# Comprehensive Analysis of N6-Methyladenosine (m^6^A) RNA Methylation Regulators and Tumour Microenvironment Cell Infiltration Involving Prognosis and Immunotherapy in Gastroesophageal Adenocarcinomas

**DOI:** 10.1155/2022/3506518

**Published:** 2022-11-21

**Authors:** Duanrui Liu, Mingjie Yuan, Zongming Wang, Liping Sun, Yusong Fang, Xiaoli Ma, Lulu Zhang, Yuanxin Xing, Jingyu Zhu, Yunyun Liu, Wenshuai Zhu, Shuqin Bao, Yanfei Jia, Yunshan Wang

**Affiliations:** ^1^Department of Clinical Laboratory, Shandong Provincial Hospital Affiliated to Shandong First Medical University, Jinan 250021, China; ^2^Research Center of Basic Medicine, Jinan Central Hospital, Shandong First Medical University, Jinan 250013, China; ^3^Department of Laboratory, Jinan Central Hospital, Shandong First Medical University, Jinan 250013, China; ^4^Department of Esophageal Surgery, Jinan Central Hospital, Shandong First Medical University, Jinan 250013, China; ^5^Department of Infectious Diseases, Jinan Central Hospital, Shandong First Medical University, Jinan 250013, China; ^6^Department of Gastroenterology, Jinan Central Hospital, Shandong First Medical University, Jinan 250013, China

## Abstract

**Objective:**

Gastroesophageal adenocarcinoma (GEA) is a high deadly and heterogeneous cancer. RNA N6-methyladenosine (m^6^A) modification plays a non-negligible role in shaping individual tumour microenvironment (TME) characterizations. However, the landscape and relationship of m^6^A modification patterns and TME cell infiltration features remain unknown in GEA.

**Methods:**

In this study, we examined the TME of GEA using assessments of the RNA-sequencing data focusing on the distinct m^6^A modification patterns from the public databases. Intrinsic patterns of m^6^A modification were evaluated for associations with clinicopathological characteristics, underlying biological pathways, tumour immune cell infiltration, oncological outcomes, and treatment responses. The expression of key m^6^A regulators and module genes was validated by qRT-PCR analysis.

**Results:**

We identified two distinct m^6^A modification patterns of GEA (cluster 1/2 subgroup), and correlated two subgroups with TME cell-infiltrating characteristics. Cluster 2 subgroup correlates with a poorer prognosis, downregulated PD-1 expression, higher risk scores, and distinct immune cell infiltration. In addition, PPI and WGCNA network analysis were integrated to identify key module genes closely related to immune infiltration of GEA to find immunotherapy markers. COL4A1 and COL5A2 in the brown module were significantly correlated to the prognosis, PD-1/L1 and CTLA-4 expression of GEA patients. Finally, a prognostic risk score was constructed using m^6^A regulator-associated signatures that represented an independent prognosis factor for GEA. Interestingly, COL5A2 expression was linked to the response to anti-PD-1 immunotherapy, m^6^A regulator expression, and risk score.

**Conclusion:**

Our work identified m^6^A RNA methylation regulators as an important class of players in the malignant progression of GEA and were associated with the complexity of the TME. COL5A2 may be the potential biomarker which contributes to predicting the response to anti-PD-1 immunotherapy and patients' prognosis.

## 1. Introduction

Gastroesophageal adenocarcinomas (GEAs) are still a major cause of cancer-related mortality worldwide [1]. Currently, the development of effective targeted therapeutics for GEA patients lags behind that for other cancers. Despite recent improvements in multidisciplinary and multimodality treatment, the overall prognosis for GEA patients remains poor, with a global 5-year survival rate lower than 30% for gastric cancer (GC) and approximately 19% for oesophageal adenocarcinoma [2]. Due to the high heterogeneity and complicated disease processes of GEA, there is still a lack of effective prognostic markers in this disease. Therefore, identifying molecular biomarkers and novel potential therapies are critical to predict the GEA patient prognosis and determine personalized treatment.

Notably, N6-methyladenosine (m^6^A), the most abundant modification on mRNAs in eukaryotes, is closely related to stem cell differentiation, immune response, embryonic development, and microRNA (miRNA) editing; it also plays an essential role in the progression of various cancers [3–7]. The m^6^A methylation levels in tumours mainly depend on the expression of m^6^A methylation regulators. m^6^A is modulated by methyltransferase complexes (“writers”), demethylases (“erasers”), and RNA-binding proteins (“readers”), which perform a series of biological functions [8]. The aberrant expression of m^6^A regulators plays a vital regulatory role in tumour progression, prognosis, and radioresistance. Li et al. [9] showed the characteristics of m^6^A RNA methylation across 33 types of cancer and speculated that the mechanism of m^6^A RNA modification might be associated with the activation or depression of some oncogenic pathways, such as the PI3K-AKT-mTOR signalling, KRAS, and P53 pathways. However, given the limited knowledge of the role of m^6^A methylation in GEA, studying the precise correlation between m^6^A-related regulator genes and its clinical prognosis is in high demand.

Immunotherapy represented by immunological checkpoint blockade (ICB, PD-1/L1, and CTLA-4) has demonstrated surprising clinical efficacy in a small number of patients with durable responses. In September 2017, the U.S. Food and Drug Administration (FDA) granted accelerated approval for pembrolizumab for the treatment of patients with recurrent, locally advanced, or metastatic gastric adenocarcinoma or GEA whose tumours expressed PD-L1 and with disease progression on or after 2 or more systemic therapies. Disappointingly, the response rates of immune checkpoint inhibitor monotherapy in GEA are approximately 10%–25% depending on the number of previous lines of chemotherapy and PD-L1 status [10]. Hence, it is important and necessary to understand the complexity of the tumour microenvironment (TME) and identify subclasses of the tumour immune microenvironment existing in the patients' tumours to predict and administer corresponding immunotherapy. Notably, several studies have indicated a special relationship between TME-infiltrating immune cells and m^6^A modification. For instance, Han et al. [11] showed that loss of YTHDF1 in classical dendritic cells enhanced the cross-presentation of tumour antigens and the cross-priming of CD8^+^ T cells in vivo, and YTHDF1 may be a potential therapeutic target in anticancer immunotherapy. Zhang et al. [12] determined three distinct m^6^A modification patterns in gastric cancer and found that the TME cell-infiltrating characteristics under these three patterns were highly consistent with the three immune phenotypes of tumours. Yang et al. [13] suggested that m^6^A demethylation by fat mass and obesity-associated protein (FTO) increases melanoma growth and decreases the response of anti-PD-1 blockade immunotherapy. Li et al. [14] showed that Alkbh5 regulated the composition of tumour-infiltrating Tregs and myeloid-derived suppressor cells and sensitized tumours to cancer immunotherapy. However, until now, the role of m^6^A regulators in the malignancy and prognosis of GEA has not been comprehensively clarified. Therefore, research focusing on m^6^A regulators is warranted to elucidate the potential regulatory mechanism of m^6^A methylation in the TME, which may reveal the potential mechanism and targets of immunotherapy.

In this study, we systematically evaluated the role of m^6^A modification, and correlated the m^6^A modification with the TME cell-infiltrating characteristics in GEA. Two GEA subtypes (cluster1/2) were determined via the consensus clustering for m^6^A regulators that stratified the prognosis of patients, different TIICs, and PD-1 expression. After WGCNA analysis, low COL5A2 expression was found to be linked to enhance response to anti-PD-1 immunotherapy. Risk score developed from three m^6^A regulator-based signatures was an independent prognostic indicator of patients with GEA. The m^6^A regulator-based risk signatures were significantly related to the immune cell infiltration levels of patients with GEA. Furthermore, we collected GEA samples to validate our key genes expression by qRT-PCR. Therefore, this study sought to provide insights into the regulatory mechanisms associated with the TME and the strategies for GEA immunotherapy.

## 2. Materials and Methods

### 2.1. Data Processing

The overall flow chart is shown in Figure S1. The mRNA (RNA-sequencing) fragments per kilobase of transcript per million fragments standardized expression data and corresponding clinicopathological features of TCGA-STAD&ESCAcohorts were retrieved for 159 GEA tissues and 39 adjacent nontumour tissues from The Cancer Genome Atlas (TCGA, http://cancergenome.nih.gov/) and 121 GEA tissues from the Gene Expression Omnibus (GEO, http://www.ncbi.nlm.nih.gov/geo/). Patients without prognostic information were excluded from the analysis. The dataset of GSE96669 was obtained using the GPL10558 platform (Illumina Human HT-12 V4.0 expression BeadChip). We utilized the limma package to conduct the normalization process, deleting the normal or repeated samples for subsequent analysis. Then, the clinicopathological parameters for included samples also were download from the TCGA database. The relevant data TCGA and GEO provided are publicly available and open source; hence, approval by a local ethics committee was not required.

### 2.2. Evaluation of Tumour-Infiltrating Immune Cells (TIICs)

CIBERSORT algorithm was applied to calculate the fractions of the 22 types of TIICs [15], which is considered better than previous deconvolution methods for the analysis of unknown mixture content and noise. We used this algorithm to statistically estimate the relative proportions of cell subpopulations from complex tissue expression profiles, making it a useful tool to estimate the abundances of special cells in the mixed tissue. In this research, we used the *R* package “CIBERSORT” to estimate the fraction of immune cells of TCGA samples, which followed by quality filtering that tumour samples with *P* < 0.05 were selected for the following analysis.

### 2.3. Generation of Immune Score, Stromal Score, and ESTIMATE Score

The ESTIMATE algorithm was exploited to infer the fraction of immune and stromal cells in tumour tissues based on gene expression signature, including the microarray expression, data sets, new microarray, as well as RNA-seq transcriptome profiles. The R script of the ESTIMATE algorithm was downloaded from the public source website (https://sourceforge.net/projects/estimateproject/). Then, we calculated the immune scores, stromal scores, and ESTIMATE scores for each sample of the TCGA dataset, respectively. The higher the respective score, the larger the ratio of the corresponding component in the TME. After we got three scores from the ESTIMATE method, we could classify the samples into high- and low-level groups according to the median score, respectively.

### 2.4. Selection of m^6^A Methylation Regulators

A total of 21 m^6^A methylation regulators were extracted from GSE96669 and TCGA database for identifying different m^6^A modification patterns mediated by m^6^A regulators in GEA. Although 21 regulators have been systematically analyzed in gastric cancer [12], they have not been systematically analyzed in GEA. These 21 m^6^A regulators included 8 writers (CBLL1, METTL3, METTL14, KIAA1429, RBM15, RBM15B, WTAP, and ZC3H13), 2 erasers (ALKBH5 and FTO), and 11 readers (ELAVL1, FMR1, HNRNPA2B1, HNRNPC, IGF2BP1, LRPPRC, YTHDC1, YTHDC2, YTHDF1, YTHDF2, and YTHDF3). Then, the correlation between the expression of these m^6^A RNA methylation regulators and different clinicopathological features were systematically evaluated.

### 2.5. Unsupervised Clustering of m^6^A Methylation Regulators

In order to further investigate the function of m^6^A RNA methylation regulators in GEA, we clustered the GEA patients into different groups by using the *R* package ConsensusClusterPlus (50 iterations, resample rate of 80%, and Pearson correlation, http://www.bioconductor.org/) based on the expression of the 21 m^6^A RNA methylation regulators [16]. The number of clusters and their stability were determined by the consensus clustering algorithm. Principal components analysis (PCA) was used with the *R* package for R v3.6.3 to study the gene expression patterns in different GEA groups.

### 2.6. Differentially Expressed Genes (DEGs)

We used *R* package “limma” with log_2_|fold-change (FC)| > 1 and adjusted *P* value <0.05 to perform differentiation analysis of the gene expression, and DEGs were generated by the comparison between GEA samples vs. adjacent noncancerous samples in TCGA and GSE96669 datasets. Venn online software (http://bioinformatics.psb.ugent.be/webtools/Venn/) was used to identify the overlapping DEGs between tumour and normal samples.

### 2.7. Weighted Gene Coexpression Network Analysis (WGCNA) of DEGs

WGCNA is a useful tool to establish the coexpression network between the gene pattern and clinical traits using the WGCNA package in *R* based on the RNA-seq data from TCGA database [17]. In the first step, we calculated a similarity matrix using biweight midcorrelation, as it is more robust to outliers. After that, a weighted adjacency matrix was defined by raising the coexpression similarity to appropriate soft-thresholding power. The best power (*β*-value) was chosen based on the criterion of approximate scale-free topology. Then, we transformed the adjacency into a topological overlap matrix (TOM) and calculated the corresponding dissimilarity to minimize the effects of noise and spurious associations. Hierarchical clustering was used to produce a hierarchical clustering tree and dynamic tree cut method to assign coexpressed genes to each module. Modules were constructed with a minimum module size of 20 genes, and highly similar modules were combined using a dissimilarity threshold of 0.25.

### 2.8. Screening Significant Modules and Functional Enrichment Analysis

In order to identify the significance of each module, gene significance (GS) was calculated using linear regression by log 10 conversion of the *p* value between gene expression and clinical features. Module eigengenes (MEs) were defined as the first principal component of each gene module and adopted as the representative of all genes in each module. Then, we calculated the correlation between gene modules and clinical traits by the WGCNA package in *R* and draw a heatmap. After obtaining these, genes in the gene modules from Gene Ontology (GO) and Kyoto Encyclopedia of Genes and Genomes (KEGG) pathway enrichment analysis were performed to observe the function of selected significant gene modules using the cluster Profiler package in *R*. Enriched terms and pathways with adjusting *P* value <0.05 were selected.

### 2.9. Protein-Protein Interaction (PPI) Network and Hub Genes Identification

For clarifying the drivers of inducing carcinogenesis in a more reliable way, PPI analysis was performed necessarily. The Retrieval of Interacting Genes (STRING) database (http://string-db.org) online tool was used to evaluate interactive relationships and generate PPI networks among the DEGs in selected gene modules. The interaction score 0.7 served as the cutoff value prior to visualization. Then, Cytoscape software (http://cytoscape.org/development_team.html) was selected to visualize the results of the PPI networks. Furthermore, CytoHubba app identifying hub objects from the complex interaction in the Cytoscape software was used to find top hub genes. Subsequently, top hub genes were selected and ranked by the maximal clique centrality (MCC) method. Afterward, to select key genes that affect the prognosis, survival data including the living status and survival time was extracted from the TCGA database. Kaplan–Meier survival curves were built to screen for genes significantly associated with the prognosis.

### 2.10. Construction of m^6^A-Related Gene Signature

Univariate Cox regression analysis of the expression of 21 m^6^A RNA methylation regulators was conducted to determine the candidate genes associated with overall survival (OS). After that, regulators associated with OS in univariate analyses were subsequently selected for the least absolute shrinkage and selection operator (LASSO) Cox regression to construct a m^6^A-related risk signature for clinical prognosis [18]. Finally, three m^6^A RNA methylation regulators with their corresponding coefficients were determined by the minimum mean cross-validated error, choosing the optimal penalty parameter *λ* related to the minimum 10-fold cross validation within the training set. The risk score of each patient with GEA in the TCGC cohort was calculated using the following formula:

(1) $$\begin{array}{c} Risk\;score\left(RS\right)=\overset{N}{\underset{i=1}{\sum }}\left(Coefi\times Xi\right)\text{,} \end{array}$$

where *Xi* is the standardized expression value of each selected m^6^A RNA methylation regulator, and Coef*i* is the corresponding coefficient of the gene. All patients were divided into low- and high-risk groups based on the median value of the risk scores. Survival curves in the high-risk and low-risk groups were estimated using the Kaplan–Meier method. In addition, the receiver operating characteristic (ROC) curves and area under the ROC curves (AUC values) were applied to access sensitivity and specificity. AUC >0.5 was considered as a significant diagnostic model.

### 2.11. Gene Set Enrichment Analysis (GSEA)

GSEA is a computational method usually used to determine whether a set of basically defined gene sets exhibit statistically significant differences between two biological states. GSEA was provided by the JAVA program with MSigDB v7.1 and downloaded from the website of Broad Institute [19]. According to the median value of RS, the samples were divided into two groups, and “c2.cp.kegg.v7.1.symbols.gmt” gene set enrichment analysis was carried out, with a *p* value <0.05 and *q*-value <0.05 as indicative of statistical significance. The enrichment pathway was visualized using the R packages “ggplot2” and “cluster Profiler.”

### 2.12. Patients and Sample Information

We totally collected 16 non-neoplastic and neoplastic samples from GEA patients who underwent surgical treatments in the Gastrointestinal Surgery Department of Jinan Central Hospital Affiliated to Shandong University from 2018 to 2020. Fresh tumour and non-neoplastic tissues were frozen and stored at −80°C that was used for PCR analysis. Clinical characteristics of the included patients are shown in Supplementary Table S1. This research was approved by the Medical Ethics Committee of Jinan Central Hospital Affiliated to Shandong University and the sample acquisition and usage was performed in accordance with the approved guidelines. Informed consent was acquired from each involved patient.

### 2.13. Quantitative Real-Time Polymerase Chain Reaction (qRT-PCR)

For evaluating the expression levels of three signature regulators and hub genes, we extracted the total RNA from clinical GEA samples by using RNA trizol reagent (CWBIO). According to the instructions of the manufacturer, cDNA synthesis was carrying out by using the reverse transcription kit (CWBIO). The qRT-PCR analysis was conducted on the LightCycler 480 Real-Time PCR System. The PCR mixtures were preheated for 5 min at 95°C, followed by 45 cycles of 95°C for 10 s, and 60°C for 45 s, and the final dissolution curve analysis was performed according to manufacturer's instruction. Related gene expression levels were calculated using the 2^−△△CT^ method and the related GAPDH mRNA expression was used as an endogenous control. Primer sequences are presented in Supplementary Table S2.

### 2.14. Statistical Analysis

Data were analyzed using the R software (version 3.6.3) and GraphPad Prism (version 6). Wilcoxon's test was used to compare the expression of m^6^A RNA methylation regulators between cancer and normal tissues. Spearman correlation analysis was performed using “corrplot” package in R. The distributions of age, sex, histological grade, and TNM stage between clusters and between risk subgroups were analyzed using the chi-square test. Wilcoxon rank sum or Kruskal–Wallis rank sum test as the significant test depending on the number of clinicopathological features and immunotherapy response for comparison. Survival curves were plotted by using the “survival” package in R. The ROC analysis was performed for the evaluation of the AUC value in the follow-up period with the “survival ROC” package. Log-rank test was used to assess statistical significance. All statistical results with *p* < 0.05 were regarded to be statistically significant.

## 3. Results

### 3.1. The Landscape of m^6^A Methylation Regulators and TIICs in GEA

**Figure 1:**
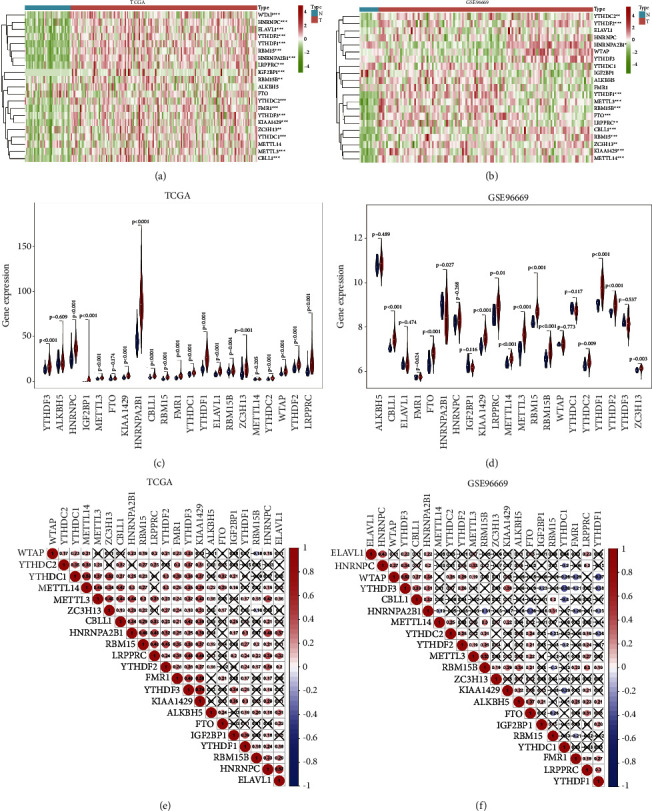
The landscape of m^6^A RNA methylation regulators in GEA. (a, b) Heatmaps of expression levels of 21 m^6^A RNA methylation regulators (normal sample vs. tumour sample) from the TCGA database (a) and GSE96669 database (b). (c, d) Violin diagrams visualizing 21 m^6^A RNA methylation regulators in GEA (assume blue is normal and red is gastric cancer) corresponding to (a, b). (e, f) Spearman correlation analysis of the 21 m^6^A regulators in GEA samples from the TCGA database (e) and GSE96669 database (f). ^*∗*^*P* < 0.05; ^*∗∗*^*P* < 0.01; ^*∗∗∗*^*P* < 0.001; N: normal sample; T: tumour sample.

To explore the important biological functions of each m^6^A RNA methylation regulator in tumourigenesis and development, we first compared the expression of 21 m^6^A methylation regulators in tumour and normal samples. The results indicated that most m^6^A RNA methylation regulators were significantly overexpressed in tumour samples of GEA patients (Figures 1(a)–1(d)). Then, correlation analysis was also employed to investigate the relationship between the expression level of m^6^A RNA methylation regulators of GEA. We found that the relationship between the 21 m^6^A RNA methylation regulators was positively correlated (Figures 1(e) and 1(f)). The analyses presented above suggested that high heterogeneity of the expressional alteration landscape in m^6^A regulators between normal and tumour samples, indicating that the expression imbalance of m^6^A regulators may play a crucial role in the GEA occurrence and progression.

Then, the difference between GEA tissues and adjacent tissues in 22 immune cell types was analyzed by using the CIBERSORT algorithm in TCGA. We first show the distribution of 22 immune cells in each GEA patient in Supplementary Figure S2A. Obviously, the proportion of immune cells in GEA tumour tissues was significantly different from that in normal tissues (Supplementary Figure S2B). We speculate that the change in the correlation of immune cells may be an internal characteristic that can reflect external differences. Then, we investigated the mutual relationship between 22 immune cells in GEA samples, and the results showed that most of the relationships between the immune cells were negatively correlated, and the M2 macrophages and naive B cells were most negatively correlated (Supplementary Figure S2C). Meanwhile, the positive correlation between resting NK cells and activated memory CD4 T cells was the most significant (Supplementary Figure S2C). The results of the above analysis indicate a complex tumour immune microenvironment, further confirming the existence of a large heterogeneity of GEA for immunotherapy.

### 3.2. Correlation of TME Components with Clinicopathological Characteristics and m^6^A Methylation Regulators

To determine the relationship between the proportion of immune and stromal components in the TME and the clinicopathological characteristics, we analyzed the corresponding clinical information of GEA cases from TCGA database. The stromal score was positively correlated with TMN-T stage (*P*=0.009), tumour grade (*P* < 0.01), and tumour stage (*P*=0.037) (Supplementary Figure S3A); immune scores were associated with advanced tumour grade (G3 > G2&G1, *P*=0.021), higher TNM-N level (N3 > N0, N1&N2, *P*=0.042), and higher immune scores in females than in males (*P*=0.045) (Supplementary Figure S3B); the ESTIMATE score showed a positive correlation with the N and T classification of the TNM stage, tumour grade, and sex (*P* < 0.05) (Supplementary Figure S3C). Therefore, these results indicated that the ratio of immune and stromal components was related to the progression of GEA, such as invasion and metastasis. Then, to explore the correlation between the high/low ratio of immune and stromal components in the TME and m^6^A regulators, we found that most m^6^A regulators were highly expressed in samples with low immune and stromal scores, which indicate a special connection between TME components and m^6^A regulators (Supplementary Figures S3D and S3E).

### 3.3. Consensus Clustering for m^6^A RNA Methylation Regulators Correlated with Distinct Survival and Immune Cell Infiltration

**Figure 2:**
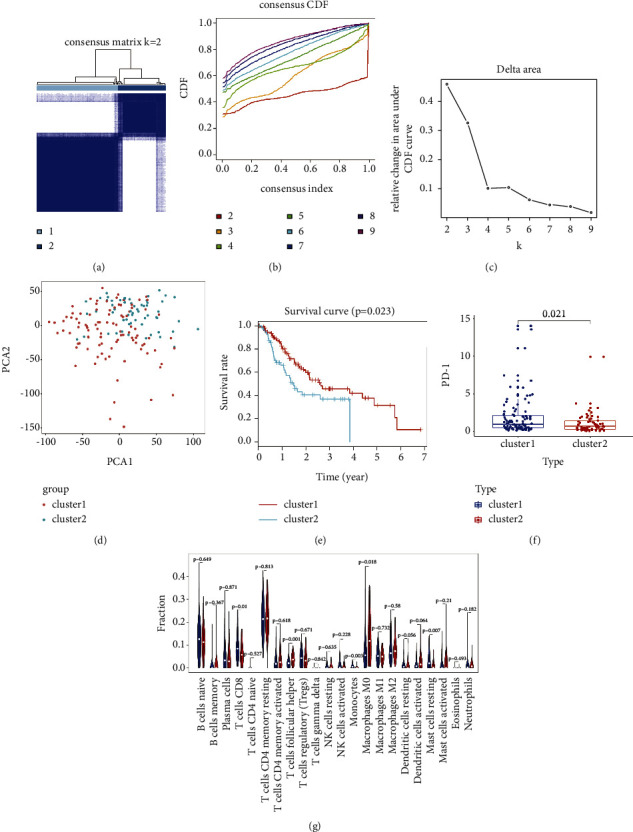
Differential overall survival, TIICs, and risk-signature regulators expression of GEA in the cluster 1/2 subgroups. (a) Consensus clustering matrix for *k* = 2. (b) Consensus clustering cumulative distribution function (CDF) for *k* = 2–9. (c) Relative change in the area under CDF curve for *k* = 2–9. (d) Principal component analysis of the total RNA expression profile in TCGA dataset. GEA in the Cluster 1 subgroup are marked with red and the Cluster 2 subgroup is marked with blue. (e) Kaplan–Meier overall survival (OS) curves for patients in the Cluster1/2 subgroup. (f) The differential expression of PD-1 between Cluster 1 and Cluster 2. (g) Vioplot visualizing differentially expressed immune cells between Cluster 1 and Cluster 2 (assume blue is Cluster 1 and red is Cluster 2). GEA: gastroesophageal adenocarcinoma.

As GEA patients have a very poor prognosis, we tried to classify patients with qualitatively different m^6^A modification patterns based on the expression of m^6^A RNA methylation regulators to explore its possible pathogenesis. According to the expression similarity of m^6^A RNA methylation regulators, *k* = 2 was the best, with clustering stability datasets increasing from *k* = 2–9 (Figures 2(a)–2(c), Supplementary Figure S4). Hence, GEA samples from TCGA dataset were preclassified into two subgroups (100 samples in one group labelled Cluster 1 and 59 samples in another subgroup labelled Cluster 2 through consensus cluster analysis. PCA was performed to elucidate the difference in transcriptional profiles between Cluster 1 and Cluster 2 subgroups. Our results showed a clear distinction between these two subgroups, which indicates the reliability of our typing (Figure 2(d)). Kaplan–Meier survival analysis for the clustered samples revealed a noticeable decrease in the OS of Cluster 2 compared with Cluster 1, suggesting that the 21 methylation regulators could classify the GEA samples at the prognostic level (Figure 2(e)). Moreover, we discovered that Cluster 2 had lower PD-1 expression and that most TIIC fractions were significantly higher in Cluster 1, such as resting CD8 T cells, monocytes, and mast cells (*P* < 0.05, Figures 2(f) and 2(g)). The clustering results suggested that patients with different modification patterns may possess different immune cell infiltration fractions and therapeutic effects.

### 3.4. Differentially Expressed Genes (DEGs) Screening and WGCNA Analysis

**Figure 3:**
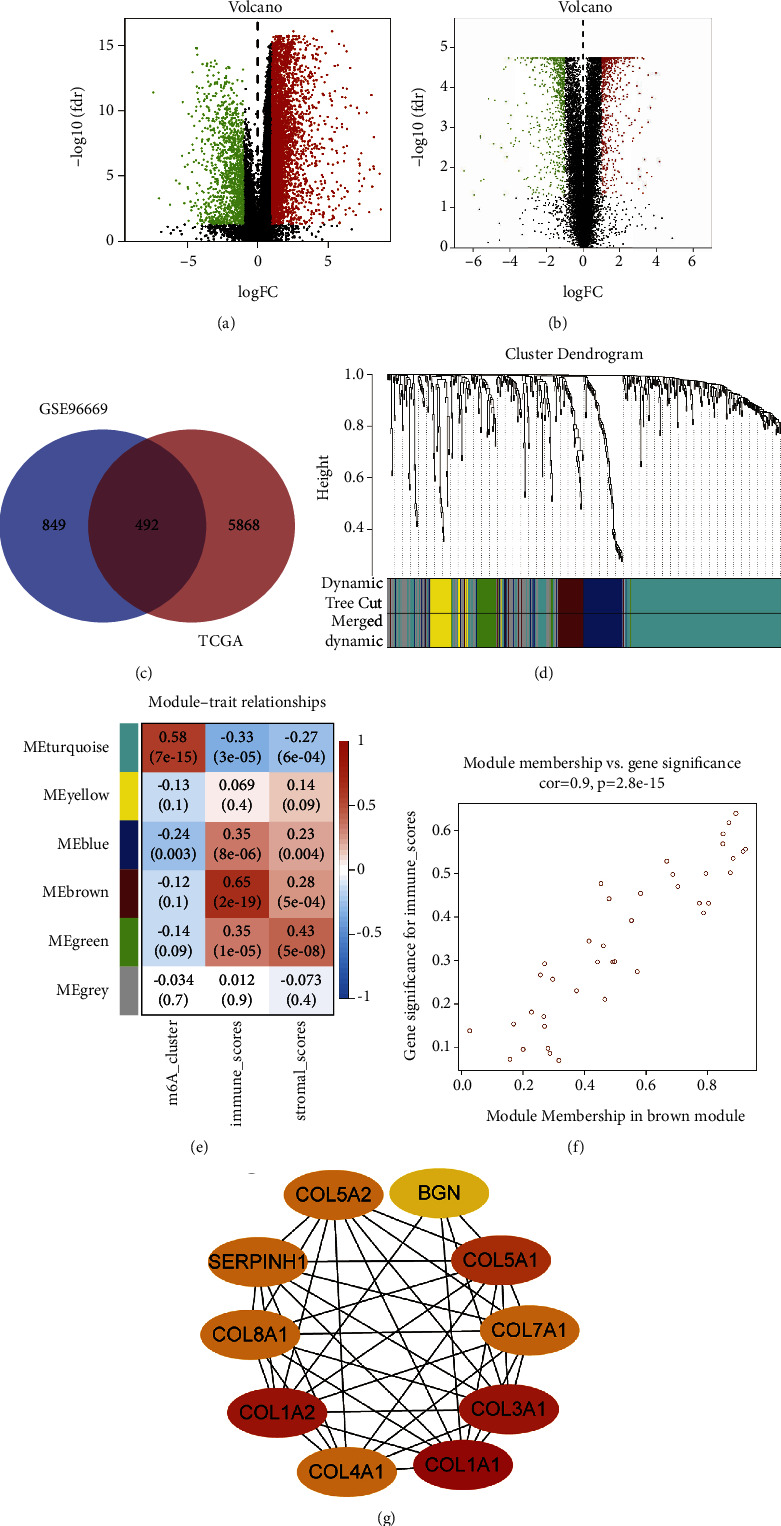
Identification of DEGs and construction of the weighted coexpression network with WGCNA. (a, b) Volcano map of DEGs in TCGA and GSE9669, respectively; green indicates downregulated genes, and red indicates upregulated genes. (c) Venn diagrams of differentially expressed genes of TCGA datasets and GSE96669 dataset. (d) The cluster dendrogram of genes of GEA patients. Each branch in the figure represents one gene, and every color represents one coexpression module. (e) Correlation between the gene module and clinical characteristics, including m^6^A cluster, immune scores, and stromal scores. The correlation coefficient in each cell represented the correlation between the gene module and the clinical characteristics, which decreased in size from red to blue. (f) Scatter diagram for module membership vs. gene significance in the brown module. (g) Identification of the hub genes from the PPI network in the brown module using maximal clique centrality (MCC) algorithm. GEA: gastroesophageal adenocarcinoma; PPI: protein-protein interaction.

Considering the diversity of immune phenotypes of GEA, we further explored potential gene biomarkers associated with immunotherapy. First, we identified 1341 DEGs in the GSE96669 dataset (Supplementary Table S3) and 6360 DEGs in the TCGA dataset (Supplementary Table S4) between tumour samples and adjacent normal samples of GEA patients (Figures 3(a) and 3(b)). Of these, 492 overlapping DEGs were selected for further analysis (Figure 3(c), Supplementary Table S5). Subsequently, WGCNA was performed to construct a gene coexpression network to correlate gene modules with trait data such as m^6^A clusters, immune scores, and stromal scores. In the case of a scale-free network and topological overlap, a hierarchical clustering tree based on dynamic hybrid cutting is established after the outlier samples were eliminated (Figure 3(d), Supplementary Figure S5A). To ensure a scale-free network, we selected *β* = 4 (scale-free *R*^2^ = 0.90) as a soft threshold (Supplementary Figure S5B). Finally, six gene modules were identified (Figure 3(e)).

**Figure 4:**
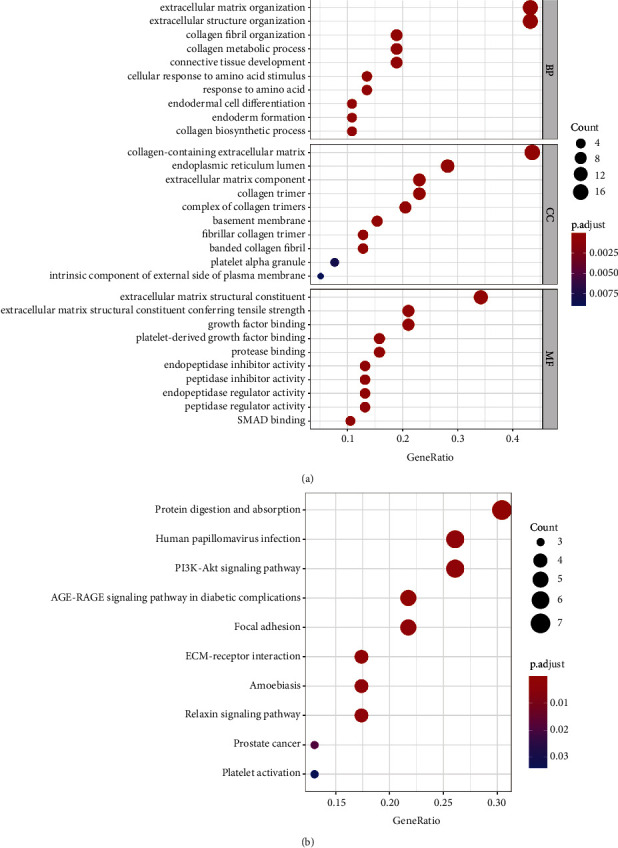
Functional enrichment analysis. (a) GO functional enrichment analysis of the genes in the brown module. (b) KEGG pathway functional enrichment analysis of the genes in the brown module. GO: gene ontology; KEGG: Kyoto Encyclopedia of Genes and Genomes.

The module trait relationships were estimated by the correlation between modules and phenotypes, which made it easier to identify highly correlated modules and phenotypes. Figure 3(e) shows that the brown module was significantly related to immune scores (cor = 0.65, *P*=2*e* − 19). In addition, scatter diagrams of gene significance are shown in Figure 3(f). To explore the function of the significant modules and key genes, GO and KEGG pathway enrichment analyses were performed. GO analysis showed that the genes in the brown module were mainly enriched in extracellular matrix organization, extracellular structure organization, and so forth (Figure 4(a)). Meanwhile, KEGG pathway enrichment analysis indicated that the genes in the brown module were mainly associated with protein digestion and absorption, the PI3K-Akt signalling pathway, and so forth (Figure 4(b)). Based on the abovementioned analysis, it is not difficult to find the most enriched pathways associated with cancer progression.

### 3.5. Hub Genes Identification and Its Role in Immunotherapy

**Figure 5:**
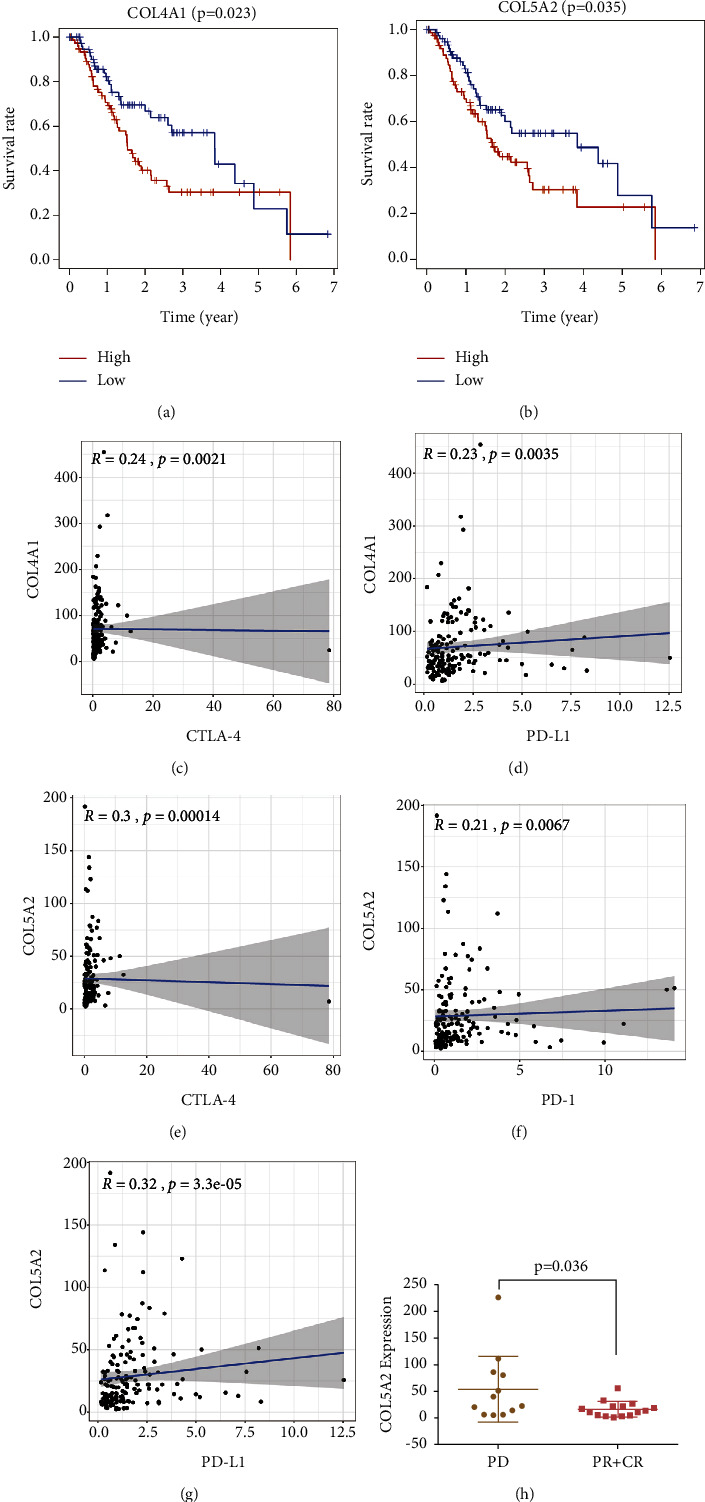
Survival analysis of hub genes and their role in immunotherapy. (a, b) Comparison of prognosis between low and high expression level of three hub genes (COL4A1 and COL5A2) using Kaplan–Meier curves. (c, g) Correlation analysis of three hub genes with immunological checkpoint (PD-1/L1 and CTLA-4) expression. (h) Distribution of COL5A2 expression in distinct anti-PD-L1 clinical response groups.

**Figure 6:**
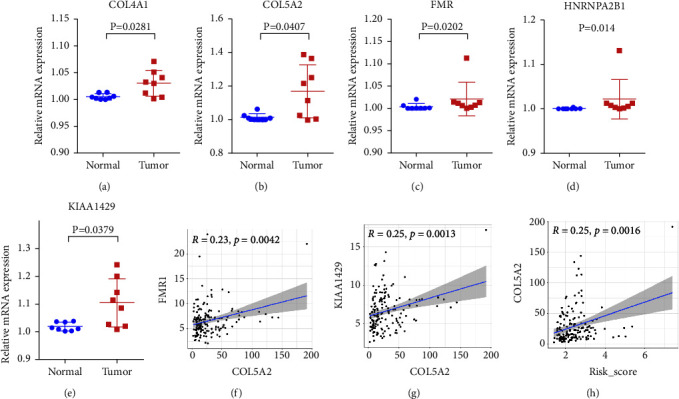
qRT-qPCR analysis and correlation analysis. (a–e) Validation of three m6A RNA methylation regulators and two hub genes in 8 pairs of GEA patient samples. (f–h) Correlation analysis between COL5A2 and FMR1, KIAA1429, risk score, respectively.

The PPI network among genes in the brown module (40 nodes and 52 edges) was established by using the STRING database. Based on the MCC scores, the top ten highest-scored genes in the brown module were selected as hub genes for further analysis (Figure 3(g)). Furthermore, two hub genes (COL4A1 and COL5A2) in the brown module were significantly negatively related to the prognosis of patients with GEA (*P* < 0.05, Figures 5(a) and 5(b)). Consistent with the above bioinformatic results, qRT-PCR analysis also revealed that COL4A1 and COL5A2 were significantly highly expressed in tumours (Figures 6(a) and 6(b)). Immunotherapies represented by PD-1/L1 and CTLA-4 blockades have undoubtedly emerged as a major breakthrough in cancer therapy. COL4A1 expression was significantly related to PD-L1 and CTLA-4 expressions (Figures 5(c), 5(d)). In addition, COL5A2 had a significant correlation with PD-1/L1 and CTLA-4 expressions (Figures 5(e)–5(g)). The above results suggested that COL4A1 and COL5A2 may be potential biomarkers for predicting the effect of immunotherapy. Then, in the anti-PD-1 cohort (GSE78220), a significant clinical response to anti-PD-1 immunotherapy in patients with low COL5A2 expression was observed compared to those with high COL5A2 expression (Figure 5(h)). Taken together, COL5A2 may be a potential biomarker contributing to predicting the response of anti-PD-1 immunotherapy.

### 3.6. Construction and Validation of Prognostic Signatures for m^6^A RNA Methylation Regulators

**Figure 7:**
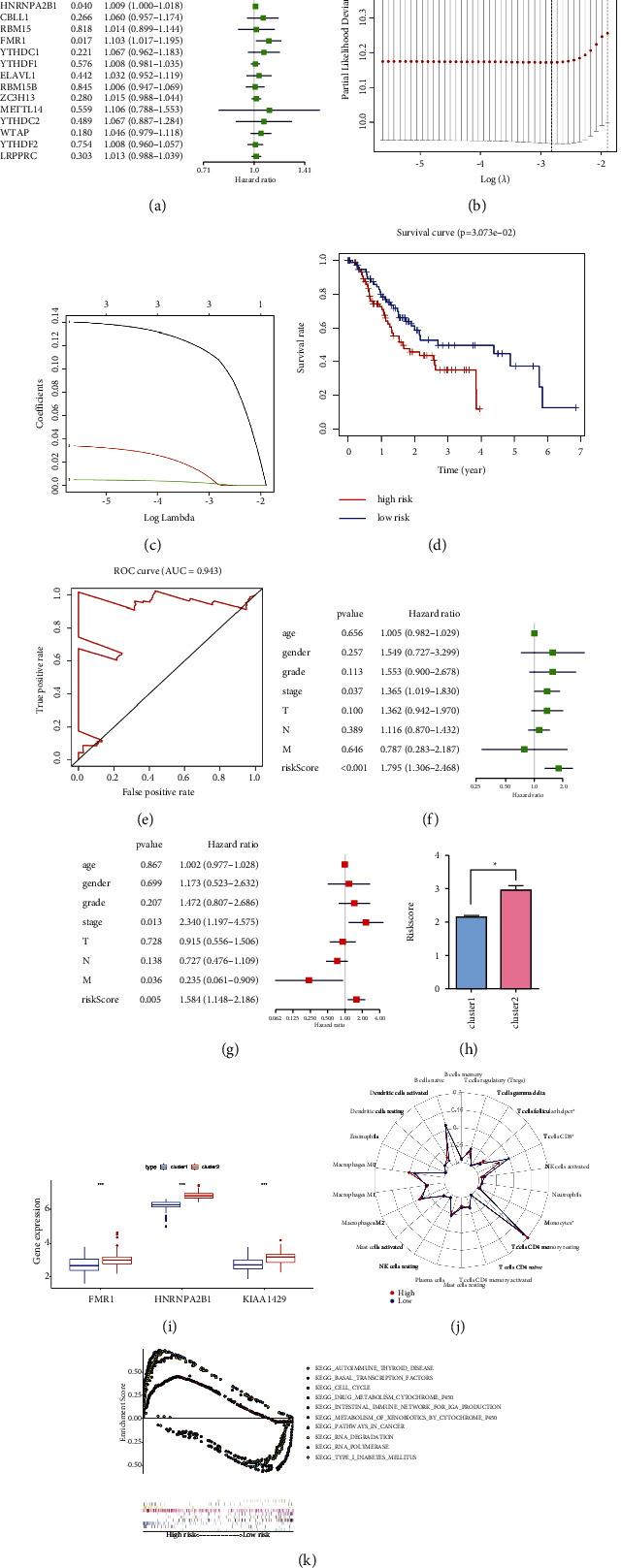
Risk signature with three m6A RNA methylation regulators. (a) The process of building the signature containing 21 m6A RNA methylation regulators. The hazard ratios (HRs), 95% confidence intervals (CIs) calculated by univariate Cox regression. (b, c) The coefficients calculated by multivariate Cox regression using LASSO is shown. (d) Kaplan–Meier overall survival (OS) curves for patients in TCGA datasets are assigned to high- and low-risk groups based on the risk score. (e) ROC curves showed the predictive efficiency of the risk signature. (f) Univariate Cox regression analyses of the correlation between clinicopathological features and overall survival of patients in TCGA datasets. (g) Multivariate Cox regression analyses of the correlation between clinicopathological features and overall survival of patients in TCGA datasets. (h) Risk score by Cluster 1/2 in TCGA cohort. (i) The differential expression of three risk-signature regulators (KIAA1429, HNRNPA2B1, and FMR1) between Cluster 1 and Cluster 2. (j) 22 different immune cells' abundance inferred by CIBERSORT for different risk groups. (k) Enrichment plots from gene set enrichment analysis (GSEA) between high- and low-risk score groups.

To investigate the prognostic value of the 21 m^6^A regulators in GEA, univariate Cox regression analysis was performed based on the expression levels of the regulators from TCGA. Our data showed that the expression of KIAA1429, HNRNPA2B1, and FMR1 is significantly correlated with the prognosis of patients. (*P* < 0.05, hazard ratio > 1, Figure 7(a)). Moreover, qRT-PCR assays revealed that the expression of KIAA1429, HNRNPA2B1, and FMR1 was significantly upregulated in tumour samples (Figures 6(c)–6(e)). To further assess the prognosis of each patient, the least absolute shrinkage and selection operator (LASSO) Cox regression analysis of the 3 prognostic regulators was conducted (Figures 7(b) and 7(c)), and the coefficient of each independent prognostic gene is shown in Supplementary Table S6. The LASSO results showed that KIAA1429, HNRNPA2B1, and FMR1 were powerful prognostic factors and a risk signature was constructed. Kaplan–Meier survival curve analysis demonstrated significant prognostic differences between the high- and low-risk groups (Figure 7(d)). Subsequently, time-dependent receiver operating characteristic (ROC) curves and areas under ROC curves (AUCs) were calculated to verify the reliability of the risk signature (AUC = 0.943, Figure 7(e)). Then, the univariate and multivariate Cox regression analysis results suggested that the risk score is an independent prognostic indicator (Figures 7(f) and 7(g)). In addition, Cluster 2 had a higher risk score and expression of KIAA1429, HNRNPA2B1, and FMR1 than Cluster 1 (Figures 7(h) and 7(i)), which was consistent with the fact that Cluster 2 had a worse prognosis than Cluster 1.

Furthermore, we evaluated the relative abundance of 22 TIICs for each patient within two risk groups using CIBERSORT. We observed a significant difference in the infiltration fraction of T follicular helper, monocyte, and CD8 T cells (Figure 7(j)). Moreover, to screen for the possible signalling pathways and mechanisms that were significantly altered within the high- and low-risk groups, GSEA was performed with data from the TCGA cohort. As shown in Figure 7(k), RNA modification-related pathways and cancer-associated pathways were more enriched in the high-risk group. Pathway enrichment analysis provided evidence of the molecular mechanisms affected by the risk signature. Importantly, risk signature gene (FMR1 and KIAA1429) expression and risk score were found to be significantly correlated with COL5A2, implicating COL5A2 as an important marker of prognosis (Figures 6(f)–6(h)).

## 4. Discussion

In the present research, we attempted to demonstrate the expression patterns, prognostic values, and effects of m^6^A regulators in GEA on the TME. Differential expression analysis found that the majority of m^6^A RNA methylation regulators were significantly differentially expressed between adjacent normal and tumour samples, suggesting that these m^6^A regulators are closely associated with cancer proliferation. Compared to normal tissues, GEA is locally infiltrated with higher immune cell subgroups, including naive B cells, memory B cells, plasma cells, gamma delta T cells, M0 macrophages, M1 macrophages, resting dendritic cells, and active dendritic cells. Meanwhile, ESTIMATE algorithm-derived immune scores, stromal scores, and ESTIMATE scores were applied to facilitate the quantification of the nontumour components in malignancy [20]. Stromal, immune, and ESTIMATE scores for tumour tissue were found to be significantly associated with the clinicopathologic features of the tumour, such as age, differentiation grade, and TNM stage. Importantly, the expression of most m^6^A regulators was significantly associated with immune/stromal scores. Therefore, these results demonstrated that aberrant immune infiltration and m^6^A regulator expression in GEA, as a tightly regulated process, which might play important roles in the tumour development and that this process has clinical importance.

We characterized the effects of distinct m^6^A methylation modifications on different GEA subtypes by clustering m^6^A regulators. The two subtypes showed significant differences in patient prognosis, PD-1 expression, immune cell infiltration, and RS. This suggests that the differences between the two subtypes are essential and reflect the heterogeneity of the immune microenvironment of GEA, which is worthy of further study. To investigate the expression characteristics of m^6^A methylation regulators in tumours, many studies clustered the tumour samples into different subtypes using consensus clustering analysis. For instance, Jing Chen et al. [21] identified two clusters of clear cell renal carcinoma with significant differences in OS and tumour stage between them based on the expression pattern of m^6^A RNA methylation regulators by means of consensus clustering. Similarly, Yi et al. [22] showed that two molecular subtypes were identified by consensus clustering for 15 m^6^A regulators, and two subtypes were distinct in the prognosis, PD-L1 expression, immunoscore, and immune cell infiltration. However, to date, the expression of m^6^A regulators has remained elusive for typing research by consensus clustering analysis in GEA. In our research, we identified a special relationship between m^6^A modification patterns and tumour immune cell infiltration.

To explore potential genetic markers to predict the effect of immunotherapy in GEA patients, we systematically clustered the coexpressed genes by WGCNA. This approach allowed us to identify gene modules most related to cancer immunological phenotypes. COL4A1 and COL5A2, the two hub prognostic genes in the brown module, in the collagen family were selected for further analysis. Désert et al. [23] reported that elevated expression of COL4A1 was significantly correlated with the tumour stage and worse overall survival in patients with hepatocellular carcinoma. Zhang et al. [24] demonstrated the abnormally high expression of COL4A1 in GC and high expression of COL4A1 was closely correlated with the primary tumour size, lymph node metastasis, and distant metastasis, with the silencing of COL4A1 significantly inhibiting cell proliferation of GC cells in vitro. Meanwhile, elevated COL4A1 gene expression has been found to be associated with trastuzumab resistance in GC [25]. Several studies have reported that COL5A2 might play a crucial role in the initiation and progression of tumours using bioinformatics technologies [26, 27]. More importantly, COL5A2 was correlated with stromal scores in GC, promoted the recruitment of circulating monocytes into the TME, and facilitated their differentiation into tumour-associated macrophages [28]. Similarly, in our research, we found that COL4A1 and COL5A2 were significantly related to the prognosis of GEA patients and TME infiltration characteristics. Intriguingly, the expression of COL5A2 and COL4A1 was significantly correlated with ICB (PD-1/L1 and CTLA-4) expression. Notably, COL5A2 expression was also linked to the response of anti-PD-1 immunotherapy. The above results suggest that COL5A2 is a potential gene marker to predict the effect of immunotherapy in GEA patients.

Whether m^6^A RNA methylation regulators have a prognostic value in cancer is of great significance [29]. We performed univariate and LASSO Cox regression analyses to construct a prognosis-related risk signature with three m^6^A RNA methylation regulators, including KIAA1429, HNRNPA2B1, and FMR1, which divided the GEA patients into low- and high-risk groups. In the m^6^A methyltransferase complex, KIAA1429 acts as a scaffold in bridging the catalytic core components of the methyltransferase complex and RNA substrates, which affect the installation of m^6^A at specific locations [30]. Miao et al. [31] found that KIAA1429 could serve as an oncogene in gastric cancer by stabilizing c-Jun mRNA in an m^6^A-independent manner. HNRNPA2B1 is a nuclear reader of the m^6^A mark and has important effects on primary microRNA processing and alternative splicing. Barceló et al. [32] reported that HNRNPA2B1 acts as a regulator of KRAS-dependent tumourigenesis through the critical pancreatic ductal adenocarcinoma cell signalling pathway PI3K/AKT. The FMR1 gene and the consequent lack of synthesis of FMR protein (FMRP) are associated with the fragile X syndrome, and FMRP plays a critical role in chromatin dynamics, RNA binding, mRNA transport, and mRNA translation [33, 34]. Li et al. [35] indicated that high expression of KIAA1429 and HNRNPA2B1 was significantly associated with the poor prognosis in osteosarcoma, and m^6^A regulators might be involved in osteosarcoma progression through a humoral immune response. Zalfa et al. [36] reported that there was an association between FMRP levels and the invasive phenotype in melanoma. In accordance with previous results, we found that the three-gene risk signature, KIAA1429, HNRNPA2B1, and FMR1, showed good performance for predicting the GEA patient prognosis and immune cell infiltration characteristics. Importantly, our further study revealed that patients in different risk groups had different levels of T-cell and macrophage infiltration. Moreover, COL5A2 expression was significantly related to KIAA1429 and FMR1 expression. Therefore, we speculate that the m^6^A modification of COL5A2 may play an important role in the immunotherapy and prognosis of GEA, which needs further validation.

The tumour microenvironment plays an essential regulatory role in tumourigenesis, and its heterogeneity can lead to multiple dimensions, including patient prognosis and therapeutic response [37–39]. Here, we analyzed the molecular signature of immune cell infiltration in different m6A RNA methylation modification patterns. Notably, CD8^+^ T cells mostly originated from normal mucosal tissues, while macrophages and Treg cells were enriched in GEA tissues. Therefore, we indicated that the downregulated immunogenicity of cancer cells potentially contributes to the formation of an immunosuppressive microenvironment. Li et al. [40] reported that a large population of CD8^+^ T cells showed continuous progression from an early effector “transitional” into a dysfunctional T-cell state, and the intensity of the dysfunctional signature was related to tumour reactivity. m^6^A RNA modification controls the differentiation of naive T cells and sustains the suppressive functions of Tregs [5, 41]. Then, our work revealed that three m^6^A regulators are highly expressed in CD8^+^ T cells, Tregs, and macrophages, which was consistent with previous works. In short, we first discovered that KIAA1429, HNRNPA2B1, and FMR1 regulate T-cell differentiation in the GEA microenvironment, which may provide new targets to optimize immunotherapy.

In summary, this study systematically evaluated the prognostic value, TME profiles, novel subtypes, and immunotherapy response in GEA patients based on m^6^A regulator expression. We also generated a risk signature to evaluate the prognosis of each GEA patient. Importantly, COL5A2 was found to be linked to the response of anti-PD-1 immunotherapy, m^6^A regulator expression, and risk score. The information from this study contributes to our understanding of m^6^A RNA regulators and the TME of GEA and may help the development of a new generation of immune therapeutics and precision treatment in GEA.

## Data Availability

The datasets used in this study are available from TCGA (https://portal.gdc.cancer.gov/repository) and GEO (http://www.ncbi.nlm.nih.gov/geo/) databases.

## Supplementary Materials

Supplementary Materials Table S1: clinical characteristics of included patients. Table S2: primers used for quantitative PCR. Table S3: differential expressed genes between GEA samples and adjacent normal samples in the GSE96669 dataset. Table S4: differential expressed genes between GEA samples and adjacent normal samples in TCGA dataset. Table S5: the overlapping differential expressed genes between GEA samples and adjacent normal samples. Table S6: the coefficient of each independent prognostic gene in the risk model. Figure S1: workflow diagram. The workflow graph of data analysis. Figure S2: the composition of tumour-infiltrating immune cells in gastroesophageal adenocarcinomas and normal samples. (A, B) The expression of 22 tumour-infiltration immune cells in gastroesophageal adenocarcinomas and normal samples. (C) Vioplot visualizing differentially expressed immune cells between gastroesophageal adenocarcinomas and normal samples (assume blue is normal and red is tumour). (D) Spearman correlation analysis of the 22 TIICs in gastroesophageal adenocarcinomas. Figure S3: correlation of TME components with clinicopathological characteristics and m^6^A methylation regulators (A–C) box plot depicting the association between stromal score, immune score, ESTIMATE score, and clinical parameters, respectively. (D) Vioplot visualizing differentially m^6^A regulators expression between high immune score group (red) and low immune score group (blue) in GEA patients. (E) Vioplot visualizing differentially m^6^A regulators expression between the high stromal score group (red) and low stromal score group (blue) in GEA patients. Figure S4: consensus clustering matrix for *k* = 2–9. Figure S5: WGCNA analysis. (A) Dendrogram of the genes and different clinical factors of GEA (m^6^A cluster, immune scores ,and stromal scores). (B) Analyze the scale-free fit index of the 1–20 soft threshold power (*β*).
