# Supplementary material for: Comprehensive Analysis of N6-Methyladenosine (m6A) RNA Methylation Regulators and Tumour Microenvironment Cell Infiltration Involving Prognosis and Immunotherapy in Gastroesophageal Adenocarcinomas
Source: Can J Gastroenterol Hepatol. 2022 Nov 21;2022:3506518. doi: 10.1155/2022/3506518 (PMC9705116; doi:10.1155/2022/3506518)
Supplement: Supplementary Materials — Table S1: clinical characteristics of included patients. Table S2: primers used for quantitative PCR. Table S3: differential expressed genes between GEA samples and adjacent normal samples in the GSE96669 dataset. Table S4: differential expressed genes between GEA samples and adjacent normal samples in TCGA dataset. Table S5: the overlapping differential expressed genes between GEA samples and adjacent normal samples. Table S6: the coefficient of each independent prognostic gene in the risk model. Figure S1: workflow diagram. The workflow graph of data analysis. Figure S2: the composition of tumour-infiltrating immune cells in gastroesophageal adenocarcinomas and normal samples. (A, B) The expression of 22 tumour-infiltration immune cells in gastroesophageal adenocarcinomas and normal samples. (C) Vioplot visualizing differentially expressed immune cells between gastroesophageal adenocarcinomas and normal samples (assume blue is normal and red is tumour). (D) Spearman correlation analysis of the 22 TIICs in gastroesophageal adenocarcinomas. Figure S3: correlation of TME components with clinicopathological characteristics and m6A methylation regulators (A–C) box plot depicting the association between stromal score, immune score, ESTIMATE score, and clinical parameters, respectively. (D) Vioplot visualizing differentially m6A regulators expression between high immune score group (red) and low immune score group (blue) in GEA patients. (E) Vioplot visualizing differentially m6A regulators expression between the high stromal score group (red) and low stromal score group (blue) in GEA patients. Figure S4: consensus clustering matrix for k = 2–9. Figure S5: WGCNA analysis. (A) Dendrogram of the genes and different clinical factors of GEA (m6A cluster, immune scores ,and stromal scores). (B) Analyze the scale-free fit index of the 1–20 soft threshold power (β). [file 3506518.f1.zip › Table S3.docx]

Table S3. Diffenertial expressed genes between GEA samples and adjacent normal samples in the GSE96669 dataset.

| gene | conMean | TreatMean | logFC | pValue | fdr | type |
| --- | --- | --- | --- | --- | --- | --- |
| ATP4A | 8739.908 | 96.94518 | -6.49431 | 0.003037 | 0.011967 | down-regulated gene |
| APOC3 | 6132.573 | 99.81388 | -5.94111 | 0.01773 | 0.048248 | down-regulated gene |
| GIF | 8789.859 | 180.3436 | -5.60702 | 0.000257 | 0.001642 | down-regulated gene |
| AQP10 | 3148.828 | 64.83225 | -5.60196 | 0.000292 | 0.001809 | down-regulated gene |
| MS4A10 | 3302.885 | 69.67445 | -5.56695 | 0.006267 | 0.021232 | down-regulated gene |
| ATP4B | 2923.889 | 68.7686 | -5.40999 | 0.005526 | 0.019199 | down-regulated gene |
| PGA4 | 4977.942 | 140.9075 | -5.14273 | 1.66E-05 | 0.000194 | down-regulated gene |
| S100G | 1644.732 | 56.20288 | -4.87106 | 0.000439 | 0.002517 | down-regulated gene |
| KCNE2 | 2725.315 | 97.53825 | -4.80431 | 1.86E-05 | 0.000211 | down-regulated gene |
| CHIA | 1346.743 | 61.23676 | -4.45893 | 0.000587 | 0.00319 | down-regulated gene |
| LIPF | 10743.01 | 533.8051 | -4.33094 | 1.19E-06 | 3.46E-05 | down-regulated gene |
| GAST | 1390.706 | 70.44671 | -4.30314 | 0.017339 | 0.0474 | down-regulated gene |
| C6orf58 | 3331.412 | 179.0497 | -4.2177 | 9.24E-07 | 3.05E-05 | down-regulated gene |
| AGXT2L1 | 1126.204 | 60.87759 | -4.20941 | 2.08E-05 | 0.000229 | down-regulated gene |
| PRSS7 | 1367.787 | 76.44412 | -4.16129 | 0.001145 | 0.005466 | down-regulated gene |
| PGA3 | 9194.334 | 537.7196 | -4.09582 | 0.000102 | 0.000788 | down-regulated gene |
| CPA2 | 1247.881 | 74.37997 | -4.06842 | 7.96E-08 | 1.84E-05 | down-regulated gene |
| ADA | 4065.129 | 263.6933 | -3.94637 | 5.48E-05 | 0.00049 | down-regulated gene |
| APOA1 | 6970.319 | 453.458 | -3.94218 | 0.000152 | 0.00108 | down-regulated gene |
| GKN1 | 10468.63 | 684.3818 | -3.93513 | 0.000412 | 0.002392 | down-regulated gene |
| GUCA2B | 1084.004 | 77.54487 | -3.80519 | 0.000528 | 0.002918 | down-regulated gene |
| GKN2 | 7988.061 | 588.5068 | -3.76271 | 0.000467 | 0.002644 | down-regulated gene |
| UGT2B17 | 1428.891 | 109.5005 | -3.70589 | 3.46E-07 | 2.04E-05 | down-regulated gene |
| ALDOB | 7478.739 | 576.7982 | -3.69666 | 0.000353 | 0.002117 | down-regulated gene |
| PGA5 | 11678.75 | 916.6409 | -3.67139 | 0.000292 | 0.001809 | down-regulated gene |
| CD36 | 853.5864 | 71.63628 | -3.57477 | 9.12E-08 | 1.84E-05 | down-regulated gene |
| SST | 991.1242 | 83.85195 | -3.56315 | 2.68E-05 | 0.000279 | down-regulated gene |
| UGT2B7 | 2274.618 | 200.9573 | -3.50066 | 0.003037 | 0.011967 | down-regulated gene |
| CCKBR | 791.7925 | 71.08184 | -3.47757 | 0.000375 | 0.002229 | down-regulated gene |
| GHRL | 3203.053 | 304.5844 | -3.39453 | 5.56E-07 | 2.41E-05 | down-regulated gene |
| GSTA1 | 3020.906 | 292.7203 | -3.36739 | 1.79E-07 | 1.84E-05 | down-regulated gene |
| NAT8B | 1279.213 | 132.7458 | -3.26852 | 0.000174 | 0.001196 | down-regulated gene |
| GC | 1543.615 | 161.29 | -3.25858 | 2.04E-07 | 1.84E-05 | down-regulated gene |
| HDC | 948.6892 | 102.8198 | -3.20582 | 1.06E-05 | 0.000141 | down-regulated gene |
| CHRD | 618.3001 | 67.18068 | -3.20219 | 0.005526 | 0.019199 | down-regulated gene |
| TMED6 | 876.9554 | 98.30123 | -3.15722 | 3.61E-07 | 2.08E-05 | down-regulated gene |
| CHGA | 3261.563 | 384.138 | -3.08587 | 7.03E-07 | 2.70E-05 | down-regulated gene |
| GPER | 928.5184 | 109.4003 | -3.08531 | 2.32E-05 | 0.000249 | down-regulated gene |
| FNDC5 | 654.0917 | 77.34874 | -3.08004 | 0.005388 | 0.018821 | down-regulated gene |
| ESRRG | 586.6513 | 71.12775 | -3.04402 | 4.37E-08 | 1.84E-05 | down-regulated gene |
| SULT2A1 | 650.8763 | 79.23078 | -3.03825 | 1.09E-07 | 1.84E-05 | down-regulated gene |
| DNASE1 | 1253.623 | 153.9978 | -3.02512 | 5.87E-05 | 0.000516 | down-regulated gene |
| MRGPRD | 597.5649 | 74.01877 | -3.01313 | 0.004866 | 0.017376 | down-regulated gene |
| AKR1B15 | 2610.224 | 328.7117 | -2.98928 | 1.31E-07 | 1.84E-05 | down-regulated gene |
| CIDEC | 1122.464 | 143.5199 | -2.96735 | 0.0095 | 0.029533 | down-regulated gene |
| CDH2 | 788.0655 | 102.0632 | -2.94885 | 0.000192 | 0.001294 | down-regulated gene |
| RBPMS2 | 1210.587 | 160.8878 | -2.91158 | 2.69E-06 | 5.60E-05 | down-regulated gene |
| DNER | 502.6477 | 68.36776 | -2.87816 | 0.000233 | 0.001516 | down-regulated gene |
| KRT20 | 1695.579 | 230.8606 | -2.87668 | 0.000102 | 0.000788 | down-regulated gene |
| CKM | 445.6079 | 60.81684 | -2.87323 | 0.000425 | 0.002454 | down-regulated gene |
| CKB | 3221.233 | 440.3751 | -2.87081 | 0.01045 | 0.031834 | down-regulated gene |
| LOC653436 | 1164.939 | 161.2077 | -2.85326 | 5.33E-07 | 2.36E-05 | down-regulated gene |
| LOC647169 | 1016.271 | 141.3401 | -2.84604 | 2.23E-07 | 1.87E-05 | down-regulated gene |
| CKMT2 | 722.7839 | 101.3382 | -2.83439 | 0.000147 | 0.001053 | down-regulated gene |
| SCTR | 840.2359 | 118.578 | -2.82496 | 4.68E-07 | 2.23E-05 | down-regulated gene |
| MS4A8B | 970.5019 | 139.7303 | -2.79609 | 6.08E-05 | 0.000529 | down-regulated gene |
| APOBEC2 | 439.0723 | 63.94075 | -2.77965 | 2.78E-07 | 1.97E-05 | down-regulated gene |
| GSTA2 | 852.5106 | 124.7533 | -2.77264 | 3.04E-07 | 1.98E-05 | down-regulated gene |
| CLCNKA | 607.8543 | 89.43225 | -2.76486 | 0.016579 | 0.045698 | down-regulated gene |
| METTL7A | 1219.513 | 182.2516 | -2.7423 | 7.26E-08 | 1.84E-05 | down-regulated gene |
| LOC100133432 | 14231.32 | 2140.11 | -2.73331 | 1.24E-06 | 3.53E-05 | down-regulated gene |
| UGT2B11 | 613.2796 | 92.8182 | -2.72407 | 1.40E-06 | 3.79E-05 | down-regulated gene |
| LOC643834 | 436.0848 | 66.11334 | -2.7216 | 0.012317 | 0.036209 | down-regulated gene |
| VSIG1 | 1258.007 | 194.3462 | -2.69444 | 0.000528 | 0.002918 | down-regulated gene |
| LOC728811 | 896.0377 | 138.5584 | -2.69307 | 0.014473 | 0.041115 | down-regulated gene |
| GSTA5 | 436.419 | 67.86976 | -2.68487 | 9.54E-08 | 1.84E-05 | down-regulated gene |
| SH3GL2 | 427.2775 | 66.7126 | -2.67914 | 0.000138 | 0.000997 | down-regulated gene |
| ADH1A | 4292.306 | 684.4225 | -2.64879 | 9.44E-07 | 3.10E-05 | down-regulated gene |
| APOBEC1 | 676.2571 | 109.0978 | -2.63195 | 0.004504 | 0.016376 | down-regulated gene |
| PGC | 16657.69 | 2709.234 | -2.62023 | 5.26E-06 | 8.66E-05 | down-regulated gene |
| LOC653308 | 673.1425 | 110.1896 | -2.61092 | 3.16E-06 | 6.24E-05 | down-regulated gene |
| KCNJ16 | 485.0105 | 79.78294 | -2.60386 | 1.50E-07 | 1.84E-05 | down-regulated gene |
| LOC642103 | 410.3797 | 68.08144 | -2.59163 | 0.014473 | 0.041115 | down-regulated gene |
| GATA5 | 524.8797 | 89.01016 | -2.55994 | 9.54E-08 | 1.84E-05 | down-regulated gene |
| MFSD4 | 397.9095 | 68.22759 | -2.54401 | 0.002133 | 0.00901 | down-regulated gene |
| MYO1A | 2829.559 | 489.2653 | -2.53189 | 0.012317 | 0.036209 | down-regulated gene |
| SLC28A2 | 575.6028 | 99.76849 | -2.52842 | 6.75E-05 | 0.00057 | down-regulated gene |
| HPGD | 1958.672 | 339.8852 | -2.52676 | 1.96E-07 | 1.84E-05 | down-regulated gene |
| P2RXL1 | 383.5828 | 67.98409 | -2.49627 | 9.20E-05 | 0.000727 | down-regulated gene |
| AADAC | 787.5136 | 140.3458 | -2.48832 | 0.000117 | 0.000879 | down-regulated gene |
| CAPN13 | 942.7905 | 168.0185 | -2.48832 | 6.33E-08 | 1.84E-05 | down-regulated gene |
| LEAP2 | 625.9566 | 111.7042 | -2.48638 | 0.010205 | 0.031254 | down-regulated gene |
| SCGB2A1 | 812.1089 | 148.9013 | -2.44732 | 0.000578 | 0.003148 | down-regulated gene |
| FBP1 | 4199.021 | 782.3951 | -2.42408 | 0.000192 | 0.001294 | down-regulated gene |
| DPCR1 | 909.3722 | 171.6358 | -2.40552 | 0.015147 | 0.042531 | down-regulated gene |
| ENPP3 | 320.8988 | 61.10222 | -2.39282 | 0.000113 | 0.000857 | down-regulated gene |
| CYP2C9 | 450.5788 | 86.01987 | -2.38904 | 0.000353 | 0.002117 | down-regulated gene |
| SLC10A2 | 380.9794 | 72.82594 | -2.38719 | 0.003469 | 0.013271 | down-regulated gene |
| LOC643959 | 360.2369 | 69.04379 | -2.38336 | 0.000125 | 0.000927 | down-regulated gene |
| SLC2A12 | 491.0395 | 94.94742 | -2.37064 | 1.79E-06 | 4.32E-05 | down-regulated gene |
| CYP3A5 | 4277.64 | 834.0462 | -2.35862 | 5.94E-06 | 9.39E-05 | down-regulated gene |
| CAPN9 | 1032.643 | 201.3611 | -2.35849 | 0.000163 | 0.001136 | down-regulated gene |
| RPRM | 351.5076 | 70.33774 | -2.32118 | 0.002802 | 0.011217 | down-regulated gene |
| AKR1B10 | 8209.492 | 1644.153 | -2.31995 | 1.87E-07 | 1.84E-05 | down-regulated gene |
| FGA | 482.0087 | 96.97527 | -2.31337 | 0.005121 | 0.018096 | down-regulated gene |
| AQP11 | 757.3567 | 153.0281 | -2.30718 | 0.00088 | 0.004416 | down-regulated gene |
| VSIG2 | 4809.531 | 974.576 | -2.30305 | 0.005668 | 0.019583 | down-regulated gene |
| ACTG2 | 5311.068 | 1080.382 | -2.29746 | 7.33E-07 | 2.75E-05 | down-regulated gene |
| LOC728473 | 857.5949 | 178.6049 | -2.26352 | 1.24E-06 | 3.53E-05 | down-regulated gene |
| MSMB | 708.8506 | 148.9229 | -2.25092 | 4.28E-05 | 0.000401 | down-regulated gene |
| LOC653778 | 1079.65 | 227.7744 | -2.24489 | 4.89E-06 | 8.25E-05 | down-regulated gene |
| CA2 | 8273.23 | 1750.826 | -2.24041 | 6.88E-07 | 2.65E-05 | down-regulated gene |
| PDIA2 | 535.2518 | 114.5416 | -2.22435 | 3.56E-06 | 6.71E-05 | down-regulated gene |
| MYH11 | 2422.379 | 522.5728 | -2.21272 | 1.52E-06 | 3.96E-05 | down-regulated gene |
| MGC13057 | 709.868 | 153.3774 | -2.21047 | 0.00099 | 0.004844 | down-regulated gene |
| AKR7A3 | 4298.928 | 929.6029 | -2.20929 | 2.04E-07 | 1.84E-05 | down-regulated gene |
| LOC648399 | 438.7599 | 95.37335 | -2.20177 | 1.14E-05 | 0.000149 | down-regulated gene |
| NKX6-2 | 301.1653 | 66.19615 | -2.18574 | 0.012033 | 0.035516 | down-regulated gene |
| LOC653261 | 325.6284 | 72.3041 | -2.17108 | 0.016956 | 0.046547 | down-regulated gene |
| SLC25A34 | 427.2376 | 95.80715 | -2.15683 | 0.000311 | 0.001908 | down-regulated gene |
| SLC25A4 | 2006.12 | 455.5399 | -2.13876 | 5.72E-06 | 9.12E-05 | down-regulated gene |
| ALDH1A1 | 6328.342 | 1451.967 | -2.12382 | 2.66E-07 | 1.95E-05 | down-regulated gene |
| PECI | 934.8032 | 215.0228 | -2.12017 | 7.18E-07 | 2.70E-05 | down-regulated gene |
| UGT2A3 | 398.4932 | 91.90905 | -2.11628 | 0.004866 | 0.017376 | down-regulated gene |
| PRPH | 303.2371 | 69.95392 | -2.11597 | 0.002381 | 0.009843 | down-regulated gene |
| SCG5 | 766.3675 | 178.3548 | -2.10329 | 0.001361 | 0.006276 | down-regulated gene |
| CBR1 | 726.0927 | 169.1462 | -2.10188 | 8.50E-07 | 2.92E-05 | down-regulated gene |
| LIFR | 318.9062 | 75.11971 | -2.08587 | 8.71E-08 | 1.84E-05 | down-regulated gene |
| TFF2 | 10160.53 | 2397.458 | -2.0834 | 6.99E-05 | 0.000587 | down-regulated gene |
| ADH1C | 2328.895 | 549.631 | -2.08311 | 5.08E-06 | 8.47E-05 | down-regulated gene |
| MAOB | 404.8012 | 98.03289 | -2.04588 | 1.65E-06 | 4.16E-05 | down-regulated gene |
| FLJ42875 | 345.4437 | 84.22885 | -2.03606 | 5.77E-08 | 1.84E-05 | down-regulated gene |
| FAM3B | 1096.767 | 268.0414 | -2.03273 | 7.26E-08 | 1.84E-05 | down-regulated gene |
| MST1 | 1718.499 | 425.4845 | -2.01397 | 0.000168 | 0.001165 | down-regulated gene |
| OAT | 2007.906 | 497.5785 | -2.0127 | 0.003469 | 0.013271 | down-regulated gene |
| GABARAPL1 | 1128.183 | 283.2111 | -1.99405 | 1.59E-06 | 4.06E-05 | down-regulated gene |
| ANXA10 | 4935.86 | 1243.911 | -1.98842 | 0.000292 | 0.001809 | down-regulated gene |
| TMEM86B | 605.1438 | 153.2097 | -1.98177 | 8.50E-07 | 2.92E-05 | down-regulated gene |
| LOC284422 | 4893.161 | 1240.606 | -1.97972 | 0.000854 | 0.004312 | down-regulated gene |
| GPT | 871.1537 | 222.9927 | -1.96593 | 5.10E-07 | 2.30E-05 | down-regulated gene |
| SLC16A10 | 579.6267 | 148.3909 | -1.96572 | 0.002447 | 0.010059 | down-regulated gene |
| C20orf56 | 409.489 | 104.8448 | -1.96557 | 3.71E-05 | 0.000359 | down-regulated gene |
| SUSD2 | 1032.237 | 264.5778 | -1.96401 | 0.000381 | 0.002262 | down-regulated gene |
| C16orf89 | 245.0133 | 62.94412 | -1.96072 | 3.04E-07 | 1.98E-05 | down-regulated gene |
| UGT2B10 | 253.1129 | 65.5536 | -1.94903 | 2.55E-07 | 1.95E-05 | down-regulated gene |
| SLC4A4 | 444.6553 | 115.1863 | -1.94872 | 8.10E-06 | 0.000116 | down-regulated gene |
| MAF | 424.3808 | 110.7159 | -1.9385 | 2.49E-05 | 0.000263 | down-regulated gene |
| ACAT1 | 2303.152 | 600.9763 | -1.93823 | 9.26E-06 | 0.000128 | down-regulated gene |
| FGG | 267.5747 | 70.02222 | -1.93406 | 0.002254 | 0.009428 | down-regulated gene |
| GHR | 238.6788 | 63.23103 | -1.91637 | 0.000138 | 0.000997 | down-regulated gene |
| SLC9A2 | 901.2778 | 241.7515 | -1.89845 | 6.06E-07 | 2.47E-05 | down-regulated gene |
| FHL1 | 609.6674 | 163.6183 | -1.89769 | 3.04E-07 | 1.98E-05 | down-regulated gene |
| ENPP5 | 394.9412 | 106.436 | -1.89165 | 0.000453 | 0.002581 | down-regulated gene |
| RAP1GAP | 3656.144 | 992.1498 | -1.88169 | 0.015849 | 0.044063 | down-regulated gene |
| TREH | 235.383 | 63.91822 | -1.88071 | 0.016211 | 0.044872 | down-regulated gene |
| C10orf116 | 5952.383 | 1625.749 | -1.87236 | 7.75E-05 | 0.000637 | down-regulated gene |
| ZNF533 | 263.4862 | 72.68858 | -1.85793 | 0.000321 | 0.001957 | down-regulated gene |
| C7orf41 | 1247.87 | 346.6165 | -1.84806 | 0.000157 | 0.001108 | down-regulated gene |
| SIGLEC11 | 207.5025 | 57.87578 | -1.8421 | 0.000805 | 0.004111 | down-regulated gene |
| CYBRD1 | 1376.835 | 386.2926 | -1.83359 | 0.000961 | 0.004731 | down-regulated gene |
| PGRMC2 | 3539.562 | 1001.923 | -1.8208 | 8.14E-08 | 1.84E-05 | down-regulated gene |
| PNPLA7 | 1144.414 | 324.0276 | -1.82042 | 2.59E-06 | 5.48E-05 | down-regulated gene |
| OKL38 | 1209.832 | 343.4407 | -1.81667 | 6.59E-07 | 2.57E-05 | down-regulated gene |
| REP15 | 345.2719 | 98.32986 | -1.81203 | 3.45E-05 | 0.000341 | down-regulated gene |
| RGMB | 301.5321 | 85.93338 | -1.81102 | 2.66E-07 | 1.95E-05 | down-regulated gene |
| ANKRD43 | 615.3906 | 175.4735 | -1.81025 | 0.000544 | 0.00299 | down-regulated gene |
| ISL1 | 786.3073 | 224.7674 | -1.80666 | 6.43E-06 | 9.91E-05 | down-regulated gene |
| SOX21 | 713.63 | 204.8308 | -1.80074 | 0.009275 | 0.028972 | down-regulated gene |
| MT1H | 1048.144 | 302.9327 | -1.79077 | 4.59E-05 | 0.000425 | down-regulated gene |
| DFNA5 | 774.5169 | 224.1171 | -1.78904 | 0.003378 | 0.012995 | down-regulated gene |
| SIDT2 | 3131.496 | 908.1281 | -1.78588 | 5.14E-08 | 1.84E-05 | down-regulated gene |
| SULT1C2 | 591.2991 | 171.6457 | -1.78445 | 0.000157 | 0.001108 | down-regulated gene |
| ACAD11 | 736.6983 | 213.9678 | -1.78368 | 0.00083 | 0.004211 | down-regulated gene |
| CGNL1 | 807.9244 | 234.7764 | -1.78293 | 0.000578 | 0.003148 | down-regulated gene |
| LOC654103 | 1627.976 | 473.1321 | -1.78276 | 0.000274 | 0.001724 | down-regulated gene |
| PDK4 | 827.8375 | 241.0249 | -1.78017 | 0.000186 | 0.001259 | down-regulated gene |
| ETFDH | 785.4857 | 229.5307 | -1.7749 | 2.78E-07 | 1.97E-05 | down-regulated gene |
| ACOX2 | 606.0055 | 177.3455 | -1.77277 | 0.001756 | 0.007684 | down-regulated gene |
| SCG2 | 391.004 | 114.4833 | -1.77205 | 6.52E-05 | 0.000556 | down-regulated gene |
| PDILT | 199.2767 | 58.52174 | -1.76773 | 2.20E-06 | 4.91E-05 | down-regulated gene |
| RIMS3 | 380.3987 | 112.4032 | -1.75883 | 0.007096 | 0.023375 | down-regulated gene |
| ARRDC4 | 345.2523 | 102.0553 | -1.7583 | 1.72E-06 | 4.24E-05 | down-regulated gene |
| GLRX | 2402.437 | 710.7476 | -1.75709 | 0.008422 | 0.026843 | down-regulated gene |
| LOC100131718 | 1937.097 | 578.6857 | -1.74304 | 1.14E-06 | 3.37E-05 | down-regulated gene |
| FLJ20273 | 1008.701 | 302.1422 | -1.7392 | 1.37E-07 | 1.84E-05 | down-regulated gene |
| CYP2C18 | 1042.408 | 312.7637 | -1.73678 | 0.001441 | 0.006554 | down-regulated gene |
| ACADM | 1583.383 | 475.8544 | -1.73442 | 3.77E-07 | 2.10E-05 | down-regulated gene |
| STX12 | 819.3695 | 248.4473 | -1.72157 | 7.95E-08 | 1.84E-05 | down-regulated gene |
| HAPLN4 | 209.3977 | 63.95913 | -1.71102 | 0.01773 | 0.048248 | down-regulated gene |
| SAT2 | 1104.884 | 340.2087 | -1.6994 | 1.43E-05 | 0.000174 | down-regulated gene |
| LOC25845 | 2240.578 | 695.2944 | -1.68818 | 0.000113 | 0.000857 | down-regulated gene |
| BRP44L | 2745.811 | 854.9716 | -1.68328 | 2.04E-07 | 1.84E-05 | down-regulated gene |
| AGPAT9 | 747.661 | 233.9691 | -1.67607 | 0.000353 | 0.002117 | down-regulated gene |
| ALDH6A1 | 632.1541 | 197.9395 | -1.67522 | 1.31E-07 | 1.84E-05 | down-regulated gene |
| C17orf91 | 489.8298 | 153.3861 | -1.67511 | 2.11E-06 | 4.79E-05 | down-regulated gene |
| FAM46C | 1474.568 | 462.6581 | -1.67227 | 6.75E-05 | 0.00057 | down-regulated gene |
| C5orf32 | 6289.938 | 1985.947 | -1.66322 | 2.32E-05 | 0.000249 | down-regulated gene |
| LOC648921 | 1114.176 | 352.7479 | -1.65927 | 1.21E-06 | 3.53E-05 | down-regulated gene |
| IQGAP2 | 1159.753 | 369.0096 | -1.65209 | 1.87E-07 | 1.84E-05 | down-regulated gene |
| KCNJ13 | 264.0423 | 84.0335 | -1.65173 | 2.04E-07 | 1.84E-05 | down-regulated gene |
| GPRC5C | 924.2198 | 295.8384 | -1.64343 | 4.34E-06 | 7.68E-05 | down-regulated gene |
| LOC650128 | 824.9185 | 264.4863 | -1.64106 | 1.29E-06 | 3.61E-05 | down-regulated gene |
| RAB17 | 2593.652 | 832.2155 | -1.63996 | 2.44E-07 | 1.93E-05 | down-regulated gene |
| CPE | 426.8361 | 136.9717 | -1.6398 | 2.92E-06 | 5.90E-05 | down-regulated gene |
| PTGR1 | 1252.991 | 402.2107 | -1.63935 | 4.93E-05 | 0.000449 | down-regulated gene |
| SULT1A2 | 410.3024 | 131.8782 | -1.63748 | 0.002583 | 0.010511 | down-regulated gene |
| SEPP1 | 791.2446 | 255.7964 | -1.62913 | 0.000934 | 0.004622 | down-regulated gene |
| PDGFD | 303.8613 | 98.31907 | -1.62787 | 6.18E-06 | 9.64E-05 | down-regulated gene |
| C18orf32 | 406.9372 | 132.7563 | -1.61603 | 3.31E-07 | 2.01E-05 | down-regulated gene |
| MOSC2 | 519.1388 | 169.533 | -1.61455 | 6.06E-07 | 2.47E-05 | down-regulated gene |
| C2orf40 | 224.5013 | 73.55926 | -1.60975 | 4.30E-07 | 2.14E-05 | down-regulated gene |
| C6orf105 | 392.0793 | 128.5716 | -1.60857 | 1.46E-06 | 3.87E-05 | down-regulated gene |
| SOSTDC1 | 361.9925 | 118.9242 | -1.60592 | 8.02E-05 | 0.000653 | down-regulated gene |
| CYP4F12 | 935.6104 | 307.4701 | -1.60546 | 0.001569 | 0.007024 | down-regulated gene |
| MAMDC2 | 219.5977 | 72.25946 | -1.6036 | 3.17E-07 | 1.98E-05 | down-regulated gene |
| WSB2 | 1044.618 | 344.9716 | -1.59843 | 3.04E-07 | 1.98E-05 | down-regulated gene |
| ACACB | 792.4411 | 261.8744 | -1.59743 | 1.25E-07 | 1.84E-05 | down-regulated gene |
| MT1M | 745.3907 | 246.8649 | -1.59428 | 0.00166 | 0.007353 | down-regulated gene |
| KIT | 287.5416 | 95.93115 | -1.5837 | 0.001684 | 0.007451 | down-regulated gene |
| PLCXD3 | 207.8221 | 69.42344 | -1.58185 | 2.92E-06 | 5.90E-05 | down-regulated gene |
| FMOD | 632.7545 | 211.4108 | -1.5816 | 0.000152 | 0.00108 | down-regulated gene |
| CTSO | 418.4723 | 140.1358 | -1.57831 | 7.22E-06 | 0.000108 | down-regulated gene |
| SSTR1 | 261.5231 | 87.59938 | -1.57795 | 5.33E-07 | 2.36E-05 | down-regulated gene |
| CTSE | 3088.796 | 1035.067 | -1.57732 | 1.38E-05 | 0.00017 | down-regulated gene |
| ENPP7 | 211.2878 | 70.89376 | -1.57548 | 7.80E-06 | 0.000114 | down-regulated gene |
| PXMP2 | 662.5697 | 222.8078 | -1.57227 | 7.18E-07 | 2.70E-05 | down-regulated gene |
| TRPV6 | 211.425 | 71.44179 | -1.56531 | 9.64E-07 | 3.10E-05 | down-regulated gene |
| UGT2B15 | 193.0951 | 66.05773 | -1.54751 | 2.66E-07 | 1.95E-05 | down-regulated gene |
| MT1G | 7817.641 | 2678.614 | -1.54525 | 0.000237 | 0.001537 | down-regulated gene |
| LOC644250 | 1775.179 | 608.7583 | -1.54402 | 1.09E-06 | 3.34E-05 | down-regulated gene |
| PGM5 | 303.2969 | 104.027 | -1.54377 | 4.12E-07 | 2.13E-05 | down-regulated gene |
| CYFIP2 | 471.7243 | 161.9075 | -1.54277 | 0.002075 | 0.008813 | down-regulated gene |
| TYRP1 | 183.8027 | 63.08891 | -1.5427 | 1.79E-07 | 1.84E-05 | down-regulated gene |
| UQCRC2 | 1275.723 | 439.9349 | -1.53595 | 3.42E-06 | 6.53E-05 | down-regulated gene |
| PTPLAD2 | 1163.658 | 401.5182 | -1.53513 | 2.69E-06 | 5.60E-05 | down-regulated gene |
| IGJ | 1151.085 | 397.5913 | -1.53364 | 1.93E-05 | 0.000217 | down-regulated gene |
| LOC647307 | 853.7007 | 294.9291 | -1.53336 | 0.000782 | 0.004024 | down-regulated gene |
| FTH1 | 1631.542 | 564.2351 | -1.53187 | 8.75E-06 | 0.000122 | down-regulated gene |
| ABHD6 | 661.8814 | 228.9853 | -1.53132 | 1.29E-06 | 3.61E-05 | down-regulated gene |
| TBC1D9 | 371.0152 | 128.6874 | -1.52761 | 6.59E-07 | 2.57E-05 | down-regulated gene |
| SULT1A1 | 1418.652 | 492.1121 | -1.52746 | 3.86E-07 | 2.11E-05 | down-regulated gene |
| CCDC125 | 1881.894 | 653.0371 | -1.52695 | 6.32E-07 | 2.52E-05 | down-regulated gene |
| LOC149620 | 194.0775 | 67.5545 | -1.52251 | 0.000321 | 0.001957 | down-regulated gene |
| C10orf11 | 267.3914 | 93.15932 | -1.52118 | 0.003854 | 0.014437 | down-regulated gene |
| HSPB7 | 202.2467 | 70.72534 | -1.51582 | 0.003657 | 0.013835 | down-regulated gene |
| AFAR3 | 624.8975 | 218.873 | -1.51353 | 2.92E-06 | 5.90E-05 | down-regulated gene |
| ZNF223 | 1165.556 | 409.063 | -1.51062 | 1.29E-06 | 3.61E-05 | down-regulated gene |
| NUCB2 | 953.2156 | 335.0304 | -1.50851 | 1.28E-05 | 0.000162 | down-regulated gene |
| C11orf92 | 209.9534 | 74.08559 | -1.5028 | 0.000133 | 0.000972 | down-regulated gene |
| CAT | 956.4917 | 337.5468 | -1.50267 | 1.24E-06 | 3.53E-05 | down-regulated gene |
| FXYD4 | 292.7149 | 103.5787 | -1.49877 | 0.003203 | 0.01247 | down-regulated gene |
| SUCLG2 | 2037.071 | 721.164 | -1.4981 | 1.87E-07 | 1.84E-05 | down-regulated gene |
| SLC9A4 | 225.6606 | 79.89446 | -1.49799 | 1.69E-06 | 4.24E-05 | down-regulated gene |
| TMEM171 | 440.5591 | 156.3262 | -1.49478 | 0.002727 | 0.01097 | down-regulated gene |
| STK32B | 226.7049 | 80.70567 | -1.49007 | 1.43E-05 | 0.000174 | down-regulated gene |
| CEP27 | 1167.285 | 417.7603 | -1.48241 | 1.34E-06 | 3.69E-05 | down-regulated gene |
| TTRAP | 270.8514 | 97.02388 | -1.48109 | 4.28E-05 | 0.000401 | down-regulated gene |
| FLJ35390 | 3121.886 | 1119.644 | -1.47938 | 1.40E-06 | 3.79E-05 | down-regulated gene |
| PHYH | 882.4661 | 316.5469 | -1.47912 | 3.46E-07 | 2.04E-05 | down-regulated gene |
| LOC100129502 | 853.6397 | 306.7547 | -1.47654 | 6.59E-07 | 2.57E-05 | down-regulated gene |
| ALCAM | 1061.333 | 381.7962 | -1.475 | 3.77E-07 | 2.10E-05 | down-regulated gene |
| MFAP5 | 378.5862 | 136.2798 | -1.47405 | 3.10E-05 | 0.000313 | down-regulated gene |
| FLJ22795 | 578.7874 | 208.7751 | -1.47108 | 0.000125 | 0.000927 | down-regulated gene |
| FLNC | 678.4354 | 245.0593 | -1.46908 | 1.38E-05 | 0.00017 | down-regulated gene |
| DISP1 | 416.6041 | 150.5283 | -1.46864 | 1.50E-07 | 1.84E-05 | down-regulated gene |
| TMEM116 | 419.2272 | 151.5404 | -1.46803 | 5.10E-07 | 2.30E-05 | down-regulated gene |
| ZNF323 | 381.6651 | 138.0247 | -1.46738 | 0.000107 | 0.000824 | down-regulated gene |
| ACSF2 | 539.581 | 195.2225 | -1.46672 | 3.61E-07 | 2.08E-05 | down-regulated gene |
| APLP1 | 232.3195 | 84.09396 | -1.46604 | 0.004504 | 0.016376 | down-regulated gene |
| SYT4 | 186.012 | 67.52315 | -1.46194 | 4.12E-07 | 2.13E-05 | down-regulated gene |
| FAM13A | 282.9698 | 102.7637 | -1.46132 | 4.30E-07 | 2.14E-05 | down-regulated gene |
| TRIM50 | 172.1997 | 63.13109 | -1.44766 | 0.000109 | 0.000834 | down-regulated gene |
| LOC729009 | 2319.313 | 850.6368 | -1.44708 | 8.02E-05 | 0.000653 | down-regulated gene |
| LOC100131989 | 1090.012 | 400.1186 | -1.44584 | 8.10E-06 | 0.000116 | down-regulated gene |
| LOC100132727 | 4761.583 | 1748.59 | -1.44525 | 2.38E-06 | 5.18E-05 | down-regulated gene |
| ITPKA | 1474.271 | 546.5784 | -1.4315 | 4.34E-06 | 7.68E-05 | down-regulated gene |
| AQP4 | 153.0378 | 56.81968 | -1.42943 | 0.001081 | 0.005208 | down-regulated gene |
| DRD5 | 171.8083 | 63.87628 | -1.42745 | 1.86E-05 | 0.000211 | down-regulated gene |
| ADI1 | 358.7364 | 133.5385 | -1.42567 | 6.06E-07 | 2.47E-05 | down-regulated gene |
| ACO2 | 665.369 | 248.0436 | -1.42356 | 3.04E-07 | 1.98E-05 | down-regulated gene |
| MAOA | 2880.963 | 1075.199 | -1.42195 | 9.09E-06 | 0.000126 | down-regulated gene |
| RDH5 | 550.126 | 205.4075 | -1.42127 | 0.001707 | 0.007505 | down-regulated gene |
| LOC653489 | 1118.819 | 419.042 | -1.41681 | 1.19E-06 | 3.46E-05 | down-regulated gene |
| FTHL8 | 1856.658 | 696.5608 | -1.41439 | 0.000102 | 0.000788 | down-regulated gene |
| GSTT1 | 1301.982 | 488.7169 | -1.41364 | 1.28E-05 | 0.000162 | down-regulated gene |
| BLVRB | 758.3917 | 284.7445 | -1.41328 | 1.49E-05 | 0.000179 | down-regulated gene |
| LOC440704 | 1438.935 | 540.5899 | -1.41239 | 4.30E-07 | 2.14E-05 | down-regulated gene |
| RCAN2 | 484.4077 | 182.3315 | -1.40966 | 4.18E-06 | 7.48E-05 | down-regulated gene |
| LOC100134159 | 5347.554 | 2016.305 | -1.40716 | 2.80E-06 | 5.75E-05 | down-regulated gene |
| FAM107A | 257.2434 | 97.10675 | -1.40549 | 6.59E-07 | 2.57E-05 | down-regulated gene |
| LOC255167 | 1737.698 | 656.0621 | -1.40527 | 8.50E-07 | 2.92E-05 | down-regulated gene |
| LOC642678 | 297.2667 | 112.3524 | -1.40373 | 1.95E-06 | 4.56E-05 | down-regulated gene |
| DTWD2 | 1164.993 | 440.6326 | -1.40267 | 1.52E-06 | 3.96E-05 | down-regulated gene |
| CYB5R1 | 1686.953 | 638.4735 | -1.40172 | 0.005253 | 0.018465 | down-regulated gene |
| NNT | 424.0755 | 160.8987 | -1.39817 | 9.81E-06 | 0.000133 | down-regulated gene |
| C9orf130 | 712.8393 | 270.8225 | -1.39623 | 1.72E-06 | 4.24E-05 | down-regulated gene |
| BCAS1 | 801.1953 | 304.5415 | -1.39552 | 0.01045 | 0.031834 | down-regulated gene |
| ODAM | 258.9324 | 98.4438 | -1.3952 | 8.75E-06 | 0.000122 | down-regulated gene |
| ADHFE1 | 205.6368 | 78.30166 | -1.39298 | 7.96E-08 | 1.84E-05 | down-regulated gene |
| HACL1 | 1414.659 | 538.721 | -1.39284 | 0.003037 | 0.011967 | down-regulated gene |
| G6PC | 191.9074 | 73.136 | -1.39176 | 0.000782 | 0.004024 | down-regulated gene |
| ANGPTL3 | 181.7869 | 69.37139 | -1.38984 | 0.002018 | 0.00862 | down-regulated gene |
| RHOU | 1898.293 | 728.0077 | -1.38268 | 3.21E-05 | 0.000321 | down-regulated gene |
| BRSK2 | 188.3248 | 72.3511 | -1.38014 | 0.002018 | 0.00862 | down-regulated gene |
| LOC286512 | 2071.341 | 795.8892 | -1.37993 | 4.70E-06 | 8.07E-05 | down-regulated gene |
| LOC653086 | 1149.262 | 441.8954 | -1.37893 | 1.26E-06 | 3.58E-05 | down-regulated gene |
| ZNF486 | 7565.64 | 2912.924 | -1.37699 | 4.43E-06 | 7.82E-05 | down-regulated gene |
| DOPEY2 | 1049.541 | 404.338 | -1.37613 | 1.96E-07 | 1.84E-05 | down-regulated gene |
| USP49 | 1747.386 | 674.8335 | -1.37259 | 5.19E-06 | 8.61E-05 | down-regulated gene |
| CCNG1 | 779.3116 | 302.0758 | -1.36729 | 3.61E-07 | 2.08E-05 | down-regulated gene |
| FLJ40722 | 431.8482 | 167.4607 | -1.3667 | 1.64E-07 | 1.84E-05 | down-regulated gene |
| LOC401098 | 1678.353 | 650.8993 | -1.36654 | 1.59E-06 | 4.06E-05 | down-regulated gene |
| UBL3 | 1135.122 | 440.516 | -1.36558 | 7.81E-07 | 2.82E-05 | down-regulated gene |
| SULT1B1 | 211.6714 | 82.37873 | -1.36148 | 7.49E-07 | 2.77E-05 | down-regulated gene |
| NFE2L2 | 1436.203 | 559.369 | -1.36039 | 1.04E-05 | 0.00014 | down-regulated gene |
| PLCL2 | 617.4119 | 241.0664 | -1.3568 | 2.20E-06 | 4.91E-05 | down-regulated gene |
| LOC389765 | 636.8601 | 248.7677 | -1.35618 | 2.03E-06 | 4.67E-05 | down-regulated gene |
| DPT | 220.5906 | 86.20383 | -1.35555 | 0.000274 | 0.001724 | down-regulated gene |
| MSRA | 605.3352 | 236.5871 | -1.35536 | 2.68E-05 | 0.000279 | down-regulated gene |
| MIR1978 | 7576.01 | 2961.445 | -1.35514 | 8.10E-06 | 0.000116 | down-regulated gene |
| ARHGAP18 | 310.6877 | 121.6977 | -1.35216 | 1.29E-06 | 3.61E-05 | down-regulated gene |
| BLZF1 | 2612.779 | 1028.25 | -1.34539 | 1.52E-06 | 3.96E-05 | down-regulated gene |
| SPON1 | 562.4336 | 221.8151 | -1.34232 | 4.13E-05 | 0.00039 | down-regulated gene |
| LRRC37B2 | 4625.495 | 1825.979 | -1.34094 | 4.34E-06 | 7.68E-05 | down-regulated gene |
| HMGCS2 | 3872.242 | 1528.736 | -1.34083 | 0.001196 | 0.005674 | down-regulated gene |
| C9orf103 | 648.3117 | 256.013 | -1.34047 | 0.000528 | 0.002918 | down-regulated gene |
| ERP27 | 265.4083 | 104.877 | -1.33952 | 0.008628 | 0.027383 | down-regulated gene |
| SCIN | 406.8115 | 160.9793 | -1.33749 | 0.002956 | 0.011718 | down-regulated gene |
| SACM1L | 769.636 | 304.7132 | -1.33672 | 1.14E-06 | 3.37E-05 | down-regulated gene |
| LOC100128062 | 2958.631 | 1172.179 | -1.33574 | 3.71E-06 | 6.93E-05 | down-regulated gene |
| MT1F | 2383.567 | 945.1815 | -1.33446 | 0.000311 | 0.001908 | down-regulated gene |
| TMEM47 | 465.0252 | 184.6708 | -1.33235 | 7.22E-06 | 0.000108 | down-regulated gene |
| LOC90586 | 1512.34 | 600.6522 | -1.33218 | 1.14E-06 | 3.37E-05 | down-regulated gene |
| ACSL1 | 512.8543 | 203.7278 | -1.33191 | 5.50E-06 | 8.89E-05 | down-regulated gene |
| LOC730130 | 219.3429 | 87.36038 | -1.32814 | 0.000805 | 0.004111 | down-regulated gene |
| NLRP8 | 1751.326 | 697.7082 | -1.32775 | 9.63E-06 | 0.000132 | down-regulated gene |
| DGKD | 380.0351 | 151.6551 | -1.32534 | 1.10E-05 | 0.000145 | down-regulated gene |
| XIST | 472.8412 | 189.2501 | -1.32106 | 0.002381 | 0.009843 | down-regulated gene |
| LOC100128893 | 234.9237 | 94.03607 | -1.32091 | 5.33E-07 | 2.36E-05 | down-regulated gene |
| FBP2 | 207.0181 | 82.9905 | -1.31874 | 2.48E-06 | 5.33E-05 | down-regulated gene |
| GRAMD1C | 182.279 | 73.07796 | -1.31864 | 3.94E-07 | 2.11E-05 | down-regulated gene |
| LOC648852 | 633.3541 | 254.3961 | -1.31594 | 6.88E-07 | 2.65E-05 | down-regulated gene |
| FLJ41603 | 374.817 | 150.9074 | -1.31252 | 7.18E-07 | 2.70E-05 | down-regulated gene |
| CYB5A | 1926.303 | 775.5791 | -1.31249 | 4.43E-06 | 7.82E-05 | down-regulated gene |
| EPHX2 | 809.3594 | 326.1986 | -1.31103 | 0.003203 | 0.01247 | down-regulated gene |
| LOC645452 | 6763.606 | 2725.978 | -1.31102 | 4.26E-06 | 7.61E-05 | down-regulated gene |
| MATN2 | 707.102 | 285.8781 | -1.30652 | 0.001034 | 0.005034 | down-regulated gene |
| NAPEPLD | 317.9778 | 128.8122 | -1.30366 | 1.25E-07 | 1.84E-05 | down-regulated gene |
| TMEM106A | 672.1502 | 273.1422 | -1.29913 | 4.89E-06 | 8.25E-05 | down-regulated gene |
| TNFSF14 | 656.2424 | 266.8592 | -1.29815 | 2.39E-06 | 5.18E-05 | down-regulated gene |
| TMEM191A | 330.3316 | 134.4872 | -1.29645 | 1.72E-06 | 4.24E-05 | down-regulated gene |
| LOC400446 | 912.9246 | 372.3645 | -1.29378 | 7.98E-07 | 2.87E-05 | down-regulated gene |
| ACTRT1 | 411.0893 | 167.7262 | -1.29334 | 2.20E-06 | 4.91E-05 | down-regulated gene |
| SERPINA5 | 324.9623 | 132.9703 | -1.28917 | 0.001756 | 0.007684 | down-regulated gene |
| ATPGD1 | 214.1962 | 87.83553 | -1.28606 | 0.001213 | 0.005727 | down-regulated gene |
| LOC400986 | 294.6739 | 121.0204 | -1.28387 | 1.93E-05 | 0.000217 | down-regulated gene |
| LEP | 478.9091 | 196.7881 | -1.28311 | 1.28E-05 | 0.000162 | down-regulated gene |
| LRIG1 | 864.9732 | 355.76 | -1.28175 | 0.002254 | 0.009428 | down-regulated gene |
| TEF | 326.2482 | 134.2681 | -1.28085 | 8.71E-08 | 1.84E-05 | down-regulated gene |
| DHRS7 | 2825.753 | 1163.61 | -1.28003 | 8.92E-06 | 0.000125 | down-regulated gene |
| PVRL3 | 1028.227 | 424.4999 | -1.27632 | 3.29E-06 | 6.38E-05 | down-regulated gene |
| LOC100132585 | 1565.473 | 646.7441 | -1.27533 | 4.52E-06 | 7.87E-05 | down-regulated gene |
| DMC1 | 3394.421 | 1402.693 | -1.27497 | 5.08E-06 | 8.47E-05 | down-regulated gene |
| BAGE5 | 194.138 | 80.23771 | -1.27473 | 4.35E-05 | 0.000408 | down-regulated gene |
| ZNF738 | 3958.436 | 1637.313 | -1.2736 | 1.16E-05 | 0.000152 | down-regulated gene |
| HEXDC | 344.2323 | 142.4373 | -1.27306 | 9.09E-06 | 0.000126 | down-regulated gene |
| FLJ36131 | 865.6982 | 358.74 | -1.27093 | 2.80E-06 | 5.75E-05 | down-regulated gene |
| PMM1 | 1139.849 | 472.3549 | -1.2709 | 6.06E-07 | 2.47E-05 | down-regulated gene |
| FAM153B | 365.4625 | 151.6124 | -1.26934 | 9.64E-07 | 3.10E-05 | down-regulated gene |
| RERGL | 232.6773 | 96.58311 | -1.26849 | 9.81E-06 | 0.000133 | down-regulated gene |
| LOC100129201 | 327.5991 | 135.9906 | -1.26842 | 7.49E-07 | 2.77E-05 | down-regulated gene |
| LOC642934 | 866.2788 | 359.6123 | -1.26839 | 1.54E-05 | 0.000184 | down-regulated gene |
| SSTR2 | 582.4286 | 241.8263 | -1.26811 | 2.20E-06 | 4.91E-05 | down-regulated gene |
| NR2F2 | 457.8149 | 190.1791 | -1.26741 | 7.49E-05 | 0.00062 | down-regulated gene |
| CPNE6 | 166.0007 | 69.01691 | -1.26617 | 8.02E-05 | 0.000653 | down-regulated gene |
| LOC100134868 | 1034.83 | 430.4831 | -1.26536 | 1.65E-06 | 4.16E-05 | down-regulated gene |
| NCOA4 | 7107.839 | 2958.421 | -1.26458 | 4.43E-06 | 7.82E-05 | down-regulated gene |
| GLOD4 | 1387.469 | 579.0699 | -1.26065 | 7.65E-07 | 2.82E-05 | down-regulated gene |
| SDHD | 1344.66 | 561.4773 | -1.25994 | 1.06E-05 | 0.000141 | down-regulated gene |
| C1orf115 | 957.2036 | 399.7182 | -1.25984 | 0.000261 | 0.001667 | down-regulated gene |
| LOC100130053 | 1316.103 | 551.5633 | -1.25467 | 9.44E-06 | 0.000129 | down-regulated gene |
| FTHL12 | 4060.724 | 1703.012 | -1.25365 | 0.000168 | 0.001165 | down-regulated gene |
| RAB33B | 373.6348 | 156.9733 | -1.25111 | 2.15E-05 | 0.000236 | down-regulated gene |
| FAM175A | 2144.173 | 903.0043 | -1.24762 | 1.72E-06 | 4.24E-05 | down-regulated gene |
| MYO5B | 322.604 | 136.0338 | -1.2458 | 1.09E-06 | 3.34E-05 | down-regulated gene |
| RNF128 | 416.9631 | 175.8438 | -1.24563 | 1.03E-06 | 3.24E-05 | down-regulated gene |
| FAM73A | 540.3076 | 228.3694 | -1.24241 | 5.56E-07 | 2.41E-05 | down-regulated gene |
| LOC643882 | 359.5893 | 152.1592 | -1.24077 | 9.64E-07 | 3.10E-05 | down-regulated gene |
| C20orf127 | 624.6164 | 264.4234 | -1.24012 | 0.00018 | 0.001226 | down-regulated gene |
| LOC100128460 | 273.8351 | 115.9335 | -1.24001 | 8.86E-07 | 2.98E-05 | down-regulated gene |
| LOC727908 | 332.9376 | 141.0764 | -1.23878 | 3.42E-06 | 6.53E-05 | down-regulated gene |
| HADH | 2704.817 | 1146.461 | -1.23834 | 1.79E-06 | 4.32E-05 | down-regulated gene |
| COX7B | 2667.714 | 1131.338 | -1.23757 | 3.39E-05 | 0.000336 | down-regulated gene |
| IL11RA | 304.102 | 129.0467 | -1.23666 | 8.33E-08 | 1.84E-05 | down-regulated gene |
| KLHDC2 | 1373.561 | 583.8569 | -1.23423 | 3.17E-07 | 1.98E-05 | down-regulated gene |
| C2orf69 | 5264.153 | 2237.635 | -1.23423 | 8.42E-06 | 0.00012 | down-regulated gene |
| SNAPC1 | 395.0269 | 167.9256 | -1.23413 | 4.18E-06 | 7.48E-05 | down-regulated gene |
| EIF5A2 | 239.0507 | 101.6866 | -1.23319 | 0.011217 | 0.033626 | down-regulated gene |
| GPR1 | 634.7649 | 270.152 | -1.23245 | 1.26E-06 | 3.58E-05 | down-regulated gene |
| FLJ25363 | 745.766 | 317.4535 | -1.23218 | 5.50E-06 | 8.89E-05 | down-regulated gene |
| LOC727899 | 250.5557 | 106.6957 | -1.23163 | 2.40E-05 | 0.000257 | down-regulated gene |
| VILL | 536.1348 | 228.3818 | -1.23115 | 0.000496 | 0.002774 | down-regulated gene |
| FAM40B | 413.3098 | 176.0699 | -1.23108 | 2.59E-06 | 5.48E-05 | down-regulated gene |
| ZNF467 | 370.2574 | 157.8212 | -1.23024 | 0.005961 | 0.020392 | down-regulated gene |
| EVI5 | 751.961 | 320.639 | -1.22971 | 5.80E-07 | 2.43E-05 | down-regulated gene |
| TMEM25 | 193.5535 | 82.74711 | -1.22595 | 7.49E-07 | 2.77E-05 | down-regulated gene |
| CCBE1 | 824.0691 | 352.522 | -1.22505 | 2.24E-06 | 4.98E-05 | down-regulated gene |
| ATP11B | 375.0383 | 160.4462 | -1.22495 | 1.66E-05 | 0.000194 | down-regulated gene |
| ANKRD9 | 1190.85 | 509.6731 | -1.22435 | 1.33E-05 | 0.000165 | down-regulated gene |
| ECHDC2 | 2871.87 | 1229.266 | -1.22419 | 3.86E-07 | 2.11E-05 | down-regulated gene |
| FAM162A | 518.0878 | 222.5346 | -1.21917 | 0.000353 | 0.002117 | down-regulated gene |
| SDPR | 302.2234 | 130.0475 | -1.21658 | 3.04E-06 | 6.06E-05 | down-regulated gene |
| LOC730990 | 492.7657 | 212.1583 | -1.21576 | 6.43E-06 | 9.91E-05 | down-regulated gene |
| IL17RD | 700.4359 | 301.6332 | -1.21546 | 4.34E-06 | 7.68E-05 | down-regulated gene |
| C6orf81 | 159.0592 | 68.50914 | -1.2152 | 0.000961 | 0.004731 | down-regulated gene |
| BCHE | 163.9517 | 70.64803 | -1.21455 | 8.71E-08 | 1.84E-05 | down-regulated gene |
| LOC731789 | 219.2583 | 94.49778 | -1.21428 | 4.89E-06 | 8.25E-05 | down-regulated gene |
| SEC24D | 404.8287 | 174.521 | -1.21391 | 5.87E-05 | 0.000516 | down-regulated gene |
| FLJ46309 | 4091.294 | 1765.884 | -1.21217 | 3.42E-06 | 6.53E-05 | down-regulated gene |
| SLC17A8 | 132.6432 | 57.28978 | -1.2112 | 0.001963 | 0.008424 | down-regulated gene |
| ALS2CR14 | 320.185 | 138.3113 | -1.21099 | 5.10E-07 | 2.30E-05 | down-regulated gene |
| AIRE | 3207.558 | 1385.983 | -1.21057 | 8.15E-07 | 2.88E-05 | down-regulated gene |
| LOC100128084 | 8718.644 | 3767.92 | -1.21034 | 6.18E-06 | 9.64E-05 | down-regulated gene |
| SEC24A | 269.1693 | 116.3394 | -1.21017 | 1.86E-05 | 0.000211 | down-regulated gene |
| LOC647081 | 451.753 | 195.6472 | -1.20728 | 6.18E-06 | 9.64E-05 | down-regulated gene |
| TMSL3 | 3796.472 | 1647.275 | -1.20458 | 0.000102 | 0.000788 | down-regulated gene |
| FTHL2 | 1517.77 | 659.2256 | -1.20311 | 0.000117 | 0.000879 | down-regulated gene |
| C5 | 422.3274 | 183.5909 | -1.20187 | 1.43E-05 | 0.000174 | down-regulated gene |
| DUXAP3 | 3602.449 | 1569.846 | -1.19836 | 3.63E-06 | 6.84E-05 | down-regulated gene |
| ENPP2 | 344.6628 | 150.2026 | -1.19828 | 1.73E-05 | 0.0002 | down-regulated gene |
| IRX3 | 906.2922 | 395.0739 | -1.19785 | 0.011755 | 0.034892 | down-regulated gene |
| PSCDBP | 243.9189 | 106.3819 | -1.19715 | 1.50E-07 | 1.84E-05 | down-regulated gene |
| FTHL3 | 1173.519 | 512.7548 | -1.1945 | 0.000218 | 0.001437 | down-regulated gene |
| CCDC106 | 502.8307 | 219.844 | -1.19359 | 2.78E-05 | 0.000288 | down-regulated gene |
| GCNT1 | 628.9689 | 275.0146 | -1.19348 | 3.16E-05 | 0.000318 | down-regulated gene |
| GBP3 | 242.0026 | 105.8724 | -1.1927 | 3.45E-05 | 0.000341 | down-regulated gene |
| PIGR | 4988.093 | 2182.835 | -1.19229 | 0.008839 | 0.027907 | down-regulated gene |
| GREM2 | 160.8225 | 70.57987 | -1.18814 | 1.05E-06 | 3.26E-05 | down-regulated gene |
| PLEKHH1 | 347.6526 | 152.6335 | -1.18757 | 5.56E-07 | 2.41E-05 | down-regulated gene |
| DBT | 531.5267 | 233.4446 | -1.18706 | 3.51E-05 | 0.000346 | down-regulated gene |
| REEP1 | 200.7952 | 88.25975 | -1.1859 | 4.70E-06 | 8.07E-05 | down-regulated gene |
| LOC644642 | 283.9154 | 124.8001 | -1.18584 | 6.88E-07 | 2.65E-05 | down-regulated gene |
| LCOR | 957.6517 | 421.348 | -1.18449 | 1.43E-05 | 0.000174 | down-regulated gene |
| PCDHB9 | 292.1164 | 128.7028 | -1.1825 | 3.42E-06 | 6.53E-05 | down-regulated gene |
| SLC9A1 | 2902.82 | 1282.891 | -1.17806 | 2.75E-06 | 5.70E-05 | down-regulated gene |
| SMCR5 | 380.2513 | 168.4571 | -1.17457 | 1.79E-06 | 4.32E-05 | down-regulated gene |
| AUH | 409.6738 | 181.5206 | -1.17434 | 4.43E-05 | 0.000413 | down-regulated gene |
| PER3 | 298.9389 | 132.6229 | -1.17252 | 1.43E-07 | 1.84E-05 | down-regulated gene |
| TMEM135 | 432.2959 | 192.1911 | -1.16948 | 2.78E-05 | 0.000288 | down-regulated gene |
| BNIP3 | 773.7924 | 344.18 | -1.16878 | 0.000211 | 0.001399 | down-regulated gene |
| PPFIBP2 | 1564.498 | 696.5052 | -1.16749 | 1.57E-07 | 1.84E-05 | down-regulated gene |
| PPA2 | 1942.711 | 865.247 | -1.16689 | 9.64E-07 | 3.10E-05 | down-regulated gene |
| ALPP | 4649.754 | 2071.352 | -1.16658 | 8.10E-06 | 0.000116 | down-regulated gene |
| IBTK | 928.4347 | 413.7204 | -1.16614 | 2.32E-05 | 0.000249 | down-regulated gene |
| SORBS2 | 258.8724 | 115.4184 | -1.16537 | 1.19E-05 | 0.000153 | down-regulated gene |
| FTHL11 | 1966.943 | 877.5639 | -1.16438 | 0.000109 | 0.000834 | down-regulated gene |
| ATP5C1 | 1670.584 | 746.3961 | -1.16234 | 6.08E-05 | 0.000529 | down-regulated gene |
| DDX17 | 673.1797 | 301.6379 | -1.15817 | 8.75E-06 | 0.000122 | down-regulated gene |
| KIAA1751 | 539.0061 | 241.6471 | -1.1574 | 1.19E-06 | 3.46E-05 | down-regulated gene |
| ABCC5 | 390.4877 | 175.4709 | -1.15405 | 1.86E-05 | 0.000211 | down-regulated gene |
| C2orf55 | 393.5408 | 177.1114 | -1.15186 | 3.56E-06 | 6.71E-05 | down-regulated gene |
| RALGPS1 | 542.4665 | 244.5154 | -1.14961 | 1.29E-06 | 3.61E-05 | down-regulated gene |
| C5orf53 | 374.616 | 169.0049 | -1.14835 | 1.96E-07 | 1.84E-05 | down-regulated gene |
| LOC100190938 | 10080.17 | 4551.159 | -1.14721 | 1.87E-06 | 4.44E-05 | down-regulated gene |
| C1orf63 | 423.3908 | 191.4749 | -1.14483 | 2.59E-05 | 0.000271 | down-regulated gene |
| FLJ21986 | 449.072 | 203.8624 | -1.13935 | 8.10E-06 | 0.000116 | down-regulated gene |
| DACT3 | 260.3507 | 118.2129 | -1.13907 | 3.86E-06 | 7.11E-05 | down-regulated gene |
| SYTL2 | 683.9528 | 310.6661 | -1.13853 | 0.006267 | 0.021232 | down-regulated gene |
| C14orf159 | 782.8215 | 355.614 | -1.13837 | 6.18E-06 | 9.64E-05 | down-regulated gene |
| FUT9 | 149.0001 | 67.80067 | -1.13594 | 6.94E-06 | 0.000105 | down-regulated gene |
| KIAA1191 | 1061.253 | 483.3013 | -1.13477 | 0.00166 | 0.007353 | down-regulated gene |
| MBD4 | 1600.033 | 729.0566 | -1.134 | 3.16E-06 | 6.24E-05 | down-regulated gene |
| LOC339352 | 255.3461 | 116.3982 | -1.13339 | 7.81E-07 | 2.82E-05 | down-regulated gene |
| ANKRD33 | 1443.344 | 657.9484 | -1.13337 | 5.29E-06 | 8.66E-05 | down-regulated gene |
| FLJ20920 | 290.2258 | 132.4237 | -1.13201 | 3.77E-07 | 2.10E-05 | down-regulated gene |
| CA8 | 225.5278 | 102.9611 | -1.13121 | 0.003203 | 0.01247 | down-regulated gene |
| C5orf28 | 1272.411 | 581.1257 | -1.13064 | 1.33E-05 | 0.000165 | down-regulated gene |
| MOCS1 | 263.9564 | 120.7105 | -1.12875 | 1.33E-05 | 0.000165 | down-regulated gene |
| LOC100130276 | 235.9173 | 107.918 | -1.12835 | 3.46E-07 | 2.04E-05 | down-regulated gene |
| KAT2B | 720.2981 | 329.5734 | -1.12799 | 4.89E-06 | 8.25E-05 | down-regulated gene |
| ACAA1 | 1064.384 | 487.4302 | -1.12675 | 0.013984 | 0.040069 | down-regulated gene |
| ASAH2 | 151.0026 | 69.16416 | -1.12648 | 2.03E-06 | 4.67E-05 | down-regulated gene |
| SELENBP1 | 751.2738 | 344.1968 | -1.12611 | 1.29E-06 | 3.61E-05 | down-regulated gene |
| SNX24 | 186.0364 | 85.3026 | -1.12492 | 2.33E-07 | 1.91E-05 | down-regulated gene |
| PRSS23 | 1011.394 | 463.9323 | -1.12436 | 0.001304 | 0.006082 | down-regulated gene |
| PAQR7 | 506.6427 | 232.8676 | -1.12146 | 0.0107 | 0.032437 | down-regulated gene |
| LOC100128525 | 218.1751 | 100.3456 | -1.12051 | 1.65E-06 | 4.16E-05 | down-regulated gene |
| RELL1 | 256.8862 | 118.1932 | -1.11998 | 1.14E-06 | 3.37E-05 | down-regulated gene |
| C8orf45 | 2087.259 | 960.7082 | -1.11944 | 5.39E-06 | 8.81E-05 | down-regulated gene |
| LOC728105 | 404.6864 | 186.2734 | -1.11938 | 4.34E-06 | 7.68E-05 | down-regulated gene |
| UBE4A | 626.9719 | 288.5911 | -1.11937 | 5.08E-06 | 8.47E-05 | down-regulated gene |
| DAPP1 | 3562.027 | 1640.176 | -1.11885 | 6.18E-06 | 9.64E-05 | down-regulated gene |
| SUCLA2 | 562.2621 | 258.9665 | -1.11848 | 2.23E-05 | 0.000242 | down-regulated gene |
| CYP2C19 | 155.0448 | 71.46082 | -1.11746 | 0.00018 | 0.001226 | down-regulated gene |
| LOC100133516 | 1456.284 | 671.3648 | -1.11712 | 1.06E-05 | 0.000141 | down-regulated gene |
| LOC100129211 | 1129.065 | 520.6744 | -1.11667 | 1.87E-06 | 4.44E-05 | down-regulated gene |
| LOC202781 | 1616.949 | 746.186 | -1.11567 | 1.06E-05 | 0.000141 | down-regulated gene |
| LOC650200 | 213.2488 | 98.45991 | -1.11493 | 7.49E-05 | 0.00062 | down-regulated gene |
| LOC100128098 | 688.0239 | 317.8076 | -1.11431 | 4.34E-06 | 7.68E-05 | down-regulated gene |
| SORD | 260.425 | 120.447 | -1.11247 | 0.002254 | 0.009428 | down-regulated gene |
| ISOC1 | 694.3171 | 321.3669 | -1.11137 | 2.69E-06 | 5.60E-05 | down-regulated gene |
| LGALS3 | 2345.202 | 1085.855 | -1.11088 | 0.000736 | 0.003833 | down-regulated gene |
| FRK | 284.6807 | 131.8413 | -1.11054 | 3.86E-07 | 2.11E-05 | down-regulated gene |
| TDRD1 | 477.2129 | 221.1107 | -1.10986 | 4.12E-07 | 2.13E-05 | down-regulated gene |
| STMN2 | 158.5736 | 73.50212 | -1.10929 | 0.000578 | 0.003148 | down-regulated gene |
| FH | 1865.191 | 864.6822 | -1.10908 | 2.08E-05 | 0.000229 | down-regulated gene |
| CLOCK | 280.0006 | 129.8149 | -1.10897 | 1.95E-06 | 4.56E-05 | down-regulated gene |
| PANK1 | 352.2828 | 163.4126 | -1.10822 | 0.000183 | 0.001245 | down-regulated gene |
| FAM190B | 337.6963 | 156.7519 | -1.10724 | 6.06E-07 | 2.47E-05 | down-regulated gene |
| RUNDC3B | 224.1939 | 104.0955 | -1.10684 | 0.004742 | 0.017045 | down-regulated gene |
| C6orf85 | 851.9087 | 395.553 | -1.10683 | 1.06E-05 | 0.000141 | down-regulated gene |
| FLJ44124 | 5721.856 | 2657.555 | -1.10638 | 3.77E-05 | 0.000365 | down-regulated gene |
| HADHB | 804.6318 | 374.216 | -1.10446 | 1.30E-05 | 0.000164 | down-regulated gene |
| SFRP5 | 123.0551 | 57.32939 | -1.10196 | 0.010205 | 0.031254 | down-regulated gene |
| AHCYL2 | 701.1999 | 327.0839 | -1.10017 | 4.93E-05 | 0.000449 | down-regulated gene |
| IL10 | 2054.044 | 959.0281 | -1.09882 | 1.10E-05 | 0.000145 | down-regulated gene |
| LRRC17 | 153.0589 | 71.51727 | -1.09772 | 3.29E-06 | 6.38E-05 | down-regulated gene |
| FAHD2A | 205.2589 | 95.95897 | -1.09695 | 1.20E-07 | 1.84E-05 | down-regulated gene |
| LOC649841 | 554.5229 | 259.2615 | -1.09684 | 0.000174 | 0.001196 | down-regulated gene |
| GCNT2 | 184.7171 | 86.36325 | -1.09683 | 1.14E-07 | 1.84E-05 | down-regulated gene |
| RETSAT | 985.452 | 460.9671 | -1.09612 | 0.003289 | 0.012712 | down-regulated gene |
| NDUFB10 | 2921.831 | 1368.383 | -1.0944 | 1.87E-07 | 1.84E-05 | down-regulated gene |
| LOC730060 | 264.2288 | 123.8495 | -1.0932 | 1.79E-06 | 4.32E-05 | down-regulated gene |
| LOC100132391 | 3257.595 | 1527.682 | -1.09246 | 1.35E-05 | 0.000168 | down-regulated gene |
| LOC730313 | 6297.354 | 2955.704 | -1.09124 | 5.50E-06 | 8.89E-05 | down-regulated gene |
| FRMD1 | 120.9949 | 56.83978 | -1.08997 | 3.17E-07 | 1.98E-05 | down-regulated gene |
| CMTM4 | 462.9711 | 217.6915 | -1.08864 | 3.58E-05 | 0.000349 | down-regulated gene |
| SGK1 | 1339.103 | 629.7998 | -1.0883 | 0.000855 | 0.004312 | down-regulated gene |
| DKFZp434K191 | 363.2506 | 170.9385 | -1.08749 | 5.29E-06 | 8.66E-05 | down-regulated gene |
| AK3 | 2487.923 | 1171.808 | -1.08621 | 7.81E-07 | 2.82E-05 | down-regulated gene |
| SLCO2A1 | 926.07 | 436.2649 | -1.08592 | 4.76E-05 | 0.000437 | down-regulated gene |
| PKIB | 205.642 | 96.88767 | -1.08575 | 3.86E-06 | 7.11E-05 | down-regulated gene |
| RAXL1 | 811.6569 | 382.862 | -1.08405 | 9.81E-06 | 0.000133 | down-regulated gene |
| CPOX | 504.0287 | 237.9066 | -1.08311 | 0.000121 | 0.000904 | down-regulated gene |
| MIR1227 | 139.2385 | 65.75264 | -1.08244 | 3.45E-05 | 0.000341 | down-regulated gene |
| SYNM | 240.6549 | 113.6918 | -1.08184 | 1.59E-06 | 4.06E-05 | down-regulated gene |
| EIF2AK4 | 901.4441 | 426.0264 | -1.0813 | 6.18E-06 | 9.64E-05 | down-regulated gene |
| LOC643031 | 12704.97 | 6005.789 | -1.08097 | 6.06E-06 | 9.56E-05 | down-regulated gene |
| C4orf34 | 2163.127 | 1022.617 | -1.08085 | 5.08E-06 | 8.47E-05 | down-regulated gene |
| UGDH | 1228.598 | 580.9193 | -1.0806 | 2.23E-05 | 0.000242 | down-regulated gene |
| LOC100129742 | 3542.073 | 1675.76 | -1.07978 | 0.002654 | 0.010744 | down-regulated gene |
| MARVELD2 | 357.8193 | 169.306 | -1.0796 | 6.06E-07 | 2.47E-05 | down-regulated gene |
| CATSPER2 | 1154.342 | 546.2252 | -1.0795 | 4.01E-06 | 7.31E-05 | down-regulated gene |
| SLC22A23 | 514.2096 | 243.4291 | -1.07885 | 1.14E-06 | 3.37E-05 | down-regulated gene |
| MGC12965 | 206.5265 | 97.81323 | -1.07823 | 1.29E-06 | 3.61E-05 | down-regulated gene |
| C12orf23 | 246.8035 | 116.9759 | -1.07715 | 4.70E-06 | 8.07E-05 | down-regulated gene |
| ANKRD30B | 3276.181 | 1554.036 | -1.07599 | 3.51E-05 | 0.000346 | down-regulated gene |
| NME5 | 142.1937 | 67.49417 | -1.07502 | 0.000129 | 0.000949 | down-regulated gene |
| FAHD2B | 669.5764 | 317.8237 | -1.07502 | 4.89E-07 | 2.29E-05 | down-regulated gene |
| GUCA1C | 129.7027 | 61.56748 | -1.07497 | 0.002075 | 0.008813 | down-regulated gene |
| NBPF8 | 511.3735 | 242.75 | -1.07491 | 2.40E-05 | 0.000257 | down-regulated gene |
| SCNN1D | 292.1951 | 138.7501 | -1.07444 | 0.012606 | 0.036858 | down-regulated gene |
| LOC646786 | 989.7303 | 469.9813 | -1.07443 | 1.49E-05 | 0.000179 | down-regulated gene |
| LOC653158 | 578.2251 | 274.8367 | -1.07306 | 3.58E-05 | 0.000349 | down-regulated gene |
| SLC4A5 | 373.7756 | 177.6619 | -1.07304 | 1.87E-06 | 4.44E-05 | down-regulated gene |
| RNASE1 | 751.6351 | 357.2797 | -1.07298 | 0.018129 | 0.049137 | down-regulated gene |
| FMO5 | 376.4978 | 179.0145 | -1.07257 | 0.001213 | 0.005727 | down-regulated gene |
| SOAT2 | 150.0626 | 71.36657 | -1.07224 | 0.009055 | 0.028414 | down-regulated gene |
| UAP1 | 862.2296 | 410.4963 | -1.0707 | 2.00E-05 | 0.000223 | down-regulated gene |
| DEXI | 658.9167 | 313.7799 | -1.07034 | 3.71E-05 | 0.000359 | down-regulated gene |
| HIGD1A | 2210.031 | 1052.974 | -1.0696 | 2.80E-06 | 5.75E-05 | down-regulated gene |
| PRKAB2 | 337.6089 | 161.0115 | -1.06819 | 2.32E-05 | 0.000249 | down-regulated gene |
| ASAP3 | 576.2866 | 274.9767 | -1.06748 | 0.010205 | 0.031254 | down-regulated gene |
| LARP4 | 392.1165 | 187.2405 | -1.06639 | 9.44E-06 | 0.000129 | down-regulated gene |
| ASPA | 138.3711 | 66.09862 | -1.06585 | 3.86E-06 | 7.11E-05 | down-regulated gene |
| MYO10 | 878.6068 | 420.2612 | -1.06393 | 0.000113 | 0.000857 | down-regulated gene |
| SEMA3B | 333.7465 | 159.6899 | -1.06348 | 1.49E-05 | 0.000179 | down-regulated gene |
| LOC646996 | 2012.859 | 963.445 | -1.06297 | 5.72E-06 | 9.12E-05 | down-regulated gene |
| C7 | 137.6693 | 65.96776 | -1.06137 | 2.99E-05 | 0.000304 | down-regulated gene |
| PRKD1 | 164.9331 | 79.0381 | -1.06126 | 4.30E-07 | 2.14E-05 | down-regulated gene |
| C4orf19 | 351.101 | 168.2907 | -1.06093 | 2.11E-06 | 4.79E-05 | down-regulated gene |
| CMBL | 2056.744 | 987.1063 | -1.05908 | 0.00248 | 0.010185 | down-regulated gene |
| LRRC31 | 425.1132 | 204.213 | -1.05777 | 6.08E-05 | 0.000529 | down-regulated gene |
| MGAM | 134.8487 | 64.81108 | -1.05703 | 0.000129 | 0.000949 | down-regulated gene |
| ZNF682 | 1054.088 | 506.7685 | -1.0566 | 6.06E-06 | 9.56E-05 | down-regulated gene |
| UNC5CL | 330.4666 | 158.915 | -1.05625 | 0.000138 | 0.000997 | down-regulated gene |
| GNG7 | 200.5101 | 96.43559 | -1.05604 | 2.80E-06 | 5.75E-05 | down-regulated gene |
| LOC100134053 | 311.4042 | 149.8555 | -1.05522 | 2.03E-06 | 4.67E-05 | down-regulated gene |
| ENTPD5 | 229.8168 | 110.601 | -1.05512 | 0.000125 | 0.000927 | down-regulated gene |
| LOC100134108 | 526.3561 | 253.4524 | -1.05432 | 6.32E-07 | 2.52E-05 | down-regulated gene |
| LOC642333 | 1430.119 | 689.3153 | -1.0529 | 3.10E-06 | 6.17E-05 | down-regulated gene |
| LOC644310 | 272.5291 | 131.4121 | -1.05231 | 7.22E-06 | 0.000108 | down-regulated gene |
| KLF4 | 598.8057 | 288.8295 | -1.05187 | 0.000453 | 0.002581 | down-regulated gene |
| BRI3P1 | 1019.975 | 492.072 | -1.05159 | 3.84E-05 | 0.000369 | down-regulated gene |
| PIP5K2B | 2448.107 | 1182.414 | -1.04993 | 2.49E-05 | 0.000263 | down-regulated gene |
| RAB8B | 514.5542 | 248.5296 | -1.04991 | 8.02E-05 | 0.000653 | down-regulated gene |
| TMEM37 | 209.6249 | 101.2805 | -1.04945 | 0.002316 | 0.009634 | down-regulated gene |
| SLC25A20 | 265.0511 | 128.067 | -1.04937 | 4.89E-07 | 2.29E-05 | down-regulated gene |
| LOC285016 | 135.2917 | 65.511 | -1.04626 | 0.001179 | 0.005597 | down-regulated gene |
| SLC31A1 | 793.986 | 384.9543 | -1.04443 | 0.000292 | 0.001809 | down-regulated gene |
| TRIM2 | 1162.046 | 564.0248 | -1.04284 | 0.000439 | 0.002517 | down-regulated gene |
| KIAA0427 | 299.3187 | 145.4145 | -1.04151 | 5.29E-05 | 0.000478 | down-regulated gene |
| FBXL13 | 141.5915 | 68.86741 | -1.03984 | 0.003378 | 0.012995 | down-regulated gene |
| NKX2-3 | 132.3084 | 64.35522 | -1.03978 | 4.80E-08 | 1.84E-05 | down-regulated gene |
| LHFPL2 | 449.5404 | 218.7284 | -1.03931 | 2.04E-05 | 0.000227 | down-regulated gene |
| LOC728903 | 827.87 | 403.1242 | -1.03818 | 2.53E-06 | 5.43E-05 | down-regulated gene |
| GP2 | 952.528 | 463.9828 | -1.03769 | 0.014807 | 0.041811 | down-regulated gene |
| ACCS | 545.4807 | 265.7377 | -1.03753 | 9.52E-05 | 0.000747 | down-regulated gene |
| GSTTP2 | 191.0924 | 93.25608 | -1.035 | 1.26E-06 | 3.58E-05 | down-regulated gene |
| LOC145837 | 188.5387 | 92.06398 | -1.03415 | 6.30E-05 | 0.000543 | down-regulated gene |
| FMO4 | 216.7968 | 105.9281 | -1.03326 | 1.46E-06 | 3.87E-05 | down-regulated gene |
| GOT1 | 1967.596 | 961.6598 | -1.03284 | 1.89E-05 | 0.000215 | down-regulated gene |
| LOC100128510 | 608.5381 | 297.5117 | -1.0324 | 1.52E-06 | 3.96E-05 | down-regulated gene |
| EPB41L4B | 202.8508 | 99.49614 | -1.02771 | 2.33E-07 | 1.91E-05 | down-regulated gene |
| CHPT1 | 986.6361 | 484.157 | -1.02704 | 4.93E-05 | 0.000449 | down-regulated gene |
| STOX2 | 377.8808 | 185.4598 | -1.02682 | 2.23E-05 | 0.000242 | down-regulated gene |
| SYAP1 | 736.7244 | 362.1923 | -1.02437 | 2.78E-07 | 1.97E-05 | down-regulated gene |
| NR3C2 | 254.422 | 125.1131 | -1.02399 | 1.95E-06 | 4.56E-05 | down-regulated gene |
| AMPD1 | 127.0803 | 62.49649 | -1.02389 | 2.20E-06 | 4.91E-05 | down-regulated gene |
| LOC100133298 | 340.5315 | 167.4772 | -1.02382 | 2.39E-06 | 5.18E-05 | down-regulated gene |
| MFAP4 | 826.8868 | 406.9002 | -1.02301 | 0.002514 | 0.010278 | down-regulated gene |
| KCNMB1 | 254.3705 | 125.2073 | -1.02261 | 2.83E-05 | 0.000293 | down-regulated gene |
| ATP5A1 | 7288.861 | 3588.548 | -1.02229 | 2.75E-06 | 5.70E-05 | down-regulated gene |
| LOC441282 | 135.9647 | 66.95985 | -1.02186 | 6.30E-05 | 0.000543 | down-regulated gene |
| ZBTB8A | 230.2203 | 113.4647 | -1.02077 | 3.36E-06 | 6.48E-05 | down-regulated gene |
| FAM177B | 193.9592 | 95.59884 | -1.02069 | 7.88E-05 | 0.000648 | down-regulated gene |
| CHGB | 209.5448 | 103.2855 | -1.02062 | 0.000467 | 0.002644 | down-regulated gene |
| LEPR | 154.5538 | 76.22197 | -1.01983 | 6.06E-07 | 2.47E-05 | down-regulated gene |
| FLJ44054 | 301.2679 | 148.6818 | -1.01882 | 1.96E-07 | 1.84E-05 | down-regulated gene |
| MTE | 737.9961 | 364.3183 | -1.01841 | 0.001401 | 0.006406 | down-regulated gene |
| ACADS | 320.3648 | 158.221 | -1.01777 | 0.000512 | 0.002841 | down-regulated gene |
| AKR1C2 | 405.3724 | 200.2302 | -1.01759 | 0.00099 | 0.004844 | down-regulated gene |
| LOC389787 | 4759.022 | 2351.165 | -1.01729 | 0.00269 | 0.01088 | down-regulated gene |
| SNX4 | 601.3353 | 297.1262 | -1.01709 | 1.60E-05 | 0.000189 | down-regulated gene |
| CRY2 | 536.0505 | 265.116 | -1.01575 | 5.51E-08 | 1.84E-05 | down-regulated gene |
| RNF213 | 255.3923 | 126.4849 | -1.01375 | 8.86E-07 | 2.98E-05 | down-regulated gene |
| ABCD3 | 365.3025 | 180.9357 | -1.01361 | 2.91E-07 | 1.98E-05 | down-regulated gene |
| LOC100127975 | 659.5236 | 326.6947 | -1.01348 | 1.40E-06 | 3.79E-05 | down-regulated gene |
| PIB5PA | 224.0169 | 111.0336 | -1.01261 | 0.001285 | 0.006 | down-regulated gene |
| SLC25A42 | 259.4177 | 128.6702 | -1.0116 | 3.86E-07 | 2.11E-05 | down-regulated gene |
| PLD1 | 568.2351 | 281.9724 | -1.01093 | 0.000907 | 0.004522 | down-regulated gene |
| NUBPL | 1583.484 | 786.5364 | -1.00952 | 1.49E-05 | 0.000179 | down-regulated gene |
| PBLD | 161.2813 | 80.13705 | -1.00904 | 4.89E-06 | 8.25E-05 | down-regulated gene |
| DLD | 579.2815 | 287.9716 | -1.00834 | 0.000205 | 0.001363 | down-regulated gene |
| PTGER4 | 674.4318 | 335.2797 | -1.00831 | 0.000174 | 0.001196 | down-regulated gene |
| GALNT6 | 334.1657 | 166.1744 | -1.00787 | 0.000364 | 0.002173 | down-regulated gene |
| LOC100131096 | 302.0561 | 150.2963 | -1.00701 | 5.29E-06 | 8.66E-05 | down-regulated gene |
| YIPF5 | 184.7373 | 92.01555 | -1.00553 | 1.96E-07 | 1.84E-05 | down-regulated gene |
| NDUFAB1 | 3513.96 | 1751.802 | -1.00426 | 0.000174 | 0.001196 | down-regulated gene |
| C21orf55 | 11175.01 | 5574.058 | -1.00348 | 4.52E-06 | 7.87E-05 | down-regulated gene |
| PTGS1 | 220.3935 | 110.0176 | -1.00235 | 1.01E-06 | 3.18E-05 | down-regulated gene |
| DLC1 | 245.9133 | 122.7855 | -1.00201 | 3.98E-05 | 0.000379 | down-regulated gene |
| ELOVL6 | 653.772 | 326.4316 | -1.00201 | 0.006587 | 0.022064 | down-regulated gene |
| C1orf175 | 171.2909 | 85.57093 | -1.00126 | 1.72E-06 | 4.24E-05 | down-regulated gene |
| SMPDL3A | 405.6787 | 202.7071 | -1.00094 | 0.002193 | 0.009212 | down-regulated gene |
| TMEM200A | 314.7015 | 157.296 | -1.0005 | 0.002956 | 0.011718 | down-regulated gene |
| KIAA1128 | 295.3608 | 147.6506 | -1.00029 | 6.32E-07 | 2.52E-05 | down-regulated gene |
| RPL4 | 1939.819 | 3882.195 | 1.00095 | 4.89E-07 | 2.29E-05 | up-regulated gene |
| ELF3 | 769.524 | 1540.322 | 1.001193 | 1.86E-05 | 0.000211 | up-regulated gene |
| GPAA1 | 184.1045 | 368.647 | 1.001715 | 6.08E-05 | 0.000529 | up-regulated gene |
| CKAP5 | 292.6199 | 585.9446 | 1.001736 | 7.81E-07 | 2.82E-05 | up-regulated gene |
| TRAF2 | 90.83756 | 181.9822 | 1.002436 | 7.81E-07 | 2.82E-05 | up-regulated gene |
| C1orf112 | 121.5267 | 243.5257 | 1.002801 | 8.68E-07 | 2.97E-05 | up-regulated gene |
| CHAF1B | 85.89877 | 172.3043 | 1.004249 | 7.49E-07 | 2.77E-05 | up-regulated gene |
| EOMES | 71.20699 | 142.869 | 1.004602 | 0.000805 | 0.004111 | up-regulated gene |
| GARS | 894.8 | 1795.839 | 1.005021 | 3.46E-07 | 2.04E-05 | up-regulated gene |
| HIST2H2AC | 314.409 | 631.0155 | 1.005033 | 0.000412 | 0.002392 | up-regulated gene |
| ENO1 | 3824.302 | 7677.36 | 1.005414 | 1.56E-07 | 1.84E-05 | up-regulated gene |
| LOC284821 | 1525.686 | 3063.037 | 1.005505 | 3.58E-05 | 0.000349 | up-regulated gene |
| LOC286444 | 1534.049 | 3081.009 | 1.006058 | 2.64E-06 | 5.58E-05 | up-regulated gene |
| ELK1 | 230.4918 | 463.1195 | 1.006669 | 7.96E-08 | 1.84E-05 | up-regulated gene |
| TOMM40 | 854.8431 | 1718.583 | 1.007488 | 8.58E-06 | 0.000122 | up-regulated gene |
| TOMM6 | 1039.616 | 2091.106 | 1.008216 | 8.68E-07 | 2.97E-05 | up-regulated gene |
| C19orf22 | 653.2568 | 1314.227 | 1.008493 | 1.30E-05 | 0.000164 | up-regulated gene |
| CYB5B | 1414.445 | 2845.847 | 1.008622 | 1.12E-06 | 3.37E-05 | up-regulated gene |
| C6orf173 | 168.8743 | 339.9713 | 1.009464 | 0.001991 | 0.008536 | up-regulated gene |
| ERGIC3 | 1099.163 | 2213.457 | 1.009897 | 4.30E-07 | 2.14E-05 | up-regulated gene |
| KRT18P13 | 414.6707 | 835.0994 | 1.009982 | 0.000129 | 0.000949 | up-regulated gene |
| FOXM1 | 98.64896 | 198.6786 | 1.010061 | 1.14E-06 | 3.37E-05 | up-regulated gene |
| SF3B2 | 434.9181 | 876.1517 | 1.010437 | 2.66E-07 | 1.95E-05 | up-regulated gene |
| NUP37 | 360.3865 | 726.4332 | 1.011285 | 8.50E-07 | 2.92E-05 | up-regulated gene |
| DUS1L | 141.0125 | 284.4882 | 1.012546 | 2.48E-06 | 5.33E-05 | up-regulated gene |
| PRICKLE4 | 854.7082 | 1724.666 | 1.012813 | 6.59E-07 | 2.57E-05 | up-regulated gene |
| ST6GAL1 | 117.5984 | 237.642 | 1.014921 | 0.00092 | 0.004583 | up-regulated gene |
| THOC3 | 90.9288 | 183.8656 | 1.015842 | 2.91E-07 | 1.98E-05 | up-regulated gene |
| LEF1 | 72.66888 | 147.0632 | 1.017027 | 5.33E-07 | 2.36E-05 | up-regulated gene |
| EPHB1 | 58.82644 | 119.0594 | 1.017145 | 1.79E-05 | 0.000206 | up-regulated gene |
| LOC100130562 | 259.2448 | 524.6915 | 1.017154 | 2.08E-05 | 0.000229 | up-regulated gene |
| LOC440928 | 87.59669 | 177.338 | 1.017553 | 5.72E-06 | 9.12E-05 | up-regulated gene |
| MKI67IP | 290.5752 | 588.4147 | 1.017922 | 1.79E-06 | 4.32E-05 | up-regulated gene |
| RPS2 | 1959.498 | 3968.082 | 1.017958 | 1.46E-05 | 0.000177 | up-regulated gene |
| RPS15 | 1623.648 | 3289.65 | 1.018695 | 1.05E-06 | 3.26E-05 | up-regulated gene |
| PACSIN3 | 150.202 | 304.3763 | 1.018951 | 0.001081 | 0.005208 | up-regulated gene |
| COL6A1 | 255.6275 | 518.5191 | 1.020354 | 0.004622 | 0.016704 | up-regulated gene |
| YWHAZ | 1538.312 | 3122.65 | 1.021423 | 8.14E-07 | 2.88E-05 | up-regulated gene |
| RFC4 | 178.522 | 362.4012 | 1.021486 | 6.32E-07 | 2.52E-05 | up-regulated gene |
| CCDC34 | 242.1326 | 491.6024 | 1.021695 | 7.22E-06 | 0.000108 | up-regulated gene |
| FKBP1A | 291.1456 | 591.2058 | 1.02192 | 2.72E-07 | 1.97E-05 | up-regulated gene |
| FGFR4 | 108.52 | 220.4831 | 1.022707 | 0.00018 | 0.001226 | up-regulated gene |
| DNTTIP1 | 288.9197 | 587.1058 | 1.022952 | 1.14E-07 | 1.84E-05 | up-regulated gene |
| TFDP1 | 406.4617 | 826.1489 | 1.023282 | 1.83E-06 | 4.41E-05 | up-regulated gene |
| MGC61598 | 296.1835 | 602.1701 | 1.02368 | 6.08E-05 | 0.000529 | up-regulated gene |
| LOC283392 | 99.64947 | 202.6953 | 1.024378 | 0.000342 | 0.002065 | up-regulated gene |
| OGFR | 172.095 | 350.0913 | 1.024526 | 3.71E-05 | 0.000359 | up-regulated gene |
| GRWD1 | 223.6628 | 455.4704 | 1.026032 | 4.12E-07 | 2.13E-05 | up-regulated gene |
| RSL1D1 | 445.3544 | 907.0032 | 1.026154 | 3.46E-07 | 2.04E-05 | up-regulated gene |
| TTPAL | 130.9444 | 266.713 | 1.026333 | 1.04E-07 | 1.84E-05 | up-regulated gene |
| IL1A | 59.09911 | 120.376 | 1.026339 | 0.000596 | 0.003227 | up-regulated gene |
| POLD1 | 93.97984 | 191.547 | 1.027275 | 3.94E-07 | 2.11E-05 | up-regulated gene |
| ATL3 | 294.8908 | 601.3504 | 1.028025 | 7.36E-06 | 0.00011 | up-regulated gene |
| LMNB1 | 99.54404 | 203.0276 | 1.028269 | 8.50E-07 | 2.92E-05 | up-regulated gene |
| TFAP2A | 76.60351 | 156.2941 | 1.02878 | 8.42E-06 | 0.00012 | up-regulated gene |
| HNRNPA3 | 129.9742 | 265.1921 | 1.028813 | 3.10E-07 | 1.98E-05 | up-regulated gene |
| CD44 | 500.5807 | 1021.364 | 1.028822 | 8.74E-05 | 0.0007 | up-regulated gene |
| RAD54L | 100.2816 | 204.6287 | 1.028951 | 5.10E-07 | 2.30E-05 | up-regulated gene |
| SNHG1 | 266.0853 | 543.2231 | 1.029656 | 7.22E-06 | 0.000108 | up-regulated gene |
| PLXNA1 | 115.8028 | 236.49 | 1.030109 | 9.81E-06 | 0.000133 | up-regulated gene |
| ALDH3B2 | 93.82344 | 191.623 | 1.030251 | 0.017339 | 0.0474 | up-regulated gene |
| MSN | 360.1706 | 736.1229 | 1.031266 | 0.000163 | 0.001136 | up-regulated gene |
| RAE1 | 128.0282 | 261.7213 | 1.03157 | 4.37E-08 | 1.84E-05 | up-regulated gene |
| PRPF4 | 274.1332 | 560.7508 | 1.032483 | 6.33E-08 | 1.84E-05 | up-regulated gene |
| MIF | 1788.055 | 3658.138 | 1.032718 | 2.20E-06 | 4.91E-05 | up-regulated gene |
| BCL2L1 | 649.5538 | 1329.093 | 1.032921 | 1.93E-05 | 0.000217 | up-regulated gene |
| HIST2H2AA4 | 342.3158 | 701.7566 | 1.035643 | 0.001441 | 0.006554 | up-regulated gene |
| LMNA | 662.4522 | 1358.51 | 1.036137 | 5.80E-07 | 2.43E-05 | up-regulated gene |
| LOC642477 | 73.39606 | 150.5229 | 1.036208 | 4.01E-06 | 7.31E-05 | up-regulated gene |
| RNPS1 | 247.2615 | 507.8268 | 1.038299 | 3.04E-07 | 1.98E-05 | up-regulated gene |
| DHCR7 | 262.6741 | 539.6779 | 1.038824 | 2.20E-06 | 4.91E-05 | up-regulated gene |
| NUP210 | 92.56917 | 190.2587 | 1.039358 | 0.000364 | 0.002173 | up-regulated gene |
| BCL3 | 290.7719 | 597.9279 | 1.040084 | 0.000222 | 0.001459 | up-regulated gene |
| NFKB2 | 70.02007 | 143.9987 | 1.040215 | 5.51E-08 | 1.84E-05 | up-regulated gene |
| TMEM118 | 115.6332 | 237.9182 | 1.04091 | 7.49E-05 | 0.00062 | up-regulated gene |
| EFNB1 | 191.6934 | 394.6352 | 1.041719 | 0.000412 | 0.002392 | up-regulated gene |
| SF3B3 | 219.7304 | 452.6815 | 1.042762 | 1.12E-06 | 3.37E-05 | up-regulated gene |
| PABPC1L | 217.35 | 447.8585 | 1.043023 | 0.007454 | 0.024283 | up-regulated gene |
| LSR | 132.6427 | 273.5559 | 1.04429 | 3.84E-05 | 0.000369 | up-regulated gene |
| C11orf82 | 81.7787 | 168.7227 | 1.044857 | 1.96E-07 | 1.84E-05 | up-regulated gene |
| ULBP2 | 69.0113 | 142.558 | 1.046645 | 6.30E-05 | 0.000543 | up-regulated gene |
| PLA2G7 | 68.75144 | 142.1137 | 1.047584 | 1.04E-07 | 1.84E-05 | up-regulated gene |
| IRF8 | 311.2949 | 643.5161 | 1.047694 | 0.000673 | 0.003562 | up-regulated gene |
| IFFO2 | 74.08837 | 153.1618 | 1.047737 | 2.14E-07 | 1.84E-05 | up-regulated gene |
| PDCD5 | 346.2146 | 716.2713 | 1.048839 | 4.30E-07 | 2.14E-05 | up-regulated gene |
| CLIC1 | 1906.633 | 3945.476 | 1.049172 | 1.00E-06 | 3.18E-05 | up-regulated gene |
| STK4 | 644.4887 | 1333.868 | 1.049389 | 1.05E-06 | 3.26E-05 | up-regulated gene |
| GLIPR1 | 64.1984 | 132.9753 | 1.050549 | 2.03E-06 | 4.67E-05 | up-regulated gene |
| NUP205 | 349.9631 | 725.0123 | 1.050803 | 6.93E-08 | 1.84E-05 | up-regulated gene |
| C19orf48 | 181.2395 | 375.7435 | 1.05185 | 4.18E-06 | 7.48E-05 | up-regulated gene |
| GAD1 | 66.92408 | 138.8115 | 1.052529 | 6.06E-07 | 2.47E-05 | up-regulated gene |
| LOC647954 | 508.4863 | 1055.068 | 1.053056 | 0.000704 | 0.003705 | up-regulated gene |
| LAGE3 | 364.4831 | 756.626 | 1.053728 | 1.19E-05 | 0.000153 | up-regulated gene |
| NHP2 | 292.1165 | 606.4671 | 1.053885 | 9.09E-06 | 0.000126 | up-regulated gene |
| SF3B4 | 597.375 | 1240.883 | 1.054659 | 1.64E-07 | 1.84E-05 | up-regulated gene |
| PIWIL1 | 62.9265 | 130.8061 | 1.055691 | 0.014146 | 0.040375 | up-regulated gene |
| HRASLS3 | 522.6221 | 1086.442 | 1.055771 | 0.008022 | 0.025728 | up-regulated gene |
| SBNO2 | 108.4704 | 225.6151 | 1.056562 | 4.03E-07 | 2.13E-05 | up-regulated gene |
| CSDA | 1140.909 | 2375.808 | 1.058235 | 8.92E-06 | 0.000125 | up-regulated gene |
| LOC389049 | 68.24264 | 142.1404 | 1.058571 | 4.80E-08 | 1.84E-05 | up-regulated gene |
| UBAP2L | 306.136 | 637.6518 | 1.058596 | 1.66E-05 | 0.000194 | up-regulated gene |
| FAM64A | 75.33851 | 157.0639 | 1.059892 | 2.14E-07 | 1.84E-05 | up-regulated gene |
| CDC123 | 207.1941 | 432.0878 | 1.060342 | 1.43E-07 | 1.84E-05 | up-regulated gene |
| CTPS | 162.2717 | 338.5012 | 1.060749 | 4.68E-07 | 2.23E-05 | up-regulated gene |
| SLC7A1 | 570.2525 | 1189.97 | 1.061252 | 4.01E-06 | 7.31E-05 | up-regulated gene |
| FLJ12684 | 59.81229 | 124.8714 | 1.06193 | 7.80E-06 | 0.000114 | up-regulated gene |
| NFE2 | 55.13259 | 115.1073 | 1.062002 | 3.84E-05 | 0.000369 | up-regulated gene |
| C20orf24 | 1890.69 | 3948.25 | 1.0623 | 1.62E-06 | 4.14E-05 | up-regulated gene |
| TH1L | 433.2982 | 905.2959 | 1.063029 | 2.04E-07 | 1.84E-05 | up-regulated gene |
| LOC646942 | 1110.625 | 2321.31 | 1.063568 | 1.57E-07 | 1.84E-05 | up-regulated gene |
| EBNA1BP2 | 666.4676 | 1393.07 | 1.063662 | 3.17E-07 | 1.98E-05 | up-regulated gene |
| DUSP1 | 549.8171 | 1149.352 | 1.063797 | 0.013825 | 0.039646 | up-regulated gene |
| KRT15 | 194.5628 | 406.8982 | 1.064432 | 0.005668 | 0.019583 | up-regulated gene |
| PLAUR | 128.4647 | 268.7709 | 1.065005 | 6.99E-05 | 0.000587 | up-regulated gene |
| PLEKHO2 | 76.05852 | 159.1391 | 1.065106 | 1.71E-07 | 1.84E-05 | up-regulated gene |
| LOC401537 | 630.1622 | 1318.758 | 1.065385 | 6.06E-06 | 9.56E-05 | up-regulated gene |
| CBR3 | 95.37201 | 199.6248 | 1.065653 | 0.0004 | 0.002336 | up-regulated gene |
| RN5S9 | 233.331 | 488.6963 | 1.06656 | 0.006267 | 0.021232 | up-regulated gene |
| KCNJ2 | 74.4172 | 156.0977 | 1.068741 | 2.59E-06 | 5.48E-05 | up-regulated gene |
| CSTB | 2532.952 | 5313.49 | 1.06884 | 0.000653 | 0.003473 | up-regulated gene |
| CCL2 | 98.72316 | 207.3365 | 1.070514 | 0.000673 | 0.003562 | up-regulated gene |
| SNHG5 | 1132.098 | 2378.945 | 1.071324 | 0.000412 | 0.002392 | up-regulated gene |
| NUP107 | 244.6441 | 514.712 | 1.073081 | 2.91E-07 | 1.98E-05 | up-regulated gene |
| ASPM | 244.6419 | 514.8193 | 1.073395 | 3.71E-05 | 0.000359 | up-regulated gene |
| C17orf53 | 120.8109 | 254.4648 | 1.074715 | 5.80E-07 | 2.43E-05 | up-regulated gene |
| HOXC9 | 61.16177 | 128.8524 | 1.075018 | 4.68E-07 | 2.23E-05 | up-regulated gene |
| FAM60A | 218.5701 | 460.8275 | 1.076131 | 1.87E-06 | 4.44E-05 | up-regulated gene |
| ILDR1 | 139.5834 | 294.4039 | 1.076669 | 1.19E-05 | 0.000153 | up-regulated gene |
| HLA-E | 1563.494 | 3299.511 | 1.077479 | 2.00E-05 | 0.000223 | up-regulated gene |
| LOC730246 | 2057.694 | 4344.398 | 1.078128 | 8.71E-08 | 1.84E-05 | up-regulated gene |
| OAS2 | 171.9177 | 362.9999 | 1.078251 | 0.002654 | 0.010744 | up-regulated gene |
| LOC392871 | 96.2054 | 203.4682 | 1.080614 | 0.000237 | 0.001537 | up-regulated gene |
| TUBB | 565.6635 | 1196.639 | 1.080972 | 8.50E-07 | 2.92E-05 | up-regulated gene |
| AGRN | 185.0063 | 391.6682 | 1.082058 | 0.000439 | 0.002517 | up-regulated gene |
| IGFBP7 | 674.2229 | 1427.736 | 1.082432 | 0.005121 | 0.018096 | up-regulated gene |
| RPL8 | 1859.68 | 3940.065 | 1.083165 | 1.40E-07 | 1.84E-05 | up-regulated gene |
| SDC4 | 629.1488 | 1333.898 | 1.084175 | 0.002316 | 0.009634 | up-regulated gene |
| DNAJC9 | 346.2194 | 734.6539 | 1.085378 | 4.30E-07 | 2.14E-05 | up-regulated gene |
| AQP9 | 54.10656 | 114.8123 | 1.085402 | 3.17E-07 | 1.98E-05 | up-regulated gene |
| ECGF1 | 935.7517 | 1987.425 | 1.086703 | 0.002316 | 0.009634 | up-regulated gene |
| F12 | 208.597 | 443.1255 | 1.086997 | 0.000387 | 0.002282 | up-regulated gene |
| CDCA2 | 73.3824 | 155.9117 | 1.087224 | 3.17E-07 | 1.98E-05 | up-regulated gene |
| NUTF2 | 185.3246 | 393.9975 | 1.088132 | 1.87E-07 | 1.84E-05 | up-regulated gene |
| CCNF | 233.6688 | 496.98 | 1.088723 | 1.79E-06 | 4.32E-05 | up-regulated gene |
| PRSS22 | 72.67652 | 154.699 | 1.089903 | 3.86E-06 | 7.11E-05 | up-regulated gene |
| ANXA2 | 1852.559 | 3943.834 | 1.090079 | 1.19E-05 | 0.000153 | up-regulated gene |
| OSM | 72.12481 | 153.5876 | 1.090494 | 3.58E-05 | 0.000349 | up-regulated gene |
| MAPRE1 | 714.1269 | 1520.968 | 1.090737 | 1.96E-07 | 1.84E-05 | up-regulated gene |
| HOXA9 | 64.66919 | 137.8143 | 1.091576 | 4.13E-05 | 0.00039 | up-regulated gene |
| AGTRAP | 86.05388 | 183.4752 | 1.092273 | 1.50E-07 | 1.84E-05 | up-regulated gene |
| MYOF | 253.0488 | 539.615 | 1.092515 | 0.000467 | 0.002644 | up-regulated gene |
| LOC653879 | 74.36463 | 158.7102 | 1.093706 | 1.38E-05 | 0.00017 | up-regulated gene |
| ANXA1 | 1740.856 | 3719.926 | 1.095477 | 0.008839 | 0.027907 | up-regulated gene |
| DDX18 | 482.8479 | 1032.667 | 1.096735 | 1.14E-07 | 1.84E-05 | up-regulated gene |
| LOC100129553 | 3158.823 | 6756.813 | 1.096956 | 5.08E-06 | 8.47E-05 | up-regulated gene |
| BOP1 | 593.775 | 1270.431 | 1.097329 | 4.52E-06 | 7.87E-05 | up-regulated gene |
| SLC1A3 | 86.87368 | 185.88 | 1.097381 | 0.000102 | 0.000788 | up-regulated gene |
| NT5C3L | 158.0663 | 338.2884 | 1.097724 | 0.000961 | 0.004731 | up-regulated gene |
| TMSB10 | 5538.648 | 11856.02 | 1.098015 | 2.77E-07 | 1.97E-05 | up-regulated gene |
| RNASEH2A | 106.4619 | 228.2718 | 1.100415 | 1.43E-07 | 1.84E-05 | up-regulated gene |
| ZNF696 | 100.0352 | 214.562 | 1.100887 | 1.40E-07 | 1.84E-05 | up-regulated gene |
| ATIC | 930.9087 | 1996.943 | 1.101082 | 1.37E-07 | 1.84E-05 | up-regulated gene |
| SLC20A1 | 342.2428 | 734.1963 | 1.101146 | 1.07E-06 | 3.31E-05 | up-regulated gene |
| NOTCH3 | 191.6596 | 411.307 | 1.101669 | 3.98E-05 | 0.000379 | up-regulated gene |
| LOC440737 | 1281.502 | 2750.456 | 1.101835 | 3.17E-07 | 1.98E-05 | up-regulated gene |
| GTF3C5 | 166.5953 | 357.6607 | 1.102244 | 4.52E-06 | 7.87E-05 | up-regulated gene |
| SERPINE1 | 75.70658 | 162.5372 | 1.102279 | 4.12E-07 | 2.13E-05 | up-regulated gene |
| SUPT16H | 569.7777 | 1223.83 | 1.102932 | 1.31E-07 | 1.84E-05 | up-regulated gene |
| THOC4 | 105.7782 | 227.2114 | 1.102992 | 8.86E-07 | 2.98E-05 | up-regulated gene |
| DDX21 | 286.5151 | 615.7379 | 1.103705 | 4.34E-06 | 7.68E-05 | up-regulated gene |
| PLK4 | 101.6501 | 218.5385 | 1.104276 | 1.37E-07 | 1.84E-05 | up-regulated gene |
| TKT | 1279.668 | 2753.821 | 1.105665 | 3.29E-06 | 6.38E-05 | up-regulated gene |
| ADAMTS9 | 68.15813 | 146.8113 | 1.107006 | 1.59E-06 | 4.06E-05 | up-regulated gene |
| TRIP6 | 421.4652 | 908.3658 | 1.10786 | 0.000736 | 0.003833 | up-regulated gene |
| PPAT | 117.7679 | 253.9275 | 1.108471 | 2.23E-07 | 1.87E-05 | up-regulated gene |
| C4BPA | 62.16328 | 134.0602 | 1.108747 | 0.00099 | 0.004844 | up-regulated gene |
| ARPC1B | 157.4944 | 340.0205 | 1.110322 | 4.43E-05 | 0.000413 | up-regulated gene |
| VAT1 | 339.0457 | 732.1809 | 1.11072 | 0.000561 | 0.003068 | up-regulated gene |
| S100A10 | 4430.691 | 9570.313 | 1.111034 | 6.51E-05 | 0.000556 | up-regulated gene |
| PPIAL4A | 3451.734 | 7463.678 | 1.112566 | 1.64E-07 | 1.84E-05 | up-regulated gene |
| MFHAS1 | 118.3555 | 255.9368 | 1.11266 | 3.94E-07 | 2.11E-05 | up-regulated gene |
| C6orf48 | 321.6953 | 696.2088 | 1.113825 | 5.94E-06 | 9.39E-05 | up-regulated gene |
| LOC441775 | 2675.096 | 5793.952 | 1.114957 | 1.46E-06 | 3.87E-05 | up-regulated gene |
| TEX10 | 118.2907 | 256.205 | 1.114961 | 9.99E-08 | 1.84E-05 | up-regulated gene |
| PPIL1 | 152.4757 | 330.4689 | 1.115935 | 1.01E-06 | 3.18E-05 | up-regulated gene |
| RPL36 | 1118.309 | 2424.348 | 1.116278 | 5.72E-06 | 9.12E-05 | up-regulated gene |
| RALY | 460.1284 | 997.7407 | 1.116628 | 3.56E-06 | 6.71E-05 | up-regulated gene |
| LSM7 | 487.8923 | 1058.24 | 1.117033 | 7.18E-07 | 2.70E-05 | up-regulated gene |
| ALS2CR4 | 109.9186 | 238.7947 | 1.119335 | 2.29E-06 | 5.02E-05 | up-regulated gene |
| SHMT2 | 752.1042 | 1636.628 | 1.121722 | 1.14E-06 | 3.37E-05 | up-regulated gene |
| BRIX1 | 260.572 | 567.7226 | 1.123504 | 5.39E-06 | 8.81E-05 | up-regulated gene |
| PLEKHB1 | 177.6603 | 387.1734 | 1.123858 | 0.013825 | 0.039646 | up-regulated gene |
| MFGE8 | 758.5737 | 1653.519 | 1.124179 | 0.000121 | 0.000904 | up-regulated gene |
| PTDSS1 | 475.7038 | 1036.951 | 1.124212 | 2.78E-07 | 1.97E-05 | up-regulated gene |
| NUSAP1 | 211.4885 | 461.2253 | 1.124893 | 7.36E-06 | 0.00011 | up-regulated gene |
| NUDT5 | 322.7533 | 703.8823 | 1.124902 | 5.33E-07 | 2.36E-05 | up-regulated gene |
| COL5A1 | 346.2806 | 755.6409 | 1.125759 | 0.002316 | 0.009634 | up-regulated gene |
| TCF3 | 120.8192 | 263.7602 | 1.126377 | 2.91E-07 | 1.98E-05 | up-regulated gene |
| MRPS17 | 348.613 | 763.0238 | 1.130102 | 1.04E-07 | 1.84E-05 | up-regulated gene |
| NAT10 | 366.7925 | 803.089 | 1.130596 | 2.23E-07 | 1.87E-05 | up-regulated gene |
| RSRC1 | 80.79668 | 176.9449 | 1.130932 | 5.80E-07 | 2.43E-05 | up-regulated gene |
| TRIM29 | 79.39087 | 173.9302 | 1.131463 | 0.000412 | 0.002392 | up-regulated gene |
| DPY19L1 | 208.6808 | 457.1977 | 1.13152 | 9.64E-07 | 3.10E-05 | up-regulated gene |
| HSD3B7 | 107.8021 | 236.2442 | 1.131894 | 3.61E-07 | 2.08E-05 | up-regulated gene |
| SAA4 | 62.7531 | 137.664 | 1.133392 | 2.69E-06 | 5.60E-05 | up-regulated gene |
| LYAR | 144.2717 | 316.5155 | 1.133488 | 9.12E-08 | 1.84E-05 | up-regulated gene |
| LOC642489 | 570.4698 | 1252.487 | 1.134573 | 3.61E-07 | 2.08E-05 | up-regulated gene |
| DAP3 | 468.9086 | 1030.479 | 1.135936 | 2.04E-07 | 1.84E-05 | up-regulated gene |
| SNRPD2 | 1723.702 | 3788.804 | 1.136233 | 1.83E-07 | 1.84E-05 | up-regulated gene |
| HMGA1 | 156.1479 | 343.3045 | 1.136576 | 4.30E-07 | 2.14E-05 | up-regulated gene |
| S100A16 | 712.9555 | 1567.821 | 1.136877 | 0.001361 | 0.006276 | up-regulated gene |
| HLA-B | 1922.876 | 4232.771 | 1.138336 | 0.000445 | 0.002553 | up-regulated gene |
| LOC100130003 | 1493.024 | 3288.699 | 1.139279 | 1.04E-07 | 1.84E-05 | up-regulated gene |
| LAS1L | 170.5305 | 376.0271 | 1.140807 | 1.24E-06 | 3.53E-05 | up-regulated gene |
| CENPA | 76.04077 | 167.727 | 1.14127 | 2.14E-07 | 1.84E-05 | up-regulated gene |
| ILF2 | 642.2413 | 1417.287 | 1.141944 | 7.98E-07 | 2.87E-05 | up-regulated gene |
| COCH | 66.99832 | 147.9154 | 1.142575 | 6.94E-06 | 0.000105 | up-regulated gene |
| RPP25 | 116.1728 | 256.708 | 1.143856 | 4.79E-07 | 2.27E-05 | up-regulated gene |
| MGC87042 | 194.0985 | 429.0132 | 1.144233 | 0.006753 | 0.022476 | up-regulated gene |
| HSPC111 | 157.0765 | 347.5842 | 1.145895 | 2.78E-07 | 1.97E-05 | up-regulated gene |
| RACGAP1 | 108.5304 | 240.3359 | 1.146953 | 2.91E-07 | 1.98E-05 | up-regulated gene |
| TP63 | 91.24281 | 202.0576 | 1.146984 | 0.002583 | 0.010511 | up-regulated gene |
| C13orf37 | 107.16 | 237.3321 | 1.147141 | 3.04E-07 | 1.98E-05 | up-regulated gene |
| CHEK1 | 76.38199 | 169.2321 | 1.147699 | 1.37E-07 | 1.84E-05 | up-regulated gene |
| MRTO4 | 111.2841 | 246.777 | 1.148961 | 4.80E-08 | 1.84E-05 | up-regulated gene |
| GLT25D1 | 454.0285 | 1008.061 | 1.150728 | 2.38E-06 | 5.18E-05 | up-regulated gene |
| C9orf16 | 77.39101 | 171.8789 | 1.151155 | 8.33E-08 | 1.84E-05 | up-regulated gene |
| SNRPB2 | 468.9203 | 1041.713 | 1.151543 | 1.04E-07 | 1.84E-05 | up-regulated gene |
| GTF3A | 1260.678 | 2800.683 | 1.151579 | 6.59E-07 | 2.57E-05 | up-regulated gene |
| BATF | 71.02392 | 157.8251 | 1.151949 | 3.29E-06 | 6.38E-05 | up-regulated gene |
| C1S | 150.8282 | 335.169 | 1.151983 | 0.000805 | 0.004111 | up-regulated gene |
| CSF3R | 68.95287 | 153.304 | 1.152712 | 1.10E-05 | 0.000145 | up-regulated gene |
| MTHFD2 | 139.8569 | 311.1059 | 1.153454 | 2.91E-07 | 1.98E-05 | up-regulated gene |
| GCNT3 | 1674.999 | 3726.401 | 1.153623 | 0.012901 | 0.037549 | up-regulated gene |
| SLC39A1 | 461.2122 | 1026.97 | 1.154891 | 4.11E-07 | 2.13E-05 | up-regulated gene |
| BIK | 103.6386 | 230.9558 | 1.156056 | 0.000133 | 0.000972 | up-regulated gene |
| TMEM185B | 134.0638 | 298.7736 | 1.156133 | 4.37E-08 | 1.84E-05 | up-regulated gene |
| PRIC285 | 413.4979 | 921.8234 | 1.15661 | 2.58E-05 | 0.000271 | up-regulated gene |
| HDGF | 302.9435 | 675.6879 | 1.157308 | 4.89E-07 | 2.29E-05 | up-regulated gene |
| NIP7 | 250.7463 | 559.2674 | 1.15731 | 5.51E-08 | 1.84E-05 | up-regulated gene |
| CYP2W1 | 56.61499 | 126.4912 | 1.159781 | 0.000105 | 0.000812 | up-regulated gene |
| LOC389168 | 1561.136 | 3489.934 | 1.160604 | 6.18E-08 | 1.84E-05 | up-regulated gene |
| HNRNPL | 326.2783 | 729.6337 | 1.161069 | 7.60E-08 | 1.84E-05 | up-regulated gene |
| LOC100130561 | 195.8541 | 438.1261 | 1.161567 | 5.80E-07 | 2.43E-05 | up-regulated gene |
| LOC388707 | 347.3412 | 777.3191 | 1.162153 | 3.29E-06 | 6.38E-05 | up-regulated gene |
| EIF1 | 1380.015 | 3090.358 | 1.16309 | 1.57E-05 | 0.000187 | up-regulated gene |
| NUDT3 | 221.5931 | 496.2909 | 1.163273 | 1.50E-07 | 1.84E-05 | up-regulated gene |
| DDIT4 | 600.3735 | 1346.509 | 1.165292 | 0.000387 | 0.002282 | up-regulated gene |
| SULT2B1 | 78.4886 | 176.0604 | 1.165516 | 0.002075 | 0.008813 | up-regulated gene |
| NUP93 | 197.1951 | 442.4438 | 1.16587 | 1.43E-07 | 1.84E-05 | up-regulated gene |
| TOMM34 | 635.1118 | 1425.039 | 1.165919 | 1.99E-06 | 4.65E-05 | up-regulated gene |
| AEBP1 | 288.9883 | 650.0695 | 1.169583 | 0.015147 | 0.042531 | up-regulated gene |
| TRIM28 | 213.6537 | 480.84 | 1.170283 | 1.87E-06 | 4.44E-05 | up-regulated gene |
| WBSCR22 | 456.415 | 1027.591 | 1.170848 | 1.64E-07 | 1.84E-05 | up-regulated gene |
| PTPN1 | 294.1987 | 662.7074 | 1.171581 | 7.95E-08 | 1.84E-05 | up-regulated gene |
| IL17RB | 108.4185 | 244.5403 | 1.173461 | 4.34E-06 | 7.68E-05 | up-regulated gene |
| PSMB3 | 1494.908 | 3375.337 | 1.174974 | 9.98E-08 | 1.84E-05 | up-regulated gene |
| HCP5 | 717.4462 | 1620.071 | 1.175115 | 0.0001 | 0.000779 | up-regulated gene |
| BARD1 | 142.3778 | 321.6042 | 1.175562 | 5.80E-07 | 2.43E-05 | up-regulated gene |
| GPR172A | 523.4454 | 1182.465 | 1.175686 | 3.94E-07 | 2.11E-05 | up-regulated gene |
| LOC100127888 | 66.92335 | 151.2703 | 1.176548 | 1.79E-05 | 0.000206 | up-regulated gene |
| CALU | 250.3652 | 566.0555 | 1.17691 | 9.24E-07 | 3.05E-05 | up-regulated gene |
| PUS7 | 226.6371 | 512.9514 | 1.178438 | 3.04E-07 | 1.98E-05 | up-regulated gene |
| C9orf140 | 117.5637 | 266.094 | 1.178493 | 1.46E-06 | 3.87E-05 | up-regulated gene |
| VASN | 157.6178 | 356.9095 | 1.179128 | 0.000105 | 0.000812 | up-regulated gene |
| EIF4A3 | 801.4634 | 1814.994 | 1.179257 | 5.44E-07 | 2.40E-05 | up-regulated gene |
| RPS3 | 3295.595 | 7476.372 | 1.181799 | 2.38E-07 | 1.93E-05 | up-regulated gene |
| TBCB | 401.6 | 911.2199 | 1.18204 | 1.65E-06 | 4.16E-05 | up-regulated gene |
| NME1-NME2 | 633.4554 | 1439.449 | 1.184201 | 2.75E-06 | 5.70E-05 | up-regulated gene |
| FAM83H | 513.1511 | 1166.656 | 1.184924 | 2.11E-06 | 4.79E-05 | up-regulated gene |
| HEATR1 | 192.0833 | 436.9798 | 1.185834 | 8.71E-08 | 1.84E-05 | up-regulated gene |
| SOX9 | 320.3063 | 729.1199 | 1.186704 | 5.50E-06 | 8.89E-05 | up-regulated gene |
| RPP40 | 315.2641 | 718.9755 | 1.189381 | 1.35E-06 | 3.69E-05 | up-regulated gene |
| SNRPG | 2232.39 | 5097.275 | 1.191137 | 7.95E-08 | 1.84E-05 | up-regulated gene |
| LAMA5 | 995.1441 | 2279.239 | 1.195575 | 7.23E-05 | 0.000604 | up-regulated gene |
| VSNL1 | 82.51994 | 189.0459 | 1.195922 | 0.004866 | 0.017376 | up-regulated gene |
| S100A8 | 1582.42 | 3632.225 | 1.198721 | 1.21E-05 | 0.000156 | up-regulated gene |
| DSCC1 | 115.1295 | 264.4262 | 1.199607 | 5.02E-08 | 1.84E-05 | up-regulated gene |
| C10orf2 | 79.0631 | 181.744 | 1.200831 | 7.60E-08 | 1.84E-05 | up-regulated gene |
| IGFBP3 | 452.8793 | 1041.649 | 1.20167 | 0.008946 | 0.028229 | up-regulated gene |
| CCT3 | 401.1364 | 922.7879 | 1.201906 | 5.77E-08 | 1.84E-05 | up-regulated gene |
| MAD2L1 | 95.4701 | 220.0706 | 1.204846 | 1.52E-06 | 3.96E-05 | up-regulated gene |
| RPL39L | 104.4958 | 240.8781 | 1.204859 | 0.000282 | 0.001767 | up-regulated gene |
| CKLF | 266.4186 | 614.3777 | 1.205431 | 5.08E-06 | 8.47E-05 | up-regulated gene |
| ZMYND19 | 158.1628 | 364.8067 | 1.205722 | 1.57E-07 | 1.84E-05 | up-regulated gene |
| LGALS1 | 680.8784 | 1571.968 | 1.207103 | 0.000489 | 0.002744 | up-regulated gene |
| RRP12 | 127.5054 | 294.8318 | 1.209334 | 1.50E-07 | 1.84E-05 | up-regulated gene |
| ATAD2 | 94.18783 | 217.9748 | 1.210549 | 1.71E-07 | 1.84E-05 | up-regulated gene |
| GRINA | 191.9164 | 445.185 | 1.213927 | 3.86E-06 | 7.11E-05 | up-regulated gene |
| HOXA13 | 61.57033 | 142.8595 | 1.214289 | 1.40E-06 | 3.79E-05 | up-regulated gene |
| KIF4A | 63.59554 | 147.634 | 1.215027 | 1.04E-07 | 1.84E-05 | up-regulated gene |
| SH3KBP1 | 237.4463 | 551.7365 | 1.216378 | 1.35E-06 | 3.69E-05 | up-regulated gene |
| TTF2 | 157.0706 | 365.5405 | 1.218618 | 1.71E-07 | 1.84E-05 | up-regulated gene |
| ADORA2B | 82.53277 | 192.1041 | 1.21885 | 1.49E-05 | 0.000179 | up-regulated gene |
| ANLN | 105.0717 | 244.6459 | 1.219322 | 8.30E-05 | 0.000672 | up-regulated gene |
| TOP1MT | 96.56092 | 224.961 | 1.220164 | 1.57E-07 | 1.84E-05 | up-regulated gene |
| SNHG3-RCC1 | 113.197 | 264.2161 | 1.222883 | 8.71E-08 | 1.84E-05 | up-regulated gene |
| ADCY3 | 406.5704 | 949.1329 | 1.223105 | 4.49E-07 | 2.19E-05 | up-regulated gene |
| TMEM166 | 61.44391 | 143.5398 | 1.224109 | 4.89E-06 | 8.25E-05 | up-regulated gene |
| C2orf82 | 145.352 | 339.7219 | 1.224803 | 0.003956 | 0.014759 | up-regulated gene |
| TYMP | 88.97006 | 207.9935 | 1.225147 | 1.43E-05 | 0.000174 | up-regulated gene |
| OLR1 | 73.03006 | 170.8607 | 1.226259 | 0.000715 | 0.003738 | up-regulated gene |
| CNOT3 | 75.89922 | 177.6162 | 1.226606 | 8.15E-07 | 2.88E-05 | up-regulated gene |
| RPL30 | 3512.116 | 8221.12 | 1.226994 | 3.03E-07 | 1.98E-05 | up-regulated gene |
| PSMD3 | 106.76 | 250.1886 | 1.228645 | 3.46E-07 | 2.04E-05 | up-regulated gene |
| FADS1 | 70.27239 | 164.7999 | 1.229686 | 3.10E-05 | 0.000313 | up-regulated gene |
| ETS2 | 334.4072 | 784.282 | 1.229767 | 0.000528 | 0.002918 | up-regulated gene |
| IER5 | 120.1625 | 282.1037 | 1.231238 | 8.32E-07 | 2.92E-05 | up-regulated gene |
| MESP1 | 72.96953 | 171.3933 | 1.231944 | 5.48E-05 | 0.00049 | up-regulated gene |
| LTB | 120.9452 | 284.106 | 1.232076 | 0.000439 | 0.002517 | up-regulated gene |
| LOC100133565 | 70.67792 | 166.0654 | 1.23242 | 0.014146 | 0.040375 | up-regulated gene |
| MCM3 | 594.5353 | 1396.971 | 1.232468 | 7.49E-07 | 2.77E-05 | up-regulated gene |
| PBK | 125.5054 | 295.1764 | 1.233828 | 8.02E-05 | 0.000653 | up-regulated gene |
| HSPA6 | 78.31899 | 184.3218 | 1.234793 | 1.43E-05 | 0.000174 | up-regulated gene |
| CXCL13 | 79.55809 | 187.4136 | 1.236145 | 0.002878 | 0.011472 | up-regulated gene |
| CDK4 | 432.3135 | 1018.822 | 1.236752 | 2.04E-07 | 1.84E-05 | up-regulated gene |
| RAD51AP1 | 126.382 | 298.1991 | 1.238485 | 7.18E-07 | 2.70E-05 | up-regulated gene |
| PAICS | 343.2586 | 810.3237 | 1.239202 | 1.79E-07 | 1.84E-05 | up-regulated gene |
| S100A2 | 129.552 | 305.9324 | 1.239682 | 0.00088 | 0.004416 | up-regulated gene |
| OIP5 | 117.5095 | 277.6161 | 1.240313 | 2.66E-07 | 1.95E-05 | up-regulated gene |
| PRR7 | 62.48086 | 147.6931 | 1.241116 | 6.94E-08 | 1.84E-05 | up-regulated gene |
| SNRPB | 997.3456 | 2360.246 | 1.242772 | 1.09E-07 | 1.84E-05 | up-regulated gene |
| MCM5 | 159.3442 | 377.3663 | 1.243819 | 2.33E-07 | 1.91E-05 | up-regulated gene |
| BAT2 | 134.5157 | 318.5696 | 1.243834 | 6.88E-07 | 2.65E-05 | up-regulated gene |
| CCT2 | 443.4107 | 1051.066 | 1.245138 | 7.03E-07 | 2.70E-05 | up-regulated gene |
| PROCR | 144.9301 | 343.7412 | 1.245965 | 1.23E-05 | 0.000157 | up-regulated gene |
| HOXC8 | 61.43453 | 145.7168 | 1.246045 | 2.04E-07 | 1.84E-05 | up-regulated gene |
| GGH | 174.9797 | 415.8889 | 1.249011 | 0.000174 | 0.001196 | up-regulated gene |
| PPAN | 81.21463 | 193.381 | 1.251635 | 9.99E-08 | 1.84E-05 | up-regulated gene |
| SLC4A11 | 123.456 | 294.13 | 1.252456 | 0.000653 | 0.003473 | up-regulated gene |
| XPO5 | 148.7544 | 354.5246 | 1.252954 | 4.58E-08 | 1.84E-05 | up-regulated gene |
| CMTM7 | 195.2028 | 465.2583 | 1.253058 | 0.000109 | 0.000834 | up-regulated gene |
| GTPBP4 | 601.4358 | 1433.948 | 1.253511 | 2.78E-07 | 1.97E-05 | up-regulated gene |
| FZD2 | 65.14948 | 155.6906 | 1.256856 | 1.14E-07 | 1.84E-05 | up-regulated gene |
| PTTG1 | 317.1711 | 757.9978 | 1.256932 | 4.89E-06 | 8.25E-05 | up-regulated gene |
| CDT1 | 117.6199 | 281.393 | 1.258454 | 4.18E-06 | 7.48E-05 | up-regulated gene |
| LOC730316 | 654.0918 | 1565.62 | 1.259169 | 1.07E-06 | 3.31E-05 | up-regulated gene |
| NFE2L3 | 144.0205 | 344.8127 | 1.259539 | 1.79E-05 | 0.000206 | up-regulated gene |
| CCNA2 | 141.2796 | 338.4249 | 1.260283 | 4.89E-07 | 2.29E-05 | up-regulated gene |
| FOXJ1 | 64.90206 | 155.6144 | 1.261639 | 0.00018 | 0.001226 | up-regulated gene |
| NUDT1 | 160.8526 | 386.1175 | 1.263301 | 1.37E-07 | 1.84E-05 | up-regulated gene |
| FER1L3 | 208.0645 | 499.845 | 1.26445 | 8.02E-05 | 0.000653 | up-regulated gene |
| MX2 | 212.1081 | 509.9114 | 1.265447 | 0.007829 | 0.025255 | up-regulated gene |
| CXCL2 | 98.70435 | 237.5799 | 1.267227 | 0.000693 | 0.003652 | up-regulated gene |
| POM121C | 624.5143 | 1503.577 | 1.267592 | 1.24E-06 | 3.53E-05 | up-regulated gene |
| POLR2D | 98.98886 | 238.7836 | 1.270366 | 4.37E-08 | 1.84E-05 | up-regulated gene |
| MCM7 | 324.6744 | 783.6916 | 1.271292 | 1.87E-07 | 1.84E-05 | up-regulated gene |
| HOXA10 | 60.15034 | 145.2568 | 1.271961 | 2.04E-07 | 1.84E-05 | up-regulated gene |
| LOC650369 | 956.8513 | 2310.973 | 1.272134 | 1.04E-07 | 1.84E-05 | up-regulated gene |
| GPSM2 | 119.857 | 289.8827 | 1.274155 | 1.14E-06 | 3.37E-05 | up-regulated gene |
| PKM2 | 299.1919 | 723.8833 | 1.274686 | 1.29E-06 | 3.61E-05 | up-regulated gene |
| STIP1 | 448.7842 | 1085.916 | 1.274819 | 1.31E-07 | 1.84E-05 | up-regulated gene |
| NCAPD2 | 155.901 | 377.5051 | 1.275865 | 7.60E-08 | 1.84E-05 | up-regulated gene |
| RBM38 | 111.0802 | 268.9863 | 1.275931 | 5.26E-08 | 1.84E-05 | up-regulated gene |
| ITGB4BP | 1109.077 | 2686.147 | 1.276179 | 5.10E-07 | 2.30E-05 | up-regulated gene |
| BHLHB2 | 791.0607 | 1918.184 | 1.277881 | 6.75E-05 | 0.00057 | up-regulated gene |
| FAM83A | 73.95579 | 179.4611 | 1.278936 | 0.00083 | 0.004211 | up-regulated gene |
| TUBB2C | 918.9449 | 2231.959 | 1.28026 | 7.18E-07 | 2.70E-05 | up-regulated gene |
| PLEK | 86.77757 | 210.8347 | 1.280718 | 3.98E-05 | 0.000379 | up-regulated gene |
| S100A3 | 63.43835 | 154.172 | 1.281113 | 1.01E-06 | 3.18E-05 | up-regulated gene |
| IL20RB | 101.8199 | 247.5509 | 1.281706 | 0.000596 | 0.003227 | up-regulated gene |
| PTMS | 180.6042 | 439.2086 | 1.282075 | 3.21E-05 | 0.000321 | up-regulated gene |
| THOC6 | 77.35311 | 188.257 | 1.283172 | 4.37E-08 | 1.84E-05 | up-regulated gene |
| RAB34 | 124.6102 | 303.7236 | 1.285337 | 0.002381 | 0.009843 | up-regulated gene |
| BIRC5 | 94.2268 | 230.541 | 1.290814 | 2.78E-07 | 1.97E-05 | up-regulated gene |
| LOC646766 | 2073.809 | 5077.688 | 1.291889 | 1.64E-07 | 1.84E-05 | up-regulated gene |
| PODXL2 | 156.8748 | 384.3802 | 1.292921 | 0.002654 | 0.010744 | up-regulated gene |
| RPL29 | 122.2705 | 299.676 | 1.293327 | 4.18E-06 | 7.48E-05 | up-regulated gene |
| FSCN1 | 486.3366 | 1192.666 | 1.294162 | 0.000793 | 0.004077 | up-regulated gene |
| POLR1C | 288.2832 | 707.0577 | 1.294341 | 1.37E-07 | 1.84E-05 | up-regulated gene |
| LCE3D | 59.91021 | 147.129 | 1.296207 | 0.010956 | 0.033039 | up-regulated gene |
| SRM | 167.628 | 411.845 | 1.296838 | 4.49E-07 | 2.19E-05 | up-regulated gene |
| UCK2 | 119.389 | 293.4241 | 1.297317 | 4.58E-08 | 1.84E-05 | up-regulated gene |
| RAN | 611.8445 | 1504.508 | 1.298055 | 2.33E-07 | 1.91E-05 | up-regulated gene |
| LOC646723 | 1997.199 | 4912.612 | 1.298512 | 1.30E-05 | 0.000164 | up-regulated gene |
| PRMT1 | 238.8213 | 588.3582 | 1.300763 | 2.14E-07 | 1.84E-05 | up-regulated gene |
| CENPF | 138.5352 | 341.3463 | 1.300983 | 2.04E-07 | 1.84E-05 | up-regulated gene |
| COL3A1 | 807.4547 | 1989.847 | 1.301204 | 0.006112 | 0.0208 | up-regulated gene |
| LOC388796 | 173.4586 | 429.0019 | 1.306392 | 1.87E-06 | 4.44E-05 | up-regulated gene |
| GLDC | 58.90446 | 146.1806 | 1.311303 | 0.000842 | 0.004271 | up-regulated gene |
| PRRX2 | 134.6498 | 334.182 | 1.311422 | 0.001857 | 0.008048 | up-regulated gene |
| PPP1R15A | 467.7937 | 1161.587 | 1.312152 | 4.09E-06 | 7.43E-05 | up-regulated gene |
| NCAPG | 151.3227 | 375.7666 | 1.312208 | 3.16E-06 | 6.24E-05 | up-regulated gene |
| CCNE1 | 78.22284 | 194.2544 | 1.312285 | 6.33E-08 | 1.84E-05 | up-regulated gene |
| PSMB2 | 554.1906 | 1376.524 | 1.312576 | 7.26E-08 | 1.84E-05 | up-regulated gene |
| IMPDH2 | 765.8608 | 1904.44 | 1.314213 | 2.59E-06 | 5.48E-05 | up-regulated gene |
| HEYL | 97.52657 | 242.8069 | 1.315942 | 2.66E-07 | 1.95E-05 | up-regulated gene |
| PDPN | 77.99507 | 194.5517 | 1.318699 | 1.25E-07 | 1.84E-05 | up-regulated gene |
| GAL | 56.44423 | 140.8213 | 1.318968 | 0.001963 | 0.008424 | up-regulated gene |
| BCL2L12 | 139.8679 | 348.9745 | 1.319056 | 7.60E-08 | 1.84E-05 | up-regulated gene |
| FLJ22662 | 700.5194 | 1748.548 | 1.31966 | 5.48E-05 | 0.00049 | up-regulated gene |
| KIF2C | 92.41703 | 230.9821 | 1.32155 | 6.62E-08 | 1.84E-05 | up-regulated gene |
| PA2G4 | 472.2324 | 1180.275 | 1.321555 | 2.49E-07 | 1.95E-05 | up-regulated gene |
| WARS | 477.6503 | 1196.374 | 1.324642 | 2.08E-05 | 0.000229 | up-regulated gene |
| C16orf75 | 161.7073 | 405.3731 | 1.325865 | 1.52E-06 | 3.96E-05 | up-regulated gene |
| NINJ2 | 88.09652 | 220.9183 | 1.326356 | 1.09E-06 | 3.34E-05 | up-regulated gene |
| TNNC1 | 66.15024 | 165.9141 | 1.326618 | 7.22E-06 | 0.000108 | up-regulated gene |
| LOC341457 | 3214.085 | 8072.794 | 1.32866 | 2.04E-07 | 1.84E-05 | up-regulated gene |
| C7orf59 | 537.8835 | 1351.207 | 1.328883 | 3.94E-07 | 2.11E-05 | up-regulated gene |
| CSE1L | 406.0523 | 1020.328 | 1.329296 | 5.77E-08 | 1.84E-05 | up-regulated gene |
| LOC649553 | 315.1667 | 794.076 | 1.333162 | 1.53E-07 | 1.84E-05 | up-regulated gene |
| LOC91461 | 110.4096 | 278.4996 | 1.334809 | 0.000163 | 0.001136 | up-regulated gene |
| KIF20A | 104.5333 | 264.414 | 1.338835 | 4.30E-07 | 2.14E-05 | up-regulated gene |
| FXYD5 | 491.487 | 1244.836 | 1.340731 | 6.75E-05 | 0.00057 | up-regulated gene |
| TIMELESS | 173.1193 | 438.63 | 1.341237 | 9.54E-08 | 1.84E-05 | up-regulated gene |
| FERMT1 | 191.1213 | 484.8726 | 1.343117 | 2.69E-06 | 5.60E-05 | up-regulated gene |
| PDCD2L | 127.7408 | 324.7974 | 1.346321 | 3.04E-07 | 1.98E-05 | up-regulated gene |
| NCOA7 | 540.982 | 1381.966 | 1.353069 | 6.18E-06 | 9.64E-05 | up-regulated gene |
| HLA-DQA1 | 128.169 | 327.7029 | 1.354341 | 0.0107 | 0.032437 | up-regulated gene |
| DDAH2 | 150.8759 | 386.169 | 1.35587 | 2.69E-06 | 5.60E-05 | up-regulated gene |
| EPHA2 | 92.17668 | 236.0865 | 1.356842 | 3.31E-07 | 2.01E-05 | up-regulated gene |
| C1orf135 | 63.45487 | 162.6031 | 1.357552 | 5.51E-08 | 1.84E-05 | up-regulated gene |
| LOC731314 | 215.6615 | 552.6752 | 1.357664 | 5.80E-07 | 2.43E-05 | up-regulated gene |
| LAIR2 | 80.24202 | 205.6448 | 1.357725 | 6.75E-05 | 0.00057 | up-regulated gene |
| PYGB | 561.9848 | 1441.726 | 1.359194 | 0.000353 | 0.002117 | up-regulated gene |
| PMEPA1 | 113.7321 | 291.9033 | 1.359851 | 4.18E-06 | 7.48E-05 | up-regulated gene |
| AHCY | 748.1738 | 1920.471 | 1.360015 | 2.84E-07 | 1.98E-05 | up-regulated gene |
| LOC651524 | 113.1392 | 290.5496 | 1.360686 | 9.84E-05 | 0.000767 | up-regulated gene |
| MXRA5 | 586.9392 | 1508.59 | 1.361918 | 0.001483 | 0.006704 | up-regulated gene |
| IER5L | 71.41721 | 184.093 | 1.366091 | 9.54E-08 | 1.84E-05 | up-regulated gene |
| CTHRC1 | 114.8528 | 296.6582 | 1.369016 | 0.000321 | 0.001957 | up-regulated gene |
| HSPH1 | 602.605 | 1558.46 | 1.370837 | 1.91E-06 | 4.52E-05 | up-regulated gene |
| LOC441246 | 2828.561 | 7330.786 | 1.3739 | 1.14E-07 | 1.84E-05 | up-regulated gene |
| CLDN3 | 488.4685 | 1266.404 | 1.374401 | 0.001285 | 0.006 | up-regulated gene |
| JPH1 | 71.2638 | 184.771 | 1.374497 | 8.15E-07 | 2.88E-05 | up-regulated gene |
| CTGF | 332.931 | 863.3854 | 1.374781 | 5.48E-05 | 0.00049 | up-regulated gene |
| PSAT1 | 122.6053 | 318.6153 | 1.377795 | 5.48E-05 | 0.00049 | up-regulated gene |
| NOLC1 | 73.94022 | 193.2612 | 1.386121 | 5.02E-08 | 1.84E-05 | up-regulated gene |
| NPM3 | 231.2425 | 605.5178 | 1.388763 | 1.01E-06 | 3.18E-05 | up-regulated gene |
| RRAGD | 112.9459 | 295.7657 | 1.388823 | 0.000596 | 0.003227 | up-regulated gene |
| ADRM1 | 377.0926 | 992.6979 | 1.396436 | 4.80E-08 | 1.84E-05 | up-regulated gene |
| KRT7 | 203.6626 | 536.9391 | 1.398578 | 0.001483 | 0.006704 | up-regulated gene |
| ZC3H12A | 167.2485 | 441.2989 | 1.399763 | 2.88E-05 | 0.000296 | up-regulated gene |
| DKC1 | 567.0959 | 1497.249 | 1.400649 | 6.93E-08 | 1.84E-05 | up-regulated gene |
| TNFAIP2 | 71.77821 | 189.7318 | 1.402343 | 6.06E-07 | 2.47E-05 | up-regulated gene |
| CKS2 | 326.4302 | 865.3825 | 1.406563 | 1.28E-05 | 0.000162 | up-regulated gene |
| HSPA1B | 1075.477 | 2851.188 | 1.406586 | 7.79E-06 | 0.000114 | up-regulated gene |
| FSTL1 | 140.0693 | 371.4029 | 1.406845 | 1.33E-05 | 0.000165 | up-regulated gene |
| PRSS3 | 487.6685 | 1296.649 | 1.410815 | 0.000364 | 0.002173 | up-regulated gene |
| CCL3L3 | 69.99807 | 186.1243 | 1.410879 | 1.72E-06 | 4.24E-05 | up-regulated gene |
| TK1 | 181.1669 | 482.2664 | 1.412511 | 4.52E-06 | 7.87E-05 | up-regulated gene |
| SLC38A1 | 119.4477 | 318.711 | 1.41587 | 1.59E-06 | 4.06E-05 | up-regulated gene |
| TYMS | 185.1837 | 494.1385 | 1.415958 | 2.20E-06 | 4.91E-05 | up-regulated gene |
| SQLE | 220.9481 | 589.6803 | 1.416226 | 1.43E-05 | 0.000174 | up-regulated gene |
| EPHB2 | 76.64178 | 204.9373 | 1.41898 | 4.70E-06 | 8.07E-05 | up-regulated gene |
| C10orf10 | 170.2913 | 456.0605 | 1.421221 | 0.000129 | 0.000949 | up-regulated gene |
| PLEKHG4 | 90.28618 | 241.8467 | 1.421516 | 8.15E-07 | 2.88E-05 | up-regulated gene |
| TYROBP | 145.5237 | 390.0686 | 1.422474 | 0.000138 | 0.000997 | up-regulated gene |
| PTK7 | 79.17374 | 212.2639 | 1.422765 | 3.77E-07 | 2.10E-05 | up-regulated gene |
| BUB1 | 80.45974 | 216.0831 | 1.425247 | 4.49E-07 | 2.19E-05 | up-regulated gene |
| UHRF1 | 89.15973 | 240.0079 | 1.428618 | 9.99E-08 | 1.84E-05 | up-regulated gene |
| FAM83D | 105.3568 | 283.868 | 1.429937 | 5.29E-06 | 8.66E-05 | up-regulated gene |
| CDCA8 | 127.707 | 345.1955 | 1.434575 | 1.87E-07 | 1.84E-05 | up-regulated gene |
| LOC440160 | 74.9732 | 203.1168 | 1.437863 | 7.96E-08 | 1.84E-05 | up-regulated gene |
| FEN1 | 209.2955 | 567.374 | 1.438758 | 5.10E-07 | 2.30E-05 | up-regulated gene |
| EIF6 | 402.8288 | 1095.839 | 1.443796 | 2.33E-07 | 1.91E-05 | up-regulated gene |
| S100A9 | 1524.57 | 4152.453 | 1.445561 | 8.10E-06 | 0.000116 | up-regulated gene |
| HJURP | 108.3048 | 295.1944 | 1.446568 | 1.50E-07 | 1.84E-05 | up-regulated gene |
| MARCKSL1 | 653.3789 | 1787.38 | 1.451855 | 2.48E-06 | 5.33E-05 | up-regulated gene |
| FOXD2 | 115.7733 | 317.0744 | 1.453518 | 9.44E-06 | 0.000129 | up-regulated gene |
| LIPG | 76.92211 | 210.9205 | 1.455229 | 1.14E-06 | 3.37E-05 | up-regulated gene |
| LOC100130919 | 729.8017 | 2001.672 | 1.455629 | 6.33E-08 | 1.84E-05 | up-regulated gene |
| C7orf40 | 169.0945 | 464.9987 | 1.459397 | 6.93E-08 | 1.84E-05 | up-regulated gene |
| RENBP | 117.9014 | 324.4672 | 1.460492 | 0.000274 | 0.001724 | up-regulated gene |
| LOC729217 | 151.0646 | 416.1758 | 1.462027 | 1.37E-07 | 1.84E-05 | up-regulated gene |
| GPNMB | 89.30994 | 246.0711 | 1.462182 | 5.87E-05 | 0.000516 | up-regulated gene |
| PTPN20 | 132.7321 | 366.5904 | 1.465651 | 0.000174 | 0.001196 | up-regulated gene |
| SLC1A5 | 173.7097 | 481.7054 | 1.471473 | 5.80E-07 | 2.43E-05 | up-regulated gene |
| PKDCC | 212.4669 | 589.3745 | 1.471946 | 0.000512 | 0.002841 | up-regulated gene |
| ODC1 | 467.9096 | 1298.483 | 1.472525 | 1.46E-06 | 3.87E-05 | up-regulated gene |
| C17orf96 | 82.89948 | 230.302 | 1.474092 | 9.84E-07 | 3.16E-05 | up-regulated gene |
| HTRA1 | 286.6873 | 798.7086 | 1.478191 | 1.38E-05 | 0.00017 | up-regulated gene |
| FSTL3 | 75.02846 | 209.2106 | 1.479446 | 2.44E-07 | 1.93E-05 | up-regulated gene |
| C7orf68 | 122.6429 | 341.9946 | 1.47951 | 1.40E-06 | 3.79E-05 | up-regulated gene |
| CPNE1 | 131.9054 | 367.9353 | 1.479949 | 7.60E-08 | 1.84E-05 | up-regulated gene |
| GNA15 | 144.6553 | 403.69 | 1.480628 | 0.000105 | 0.000812 | up-regulated gene |
| EMILIN2 | 130.705 | 365.0577 | 1.48181 | 2.11E-06 | 4.79E-05 | up-regulated gene |
| LOC345041 | 307.4086 | 859.6059 | 1.483518 | 3.17E-07 | 1.98E-05 | up-regulated gene |
| MTHFD1L | 107.3981 | 300.5704 | 1.484734 | 1.04E-07 | 1.84E-05 | up-regulated gene |
| FAM38A | 361.1524 | 1011.927 | 1.486425 | 2.39E-06 | 5.18E-05 | up-regulated gene |
| C20orf199 | 312.4319 | 877.7251 | 1.490227 | 1.50E-07 | 1.84E-05 | up-regulated gene |
| LAPTM5 | 98.54423 | 276.8669 | 1.490349 | 2.59E-06 | 5.48E-05 | up-regulated gene |
| C2orf89 | 74.43598 | 209.2159 | 1.490921 | 1.86E-05 | 0.000211 | up-regulated gene |
| RCC2 | 702.1118 | 1976.28 | 1.493015 | 6.33E-08 | 1.84E-05 | up-regulated gene |
| KRT80 | 93.21355 | 262.6004 | 1.494258 | 0.000331 | 0.00201 | up-regulated gene |
| OLFML2B | 58.3393 | 164.5337 | 1.495843 | 1.20E-07 | 1.84E-05 | up-regulated gene |
| EXOSC5 | 100.5182 | 284.7076 | 1.502024 | 6.62E-08 | 1.84E-05 | up-regulated gene |
| LOC647000 | 934.219 | 2648.95 | 1.503588 | 1.22E-07 | 1.84E-05 | up-regulated gene |
| VSIG8 | 65.86655 | 187.2028 | 1.506984 | 0.006112 | 0.0208 | up-regulated gene |
| IRAK2 | 127.2649 | 361.8837 | 1.507692 | 9.64E-07 | 3.10E-05 | up-regulated gene |
| MED30 | 132.459 | 376.7091 | 1.507905 | 1.25E-07 | 1.84E-05 | up-regulated gene |
| ASF1B | 123.4938 | 351.7534 | 1.510125 | 6.06E-07 | 2.47E-05 | up-regulated gene |
| LRFN4 | 81.28201 | 231.5881 | 1.510553 | 5.51E-08 | 1.84E-05 | up-regulated gene |
| TNFRSF12A | 166.8812 | 476.0671 | 1.512343 | 1.93E-05 | 0.000217 | up-regulated gene |
| LAMC2 | 101.3222 | 289.1893 | 1.513064 | 2.48E-06 | 5.33E-05 | up-regulated gene |
| AHNAK2 | 143.5773 | 410.6237 | 1.515989 | 0.008839 | 0.027907 | up-regulated gene |
| AGT | 79.22622 | 226.7008 | 1.51674 | 1.35E-06 | 3.69E-05 | up-regulated gene |
| TGM2 | 163.2762 | 467.3432 | 1.517168 | 0.000715 | 0.003738 | up-regulated gene |
| CDKN3 | 107.3015 | 307.9548 | 1.521049 | 2.44E-07 | 1.93E-05 | up-regulated gene |
| CKAP2L | 89.34488 | 256.8732 | 1.523599 | 1.20E-07 | 1.84E-05 | up-regulated gene |
| LOC653061 | 120.8078 | 348.7242 | 1.529373 | 0.000113 | 0.000857 | up-regulated gene |
| GDPD5 | 77.39798 | 223.8074 | 1.53189 | 1.14E-06 | 3.37E-05 | up-regulated gene |
| ARID3A | 154.5224 | 447.2923 | 1.533402 | 0.000596 | 0.003227 | up-regulated gene |
| RIPK2 | 149.0503 | 432.3436 | 1.536379 | 6.04E-08 | 1.84E-05 | up-regulated gene |
| CDCA7 | 277.8418 | 808.5214 | 1.541022 | 1.60E-05 | 0.000189 | up-regulated gene |
| TTYH3 | 161.3674 | 471.0419 | 1.545506 | 3.94E-07 | 2.11E-05 | up-regulated gene |
| TAP1 | 1129.947 | 3299.627 | 1.546048 | 6.68E-06 | 0.000102 | up-regulated gene |
| MELK | 148.4222 | 433.9719 | 1.547895 | 5.00E-07 | 2.30E-05 | up-regulated gene |
| GINS2 | 150.766 | 441.3868 | 1.549732 | 7.81E-07 | 2.82E-05 | up-regulated gene |
| CDC45L | 147.1719 | 431.6542 | 1.552374 | 6.59E-07 | 2.57E-05 | up-regulated gene |
| NME1 | 442.1924 | 1299.987 | 1.555751 | 1.37E-07 | 1.84E-05 | up-regulated gene |
| ITGB2 | 323.204 | 950.2644 | 1.555884 | 0.000113 | 0.000857 | up-regulated gene |
| DUSP14 | 122.7794 | 361.4503 | 1.557729 | 6.32E-07 | 2.52E-05 | up-regulated gene |
| CCDC86 | 135.0713 | 398.031 | 1.559159 | 6.33E-08 | 1.84E-05 | up-regulated gene |
| SHFM1 | 573.8035 | 1692.444 | 1.56048 | 5.26E-08 | 1.84E-05 | up-regulated gene |
| THBS1 | 74.41134 | 220.5864 | 1.56775 | 8.50E-07 | 2.92E-05 | up-regulated gene |
| CENPN | 154.6496 | 458.5692 | 1.568136 | 6.04E-08 | 1.84E-05 | up-regulated gene |
| C1orf59 | 183.5131 | 545.4139 | 1.571468 | 3.04E-06 | 6.06E-05 | up-regulated gene |
| SOD2 | 184.0897 | 547.3902 | 1.57216 | 3.29E-06 | 6.38E-05 | up-regulated gene |
| IFI6 | 1187.236 | 3534.04 | 1.573711 | 5.77E-05 | 0.000511 | up-regulated gene |
| ZFP36 | 425.7472 | 1268.846 | 1.575449 | 5.29E-06 | 8.66E-05 | up-regulated gene |
| CSAG1 | 66.16674 | 197.4008 | 1.57695 | 0.001401 | 0.006406 | up-regulated gene |
| LOC100131139 | 83.36781 | 248.7955 | 1.577398 | 3.04E-06 | 6.06E-05 | up-regulated gene |
| SALL4 | 67.15418 | 200.5041 | 1.578083 | 3.16E-06 | 6.24E-05 | up-regulated gene |
| CEACAM5 | 387.8717 | 1158.443 | 1.578536 | 0.004992 | 0.017727 | up-regulated gene |
| HOXB5 | 219.8846 | 658.4464 | 1.582319 | 1.66E-05 | 0.000194 | up-regulated gene |
| RAB31 | 298.3861 | 895.3231 | 1.585228 | 1.72E-05 | 0.0002 | up-regulated gene |
| STAT1 | 592.0004 | 1782.471 | 1.590209 | 1.72E-06 | 4.24E-05 | up-regulated gene |
| CFB | 1167.425 | 3519.224 | 1.591927 | 0.000152 | 0.00108 | up-regulated gene |
| GZMB | 80.88934 | 244.0258 | 1.593012 | 0.000186 | 0.001259 | up-regulated gene |
| CEBPD | 499.8552 | 1508.611 | 1.593639 | 6.88E-07 | 2.65E-05 | up-regulated gene |
| PTGES | 91.75032 | 277.3617 | 1.595984 | 4.93E-05 | 0.000449 | up-regulated gene |
| ERBB2 | 422.7753 | 1281.175 | 1.599504 | 0.015147 | 0.042531 | up-regulated gene |
| SNRPF | 569.7158 | 1728.276 | 1.601019 | 6.93E-08 | 1.84E-05 | up-regulated gene |
| UBE2T | 144.5726 | 441.5495 | 1.610781 | 1.43E-07 | 1.84E-05 | up-regulated gene |
| GPRC5A | 117.7764 | 362.1644 | 1.620595 | 3.56E-06 | 6.71E-05 | up-regulated gene |
| JUNB | 82.76964 | 254.6731 | 1.621473 | 6.33E-08 | 1.84E-05 | up-regulated gene |
| ASGR1 | 90.39048 | 280.4683 | 1.633595 | 0.000782 | 0.004024 | up-regulated gene |
| LOC100134364 | 389.2045 | 1208.147 | 1.634196 | 0.001857 | 0.008048 | up-regulated gene |
| TGIF2 | 85.2567 | 265.5062 | 1.638861 | 6.93E-08 | 1.84E-05 | up-regulated gene |
| SFN | 510.9252 | 1594.731 | 1.642129 | 0.005813 | 0.019967 | up-regulated gene |
| AURKB | 107.4504 | 335.78 | 1.643845 | 2.55E-07 | 1.95E-05 | up-regulated gene |
| F5 | 87.13234 | 273.3378 | 1.649405 | 0.002317 | 0.009634 | up-regulated gene |
| HOXA11AS | 63.22612 | 199.6125 | 1.65861 | 3.71E-06 | 6.93E-05 | up-regulated gene |
| NXT1 | 273.1702 | 862.9299 | 1.659444 | 4.58E-08 | 1.84E-05 | up-regulated gene |
| FKBP10 | 66.5892 | 211.1306 | 1.664776 | 1.79E-07 | 1.84E-05 | up-regulated gene |
| AURKA | 243.7736 | 776.9643 | 1.672307 | 1.20E-07 | 1.84E-05 | up-regulated gene |
| GRB7 | 97.55398 | 312.0246 | 1.677387 | 1.33E-05 | 0.000165 | up-regulated gene |
| CTSK | 163.3717 | 522.8275 | 1.678177 | 9.44E-06 | 0.000129 | up-regulated gene |
| FCER1G | 78.60379 | 252.3792 | 1.682922 | 1.87E-06 | 4.44E-05 | up-regulated gene |
| CEP55 | 157.6556 | 508.4531 | 1.689338 | 5.56E-07 | 2.41E-05 | up-regulated gene |
| CCNB2 | 203.2249 | 656.6228 | 1.691987 | 2.66E-07 | 1.95E-05 | up-regulated gene |
| OLFM4 | 982.6966 | 3180.876 | 1.694606 | 0.008628 | 0.027383 | up-regulated gene |
| TPX2 | 110.7747 | 359.094 | 1.696733 | 6.33E-08 | 1.84E-05 | up-regulated gene |
| KLK7 | 120.0436 | 389.2548 | 1.697157 | 0.00406 | 0.015071 | up-regulated gene |
| BYSL | 112.8756 | 368.5245 | 1.707027 | 5.02E-08 | 1.84E-05 | up-regulated gene |
| C2 | 86.80466 | 283.4143 | 1.707068 | 5.10E-07 | 2.30E-05 | up-regulated gene |
| EBPL | 201.4917 | 657.9311 | 1.707216 | 1.25E-07 | 1.84E-05 | up-regulated gene |
| SPP1 | 70.00123 | 228.7702 | 1.708447 | 1.14E-06 | 3.37E-05 | up-regulated gene |
| CKS1B | 333.8815 | 1094.506 | 1.712872 | 5.10E-07 | 2.30E-05 | up-regulated gene |
| NT5DC2 | 111.4888 | 368.3605 | 1.724219 | 2.33E-07 | 1.91E-05 | up-regulated gene |
| SLPI | 493.005 | 1629.216 | 1.724503 | 0.002878 | 0.011472 | up-regulated gene |
| COL8A1 | 68.88319 | 228.1453 | 1.727729 | 4.12E-07 | 2.13E-05 | up-regulated gene |
| CD14 | 349.0424 | 1158.252 | 1.730475 | 6.59E-07 | 2.57E-05 | up-regulated gene |
| CYBA | 717.9378 | 2383.782 | 1.731322 | 1.34E-06 | 3.69E-05 | up-regulated gene |
| PLAU | 156.3307 | 519.6833 | 1.733032 | 1.46E-06 | 3.87E-05 | up-regulated gene |
| SULF1 | 142.2033 | 474.4016 | 1.738154 | 0.000615 | 0.00331 | up-regulated gene |
| MCM4 | 150.8512 | 505.016 | 1.743202 | 1.25E-07 | 1.84E-05 | up-regulated gene |
| MYB | 212.7381 | 712.5152 | 1.743842 | 1.60E-05 | 0.000189 | up-regulated gene |
| TUBB3 | 91.5298 | 307.7717 | 1.749547 | 2.00E-05 | 0.000223 | up-regulated gene |
| CPVL | 132.8987 | 447.2446 | 1.750737 | 0.001525 | 0.006858 | up-regulated gene |
| LMNB2 | 227.5663 | 768.8503 | 1.756416 | 1.43E-07 | 1.84E-05 | up-regulated gene |
| PTGS2 | 100.1617 | 338.5875 | 1.757198 | 1.49E-05 | 0.000179 | up-regulated gene |
| ECM1 | 201.0072 | 681.1096 | 1.76064 | 0.000364 | 0.002173 | up-regulated gene |
| HBA1 | 105.763 | 359.7843 | 1.766298 | 0.001401 | 0.006406 | up-regulated gene |
| SAA1 | 67.07072 | 228.8349 | 1.770552 | 7.50E-06 | 0.000111 | up-regulated gene |
| NDUFA4L2 | 69.47779 | 237.0527 | 1.770584 | 8.50E-07 | 2.92E-05 | up-regulated gene |
| JUP | 475.6818 | 1626.169 | 1.773408 | 7.50E-06 | 0.000111 | up-regulated gene |
| CRABP2 | 171.3728 | 588.6393 | 1.780246 | 8.02E-05 | 0.000653 | up-regulated gene |
| ETV4 | 69.76463 | 240.0405 | 1.78271 | 5.77E-08 | 1.84E-05 | up-regulated gene |
| MGC4677 | 166.6628 | 575.0233 | 1.786688 | 6.06E-07 | 2.47E-05 | up-regulated gene |
| HSP90AB1 | 820.3793 | 2837.952 | 1.790487 | 8.71E-08 | 1.84E-05 | up-regulated gene |
| SLC2A3 | 205.3041 | 710.6273 | 1.791331 | 7.65E-06 | 0.000113 | up-regulated gene |
| KRT6A | 1141.195 | 3951.747 | 1.791945 | 0.013798 | 0.039646 | up-regulated gene |
| CDC20 | 481.6418 | 1677.377 | 1.800174 | 5.10E-07 | 2.30E-05 | up-regulated gene |
| BANF1 | 192.0916 | 669.9881 | 1.802341 | 5.02E-08 | 1.84E-05 | up-regulated gene |
| HAVCR2 | 120.6734 | 420.9251 | 1.802456 | 4.12E-07 | 2.13E-05 | up-regulated gene |
| INDO | 65.46354 | 228.3983 | 1.802788 | 0.000117 | 0.000879 | up-regulated gene |
| IFI44L | 95.32497 | 336.0025 | 1.817546 | 0.005388 | 0.018821 | up-regulated gene |
| COL6A3 | 309.2334 | 1090.362 | 1.81804 | 7.22E-06 | 0.000108 | up-regulated gene |
| KIFC1 | 137.0056 | 483.8846 | 1.820429 | 1.50E-07 | 1.84E-05 | up-regulated gene |
| RRS1 | 104.7627 | 370.9008 | 1.823909 | 4.58E-08 | 1.84E-05 | up-regulated gene |
| PRC1 | 270.074 | 956.5317 | 1.824458 | 1.25E-07 | 1.84E-05 | up-regulated gene |
| TGFBI | 478.8679 | 1696.291 | 1.824684 | 2.88E-05 | 0.000296 | up-regulated gene |
| FOS | 417.7619 | 1499.161 | 1.843403 | 0.000715 | 0.003738 | up-regulated gene |
| TMEM158 | 202.998 | 729.4477 | 1.845339 | 5.10E-07 | 2.30E-05 | up-regulated gene |
| SERPINH1 | 184.7244 | 664.6067 | 1.847127 | 7.95E-08 | 1.84E-05 | up-regulated gene |
| SLC7A5 | 295.6571 | 1067.835 | 1.852691 | 4.70E-06 | 8.07E-05 | up-regulated gene |
| EGR1 | 242.8894 | 881.6013 | 1.859827 | 6.68E-06 | 0.000102 | up-regulated gene |
| S100A4 | 402.1967 | 1460.345 | 1.860336 | 0.000152 | 0.00108 | up-regulated gene |
| TRIP13 | 117.3129 | 430.6343 | 1.876101 | 6.62E-08 | 1.84E-05 | up-regulated gene |
| PDGFRB | 193.618 | 711.6913 | 1.878039 | 2.92E-06 | 5.90E-05 | up-regulated gene |
| SERPINB5 | 292.6611 | 1076.135 | 1.878557 | 0.000186 | 0.001259 | up-regulated gene |
| NNMT | 66.6539 | 246.1496 | 1.884774 | 2.04E-07 | 1.84E-05 | up-regulated gene |
| LOC731049 | 351.904 | 1303.541 | 1.889182 | 1.96E-07 | 1.84E-05 | up-regulated gene |
| COL4A1 | 830.1056 | 3088.968 | 1.895758 | 9.64E-07 | 3.10E-05 | up-regulated gene |
| MYC | 150.301 | 560.355 | 1.898486 | 3.31E-07 | 2.01E-05 | up-regulated gene |
| IDO1 | 80.75084 | 301.5294 | 1.900749 | 0.000512 | 0.002841 | up-regulated gene |
| C10orf99 | 103.9693 | 388.5523 | 1.901952 | 0.003078 | 0.012115 | up-regulated gene |
| CD55 | 116.7607 | 438.3231 | 1.90844 | 2.20E-06 | 4.91E-05 | up-regulated gene |
| LCN2 | 2987.756 | 11221.84 | 1.909176 | 9.65E-05 | 0.000757 | up-regulated gene |
| RGS1 | 78.97231 | 299.7971 | 1.924568 | 5.33E-07 | 2.36E-05 | up-regulated gene |
| CDC25B | 185.1824 | 704.9342 | 1.928541 | 1.79E-07 | 1.84E-05 | up-regulated gene |
| CDCA5 | 189.0309 | 729.1862 | 1.947665 | 9.12E-08 | 1.84E-05 | up-regulated gene |
| IL1B | 81.15513 | 313.1948 | 1.948306 | 0.000241 | 0.001558 | up-regulated gene |
| SNHG6 | 426.3839 | 1655.016 | 1.95662 | 1.37E-07 | 1.84E-05 | up-regulated gene |
| CXCL1 | 125.0422 | 488.9821 | 1.967367 | 1.66E-05 | 0.000194 | up-regulated gene |
| CXCL5 | 67.14392 | 262.5859 | 1.967461 | 0.003378 | 0.012995 | up-regulated gene |
| KRT17P3 | 80.52503 | 315.984 | 1.972343 | 0.00088 | 0.004416 | up-regulated gene |
| BST2 | 319.0396 | 1286.371 | 2.0115 | 3.71E-06 | 6.93E-05 | up-regulated gene |
| MCM2 | 101.3616 | 408.9687 | 2.012479 | 4.37E-08 | 1.84E-05 | up-regulated gene |
| IFITM2 | 1529.339 | 6207.483 | 2.0211 | 1.71E-07 | 1.84E-05 | up-regulated gene |
| CTSL2 | 160.4841 | 652.298 | 2.023101 | 1.79E-06 | 4.32E-05 | up-regulated gene |
| THY1 | 160.2832 | 655.0099 | 2.030894 | 1.14E-06 | 3.37E-05 | up-regulated gene |
| H19 | 309.8482 | 1269.128 | 2.034204 | 6.18E-06 | 9.64E-05 | up-regulated gene |
| CXCL9 | 64.77319 | 265.403 | 2.034716 | 2.49E-05 | 0.000263 | up-regulated gene |
| C1QC | 130.2161 | 536.4484 | 2.042531 | 3.29E-06 | 6.38E-05 | up-regulated gene |
| PDZK1IP1 | 162.847 | 674.7017 | 2.050733 | 4.28E-05 | 0.000401 | up-regulated gene |
| COL7A1 | 145.3585 | 604.5752 | 2.056307 | 1.72E-06 | 4.24E-05 | up-regulated gene |
| IGF2BP3 | 58.49024 | 243.8508 | 2.059731 | 2.92E-06 | 5.90E-05 | up-regulated gene |
| CRCT1 | 70.04299 | 294.8175 | 2.07351 | 0.018129 | 0.049137 | up-regulated gene |
| CELSR3 | 85.68788 | 362.2529 | 2.079834 | 4.30E-07 | 2.14E-05 | up-regulated gene |
| RPS21 | 290.9638 | 1241.134 | 2.092747 | 2.04E-07 | 1.84E-05 | up-regulated gene |
| DEFB103B | 66.4379 | 285.2746 | 2.102273 | 0.004742 | 0.017045 | up-regulated gene |
| GJB2 | 420.2679 | 1836.036 | 2.127213 | 0.001304 | 0.006082 | up-regulated gene |
| CXCL10 | 122.0957 | 533.6136 | 2.127784 | 0.000248 | 0.0016 | up-regulated gene |
| LOC100132394 | 378.5624 | 1700.312 | 2.167197 | 0.000375 | 0.002229 | up-regulated gene |
| KIAA1199 | 91.07729 | 409.253 | 2.16783 | 7.80E-06 | 0.000114 | up-regulated gene |
| COL5A2 | 166.6635 | 750.6239 | 2.171152 | 6.32E-07 | 2.52E-05 | up-regulated gene |
| IER3 | 605.6095 | 2756.637 | 2.18645 | 1.37E-06 | 3.76E-05 | up-regulated gene |
| C1QB | 154.8362 | 710.3729 | 2.197833 | 1.33E-05 | 0.000165 | up-regulated gene |
| FOSB | 113.6791 | 522.6571 | 2.200898 | 1.73E-05 | 0.0002 | up-regulated gene |
| LOC651816 | 132.4253 | 615.5669 | 2.216737 | 7.26E-08 | 1.84E-05 | up-regulated gene |
| LOC441763 | 96.69255 | 451.2213 | 2.222358 | 0.000205 | 0.001363 | up-regulated gene |
| CYR61 | 105.1087 | 490.8575 | 2.223422 | 6.32E-07 | 2.52E-05 | up-regulated gene |
| TIMP1 | 1184.477 | 5645.389 | 2.252823 | 4.68E-07 | 2.23E-05 | up-regulated gene |
| RARRES1 | 80.14073 | 389.6409 | 2.281538 | 2.91E-07 | 1.98E-05 | up-regulated gene |
| APOC1 | 130.9018 | 640.2472 | 2.290144 | 2.16E-06 | 4.88E-05 | up-regulated gene |
| TNFRSF6B | 68.14947 | 336.2219 | 2.302639 | 1.64E-07 | 1.84E-05 | up-regulated gene |
| KLK6 | 134.5242 | 683.0537 | 2.344133 | 5.67E-05 | 0.000502 | up-regulated gene |
| IFITM3 | 1046.764 | 5380.974 | 2.361932 | 6.62E-08 | 1.84E-05 | up-regulated gene |
| COL1A2 | 296.6838 | 1540.345 | 2.376256 | 5.29E-06 | 8.66E-05 | up-regulated gene |
| SPRR2G | 57.32964 | 300.7523 | 2.391222 | 0.007454 | 0.024283 | up-regulated gene |
| GDF15 | 165.6249 | 872.2735 | 2.396861 | 1.43E-05 | 0.000174 | up-regulated gene |
| UBE2C | 144.6335 | 763.0326 | 2.399343 | 5.02E-08 | 1.84E-05 | up-regulated gene |
| SLC2A1 | 245.8557 | 1310.539 | 2.414277 | 8.86E-07 | 2.98E-05 | up-regulated gene |
| ISG15 | 433.4549 | 2311.311 | 2.414758 | 1.14E-05 | 0.000149 | up-regulated gene |
| MMP12 | 62.5322 | 335.7046 | 2.424521 | 9.99E-08 | 1.84E-05 | up-regulated gene |
| BAMBI | 216.8639 | 1217.996 | 2.489647 | 6.99E-05 | 0.000587 | up-regulated gene |
| HOXB8 | 53.90611 | 303.0943 | 2.491246 | 7.81E-07 | 2.82E-05 | up-regulated gene |
| IFITM1 | 374.5207 | 2110.733 | 2.494627 | 4.34E-06 | 7.68E-05 | up-regulated gene |
| LY6E | 572.2053 | 3249.963 | 2.505819 | 2.04E-07 | 1.84E-05 | up-regulated gene |
| LOC644695 | 75.72838 | 431.7369 | 2.511247 | 2.55E-07 | 1.95E-05 | up-regulated gene |
| CCL20 | 288.7127 | 1668.958 | 2.531241 | 0.000117 | 0.000879 | up-regulated gene |
| TRIB3 | 105.1552 | 610.6735 | 2.537881 | 5.10E-07 | 2.30E-05 | up-regulated gene |
| BGN | 702.0724 | 4113.135 | 2.550547 | 2.14E-07 | 1.84E-05 | up-regulated gene |
| MMP9 | 59.62602 | 361.7939 | 2.601154 | 7.60E-08 | 1.84E-05 | up-regulated gene |
| CNTNAP2 | 66.47961 | 413.7051 | 2.637619 | 0.000109 | 0.000834 | up-regulated gene |
| MMP3 | 101.0349 | 629.4353 | 2.639205 | 9.52E-05 | 0.000747 | up-regulated gene |
| MMP7 | 216.1146 | 1361.888 | 2.655739 | 0.007454 | 0.024283 | up-regulated gene |
| HOXB7 | 89.60473 | 570.8531 | 2.671473 | 1.37E-07 | 1.84E-05 | up-regulated gene |
| PPP1R1B | 194.7829 | 1262.284 | 2.696097 | 0.000364 | 0.002173 | up-regulated gene |
| CEBPB | 478.952 | 3127.456 | 2.707037 | 5.51E-08 | 1.84E-05 | up-regulated gene |
| CLDN1 | 134.1386 | 879.1651 | 2.71241 | 1.52E-06 | 3.96E-05 | up-regulated gene |
| THBS2 | 89.74835 | 588.6634 | 2.713486 | 8.50E-07 | 2.92E-05 | up-regulated gene |
| LOC645638 | 153.472 | 1017.52 | 2.72901 | 0.000301 | 0.001856 | up-regulated gene |
| TOP2A | 279.1815 | 1902.171 | 2.768372 | 6.93E-08 | 1.84E-05 | up-regulated gene |
| CDH3 | 71.7435 | 490.8252 | 2.774289 | 1.50E-07 | 1.84E-05 | up-regulated gene |
| UBD | 202.9145 | 1421.821 | 2.808796 | 8.02E-05 | 0.000653 | up-regulated gene |
| APOE | 129.3415 | 979.6264 | 2.921046 | 5.72E-06 | 9.12E-05 | up-regulated gene |
| MMP11 | 73.25429 | 568.0511 | 2.955036 | 1.25E-07 | 1.84E-05 | up-regulated gene |
| LOC400578 | 78.03933 | 634.8683 | 3.024184 | 0.002654 | 0.010744 | up-regulated gene |
| SPRR2A | 282.7005 | 2304.322 | 3.026996 | 0.011482 | 0.034219 | up-regulated gene |
| LOC100008589 | 283.2665 | 2325.41 | 3.037253 | 4.13E-05 | 0.00039 | up-regulated gene |
| LOC100132564 | 93.17483 | 774.0576 | 3.054429 | 3.45E-05 | 0.000341 | up-regulated gene |
| IL8 | 109.543 | 917.6472 | 3.066442 | 1.05E-06 | 3.26E-05 | up-regulated gene |
| LGALS7 | 126.3646 | 1060.403 | 3.068948 | 1.06E-05 | 0.000141 | up-regulated gene |
| LOC653499 | 73.11202 | 620.2758 | 3.084729 | 0.000205 | 0.001363 | up-regulated gene |
| SNORD3A | 245.7823 | 2093.01 | 3.090126 | 0.004563 | 0.016567 | up-regulated gene |
| LOC728910 | 98.25356 | 895.4423 | 3.188019 | 0.000133 | 0.000972 | up-regulated gene |
| CST1 | 56.05526 | 516.8337 | 3.204779 | 6.94E-08 | 1.84E-05 | up-regulated gene |
| MGC102966 | 102.5352 | 970.5706 | 3.242714 | 0.015495 | 0.043276 | up-regulated gene |
| COL1A1 | 236.9925 | 2318.973 | 3.290573 | 2.44E-07 | 1.93E-05 | up-regulated gene |
| GAGE12H | 64.88857 | 658.3767 | 3.342877 | 0.001361 | 0.006276 | up-regulated gene |
| KRT6B | 123.4704 | 1293.084 | 3.388578 | 0.008839 | 0.027907 | up-regulated gene |
| CNFN | 138.1738 | 1531.856 | 3.470725 | 0.003469 | 0.013271 | up-regulated gene |
| KRT17 | 152.8449 | 1781.926 | 3.543297 | 2.20E-06 | 4.91E-05 | up-regulated gene |
| LGALS7B | 100.1432 | 1168.953 | 3.545081 | 9.20E-05 | 0.000727 | up-regulated gene |
| GAGE2B | 65.6063 | 834.9274 | 3.669744 | 3.21E-05 | 0.000321 | up-regulated gene |
| SNORD3C | 100.519 | 1283.874 | 3.674963 | 3.10E-05 | 0.000313 | up-regulated gene |
| REG4 | 134.9331 | 1794.041 | 3.732897 | 0.004504 | 0.016376 | up-regulated gene |
| KRT16 | 64.059 | 862.104 | 3.750389 | 1.49E-05 | 0.000179 | up-regulated gene |
| PI3 | 360.5047 | 5667.928 | 3.974732 | 1.79E-06 | 4.32E-05 | up-regulated gene |
| LOC651397 | 66.65056 | 1196.009 | 4.165467 | 0.001591 | 0.00712 | up-regulated gene |
